# The efficacy of sodium hexafluoroaluminate (cryolite) for direct amidation of carboxylic acids

**DOI:** 10.1039/d5ra04566e

**Published:** 2025-09-18

**Authors:** Aman G. Singh, Jacob Briere, Jonah VanDer Kamp, P. Veeraraghavan Ramachandran

**Affiliations:** a Department of Chemistry, Purdue University West Lafayette IN 47907 USA chandran@purdue.edu

## Abstract

Direct amidation of carboxylic acids is a fundamental transformation in organic synthesis and plays a pivotal role in the construction of amide bonds that are ubiquitous in pharmaceuticals, natural products, and materials. Despite its importance, many of the reported catalytic methods rely on expensive, moisture-sensitive, or inefficient reagents, thereby limiting their practical utility. In this study, we introduce sodium hexafluoroaluminate (cryolite, Na_3_AlF_6_) as a cost-effective, recyclable, and environmentally benign catalyst for direct amidation. Using 10 mol% cryolite, a broad range of aromatic and aliphatic carboxylic acids were converted into the corresponding amides in moderate to high yields (46–97%). Notably, the method requires no specialized additives or chromatographic purification, further enhancing its synthetic practicality. Beyond amidation, cryolite also proved to be highly effective in esterification reactions, affording esters in up to 90–92% yield within 24 hours when employed in either toluene or the reacting alcohol as solvent. The catalyst is inexpensive on a per-mole basis, widely available, and easy to handle, and it can be recycled without significant loss of activity, underscoring its potential for sustainable applications. These attributes position cryolite as a robust and attractive alternative to conventional transition-metal-based catalysts. To the best of our knowledge, this work represents the first demonstration of cryolite as a catalyst in organic synthesis, providing a practical, scalable, and green approach to both amide and ester bond formation.

## Abstract 2

Direct amidation of carboxylic acids is a fundamental transformation in organic synthesis and plays a pivotal role in the construction of amide bonds that are ubiquitous in pharmaceuticals, natural products, and materials.

## Graphical abstract

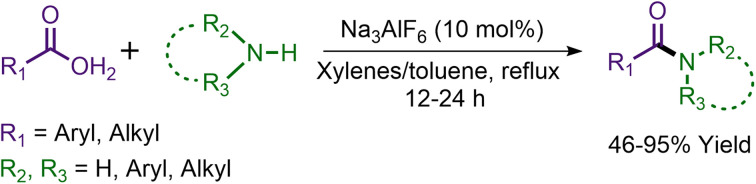

## Introduction

The carboxamide units in proteins are of fundamental significance in biological processes.^1^ Their relevance in the pharmaceutical realm also cannot be understated as they are constituents of a large percentage of FDA-approved drugs.^2^ Their synthesis *via* direct amidation of carboxylic acids is considered as one of the challenging reactions^3^ due to the drastic conditions required for the dehydration of intermediate ammonium carboxylates. Accordingly, amidation is often achieved by converting the acid to reactive derivatives, such as acid chlorides,^4^ or mixed carboxylic,^5^ sulfonyl,^6^ or phosphoryl anhydrides,^7^*etc.* Various coupling reagents, such as DCC,^8^ DIC,^9^ EDC,^10^ BOP,^11^ PyBOP,^12^ BOMI,^13^ CDI,^14^ HBTU,^15^ HOAt,^16^ EEDQ,^17^*etc.*, have also been employed for the dehydrative amidation. However, influenced by several key factors, including ready bulk availability, low cost, and efficiency, only a select few have been adopted for industrial-scale amide production. Furthermore, the difficulty in handling and removal of by-products are also critical factors contributing to the lack of enthusiasm for several coupling agents.^1^ Such factors affecting the manufacturing environment necessitates more suitable reagents, particularly catalysts, for the direct amidation of acids. Leading the main group element-catalysed amidation protocols are boron-^18–21^ and silicon-based^22^ catalysts.

**Scheme 1:**
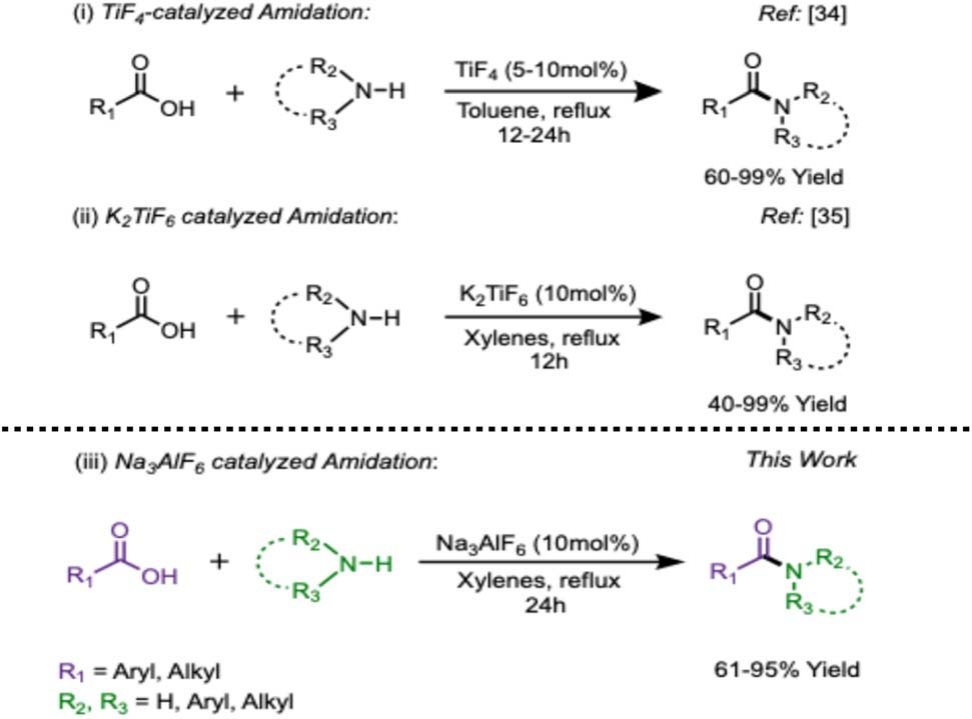
Comparison of fluoride mediated amidation.

Over the years, our group has made significant advances in developing novel direct amidation protocols employing catalytic borane–amines, such as borane–ammonia,^23^ borane–triethylamine,^23^ borane–pyridine,^24^ or utilizing borane–amines as dual-purpose (catalyst and amine carrier) reagents.^25^ In recent years, transition metal salts—particularly those of Ti,^26^ Nb,^27^ Fe,^28^ Zn,^29^ Zr,^30^ Rh,^31^ Hf,^32^ Ta,^33^ In,^34^—have been introduced for direct amidation, while organic salts such as tropylium,^35^ pyridinium,^36^ imidazolium,^37^ have also been well established. Our recent efforts have led to the examination of metal fluorides as amidation catalysts, and we have described the catalytic ability of TiF_4_ for direct amidation (Scheme 1).^38^ Despite its considerable advantages, the challenges associated with the handling of TiF_4_ led us to explore other viable candidates resulting in the introduction of low-cost^39^ potassium hexafluorotitanate (K_2_TiF_6_) as a catalyst for the direct amidation of carboxylic acids marking the first application of this hexafluoride salt in organic reactions (Scheme 1).^40^ We had compared^40^ sodium hexafluorosilicate (Na_2_SiF_6_), tetrafluoroammonium borate (NH_4_BF_4_), and sodium hexafluoroaluminate (cryolite) for a typical amidation. The results showed complete conversion, albeit in less than satisfactory yields of the product amides (Table 1, entries 1–3). The yields achieved with cryolite encouraged to optimize this reagent for direct amidation due to the low-cost and natural availability of this aluminate salt. This study has led to the application of cryolite as an efficient direct amidation catalyst. Further examination revealed that this salt is efficient for direct esterification as well. The details follow.

**Table 1:** Optimizations of fluoride salt catalysts for amidation

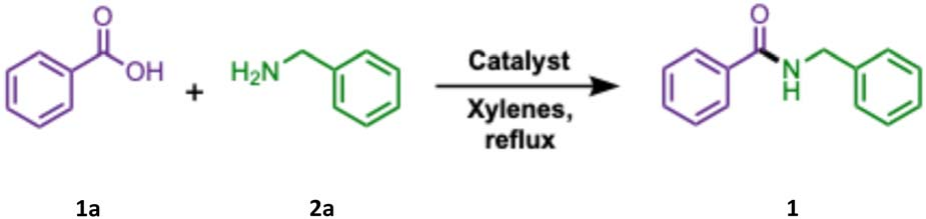						
Entry	Catalyst	Catal. loading (mol%)	Acid equiv.	Solvent	Time, h	Yield^a^, %
1	NH_4_BF_4_	10	1.1	Xylenes	12	45
2	Na_2_SiF_6_	10	1.1	Xylenes	12	43
3	Na_3_AlF_6_	10	1.1	Xylenes	12	46
4	Na_3_AlF_6_	100	1.1	Xylenes	12	50
5	Na_3_AlF_6_	10	1.1	Xylenes	24	56
6	Na_3_AlF_6_	100	1.1	Xylenes	24	69
7	Na_3_AlF_6_	10	1.25	Xylenes	24	79
8	Na_3_AlF_6_	50	1.25	Xylenes	24	57
9	Na_3_AlF_6_	10	1.2	Xylenes	24	77
10	Na_3_AlF_6_	10	1.2	Xylenes	12	66
11	Na_3_AlF_6_	10	1	Xylenes	24	67
12	Na_3_AlF_6_	10	1	Xylenes	24	72^b^
13	Na_3_AlF_6_	10	1.2	Xylenes	24	59^c^
14	Na_3_AlF_6_	10	1.2	Toluene	24	30
15	Na_3_AlF_6_	10	1.2	Water	24	2
16	Na_3_AlF_6_	10	1.2	Ethyl acetate	24	50
17	Na_3_AlF_6_	10	1.2	Dioxane	24	52
18	Na_3_AlF_6_	10	1.2	DCE	24	2^d^
19	Na_3_AlF_6_	10	1.2	Neat	24	19

a Isolated yields.

b 1.2 equiv. of amine used.

c Pyridine was used as an additive.

d Chloroethyl ester was formed as the major product.

## Results and discussions

The optimization of cryolite-mediated amidation was initiated by repeating the typical amidation of benzoic acid (1a) with benzylamine (2a) as the model substrates in xylenes solvent under reflux for 12 h.^40^ Unexpectedly, when the preparation of benzyl benzamide (1) was attempted at a 5 mmol scale rather than the previously reported 1 mmol scale, the yield decreased from 64% (ref. 35) to 46% (Table 1, entry 3). The yield slightly increased to 50% when stoichiometric quantities of the catalyst were used for 12 h (entry 4). Maintaining the catalyst loading at 10 mol% and extending the reaction time to 24 h, increased the yield to 56% (entry 5). Encouraged by this, we focused on the carboxylic acid and amine equivalencies, while maintaining this condition. Substantial benefit was observed when 1.25 equiv. of benzoic acid (1a) was used and a 79% isolated yield of 1 was achieved (entry 7). Under the same vein, when the catalyst loading was increased to 50 mol%, again, the yield dropped to 57% (entry 8). Lowering the acid equiv. to 1.2 achieved an almost similar result with an isolated yield of 77% (entry 9) within 24 h of reflux. With this ratio of acid and amine, decreasing the reaction time to 12 h displayed a lower yield of 66% (entry 10). Equimolar acid and amine (entry 11) and an excess of amine (1.2 equiv., entry 12) was not particularly effective.

**Scheme 2:**
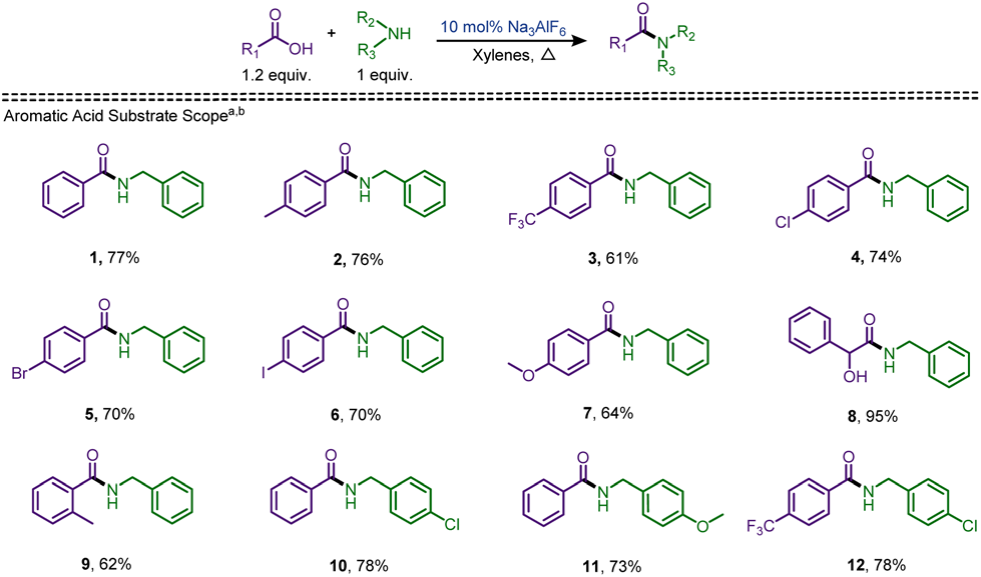
Scope of amidation of aryl carboxylic acids. ^*a*^0.1 equiv. of cryolite in refluxing xylenes. ^*b*^Isolated yield. ^*c*^Refluxed in toluene. ^*d*^Refluxed in toluene with 0.5 equiv. of cryolite.

**Scheme 3:**
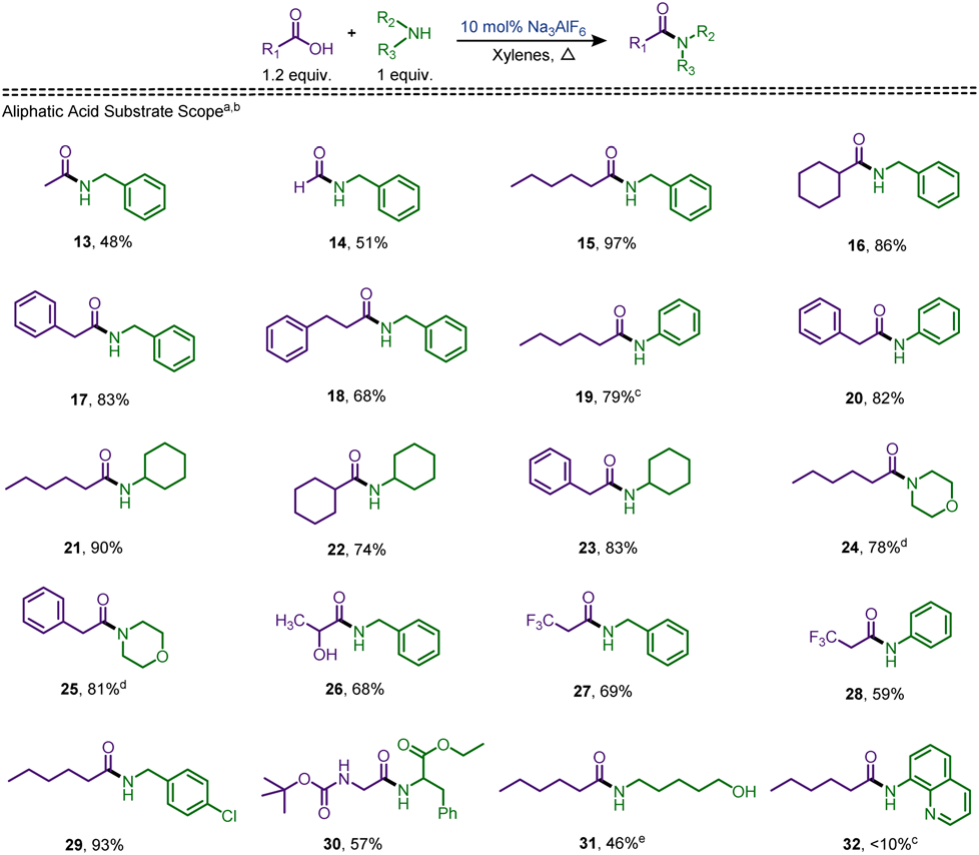
Scope of amidation of alkyl carboxylic acids. ^*a*^0.1 equiv. of cryolite in refluxing xylenes. ^*b*^Isolated yield. ^*c*^Refluxed in toluene. ^*d*^In toluene with 0.5 equiv. of cryolite. ^*e*^In toluene with 1.0 equiv. of cryolite.

Refluxing xylenes proved to be the ideal solvent to carry out the reactions (entries 14–19), though toluene was the better solvent for the preparation of tertiary amides (*vide infra*). Having determined the optimal conditions (entry 9) for cryolite-catalysed amidation, the substrate scope was examined using a range of acids and amines (Schemes 2 and 3). Initially, aromatic acids with electron-donating or -withdrawing groups was examined in conjunction with aliphatic amines (Scheme 2). 4-Methylbenzoic acid reacted with benzylamine (2a) to yield the product amide 2 in 76% yield. Bulky electron-withdrawing groups attached to the carboxylic acids, such as in 4-(trifluoromethyl)benzoic acid lowered the isolated yield to 61%. An increase in yield was observed when halogen-containing, *p*-chlorobenzoic acid, *p*-bromobenzoic acid, and *p*-iodobenzoic acid, were reacted with benzylamine providing carboxamides 4, 5, and 6 in 74%, 70%, and 70% isolated yields, respectively. These results followed our reported trend of activated aromatic acids performing better with the chosen catalyst.^40^ When electron-donating 4-methoxy group containing benzoic acid was examined, the product amide (7) was isolated in 64% yield, comparable to 3. An α-hydroxy acid, mandelic acid, provided a near quantitative yield when coupled with benzylamine. Compared to the formation of carboxamide 2, when *ortho*-toluic acid was treated with benzylamine, a yield of 62% was observed for amide 9. Upon reaction of *p*-chlorobenzylamine and *p*-methoxybenzylamine with benzoic acid (1a), the corresponding amides 10 and 11 were isolated in 78% and 73% yields, respectively. *p*-Chlorobenzylamine was also tested against 4-(trifluoromethyl)benzoic acid to give the resultant carboxamide (12) in 78% yield. These results are similar to the model amide 1, concluding that the changes in the acid moiety revealed noticeable effect.

In the subsequent series (Scheme 3), aliphatic acids were tested in reactions with aromatic and aliphatic amines. Acetic acid and formic acid reacted with benzylamine, yielding the corresponding amides 13 and 14, in 48% and 51% yields respectively. However, when the same amine was reacted with hexanoic acid, cyclohexanecarboxylic acid, phenylacetic acid, and phenylpropanoic acid, the resulting amides, 15, 16, 17, and 18 were obtained in the yields of 97%, 86%, 83%, and 68% respectively (Scheme 2). When an aromatic amine, aniline, was treated with hexanoic acid and phenylacetic acid, the carboxamides were isolated in appreciable yields of 79% and 82%, respectively. The reaction of hexanoic acid with aniline in refluxing xylenes provided poor yield (46%) for the corresponding amide 19. However, this reaction in toluene as the solvent provided a satisfactory 79% yield of 19. No solvent change was required for the reaction of aniline with phenylacetic acid, and the corresponding amide 20 was obtained in 82% yield under the optimized conditions. When cyclohexylamine was treated with hexanoic, cyclohexanecarboxylic, and phenylacetic acids, the corresponding amides 21, 22, and 23 were achieved in 90%, 74%, and 83% yields, respectively. After evaluating the secondary amide formation with primary amines, attention was turned to the preparation of tertiary amides. Initially, under the optimized conditions, morpholine failed to provide satisfactory results when treated with both hexanoic and phenylacetic acids.

Based on our previous reports,^40^ we hypothesized that a lower temperature may be beneficial for obtaining suitable yields of amides and toluene was used as the solvent. With this modification, we observed a significant increase in the yields when the catalyst loading was increased to 50 mol%. Thus, the amides 24, and 25 were isolated in 78%, and 81% respectively. Based on the favoured amidation of mandelic acid, an aliphatic α-hydroxy acid, lactic acid, was also examined.

Reaction with 2a provided the corresponding carboxamide, 26, in 68% isolated yield. To expand the substrate scope, we also synthesized trifluoropropanamides 27 and 28 in 69% and 59% yields, respectively. To test the effect of a change in the amine moiety, amide 29 was synthesised, which was obtained in an appreciable yield of 93%. In addition, Boc-glycine and ethyl phenylalaninate were reacted to produce the resultant dipeptide 30 in 57% yield. Modified conditions (100 mol% cryolite) were required to prepare amide 31 (46%) from an amino alcohol, since the optimized method (10 mol%) provided a 52 : 48 mixture of amide and ester products. Sub-satisfactory results were obtained upon exploring the scope of hindered amines, 8-aminoquinoline, with the resultant amide 32 isolated in a poor 8% yield. This result may be attributed to the bulk of the amine or the interference of the non-participating nitrogen atom with the catalyst, diminishing its activity.

**Scheme 4:**
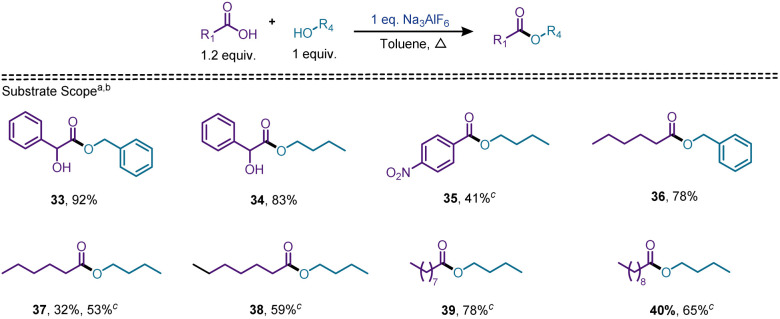
Scope of esterification. ^*a*^1 equiv. of cryolite in refluxing toluene. ^*b*^Isolated yield. ^*c*^Reaction using alcohol as the solvent.

**Scheme 5:**
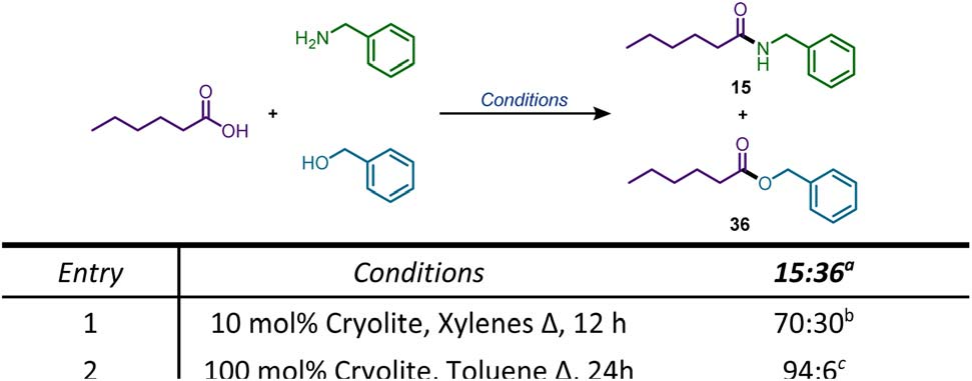
Competition reaction. ^*a*^Ratios based on ^1^H-NMR. ^*b*1^H-NMR ratio of unreacted alcohol to 36 is 70 : 30. ^*c*1^H-NMR ratio of unreacted alcohol to 36 is 93 : 7.

The formation of ester product from 5-aminopentanol prompted us to examine the efficacy of cryolite for esterification of carboxylic acids, when the corresponding esters were realized in good yields (up to 92%). We chose mandelic acid as the model carboxylic acid for optimization purposes, since it provided excellent results for amidation. Unfortunately, new reaction conditions had to be developed, since the previous optimized conditions failed to provide esters in good yields. We observed that when the cryolite loading was increased from 10 mol% to 100 mol%, the desired product (33) was isolable in 92% yield using benzyl alcohol. Toluene was the preferred choice of solvent with the duration of the reaction unchanged (24 h). Scheme 4 shows the substrate scope for esterification. Mandelic acid was tested against benzyl alcohol, and *n*-butanol, to give the corresponding esters in 97%, and 83% yields respectively. Benzoic acid and substituted benzoic acids tested poorly against these alcohols, apart from nitrobenzoic acid, which provided an acceptable yield of 41% for the ester 35. We then moved to aliphatic acids, amongst which hexanoic acid gave a good yield of 78% when reacted with benzyl alcohol, but when tested against *n*-butanol, provided a poor yield of the ester product. A significant number of esterification protocols in the literature use alcohol as the solvent.^41^ Based on this, we modified our procedure and used *n*-butanol as the solvent and the yield of the ester 37 increased from 32% to 53%. Esters 38, 39, and 40 were also synthesized in 59%, 78%, and 65% yields respectively, using the alcohol as the solvent. To determine the selectivity of carboxamide *versus* ester formation, we carried out two competition reactions (Scheme 5). Using the amidation conditions (Schemes 2 and 3), we reacted hexanoic acid with benzylamine and benzyl alcohol in the same pot and observed an amidation to esterification ratio of 70 : 30. Surprisingly, when the above reaction was carried out under esterification conditions (Scheme 4), the ratio of the amide to the ester product increased to 94 : 6 favouring amidation.

During our experiments, we observed rapid settling of the cryolite catalyst upon quenching the reaction mixture. This observation prompted us to investigate the catalyst's recyclability for amidation. Owing to its consistently high yield, compound 15 was selected as the model substrate for the recycling study. We conducted the reaction over five consecutive cycles in the same pot using fresh equivalents of the starting materials for each run, without any intermediate purification of the catalyst (see SI Table S1).

No reduction in yield was observed in the first recycle (94% = 97 × 0.97). However, subsequent cycles showed a slight decrease in yields. Upon completion of the fifth cycle, the overall isolated yield dropped to 80%. These results highlight the catalyst's potential for reuse with minimal loss in activity, underscoring its practical utility in sustainable amidation processes. Further optimization of the recycling study was not conducted.

**Fig. 1:**
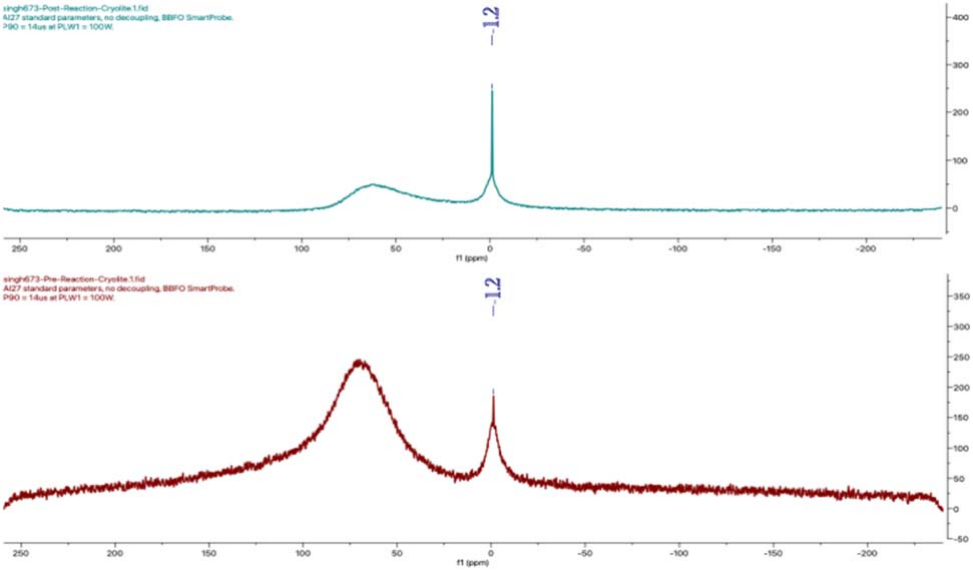
^27^Al NMR spectra of Na_3_AlF_6_ before (red) and after (blue) the reaction.

To assess whether cryolite undergoes any compositional changes during the reaction, pre- and post-reaction catalysts were analysed by powder X-ray diffraction (see SI for details). The diffraction patterns indicated no significant alteration, supporting the catalyst's stability and recyclability. Furthermore, ^27^Al NMR spectrum of the catalyst before and after the reaction exhibited a characteristic peak at −1.2 ppm, attributed to the hexacoordinated aluminium species.^42^ The absence of any downfield shift suggests that the coordination environment and oxidation sate of aluminium remain unaltered throughout the reaction,^43^ consistent with its role as a true heterogeneous catalyst. In view of the above, defining the precise role of cryolite in carbonyl activation for amidation remains inherently difficult. Dissociation to aluminium fluoride appears unlikely, as NMR studies display consistent spectral features for cryolite throughout the reaction, and prior work^44^ has explicitly negated the possibility of such dissociation (Fig. 1).

**Fig. 2:**
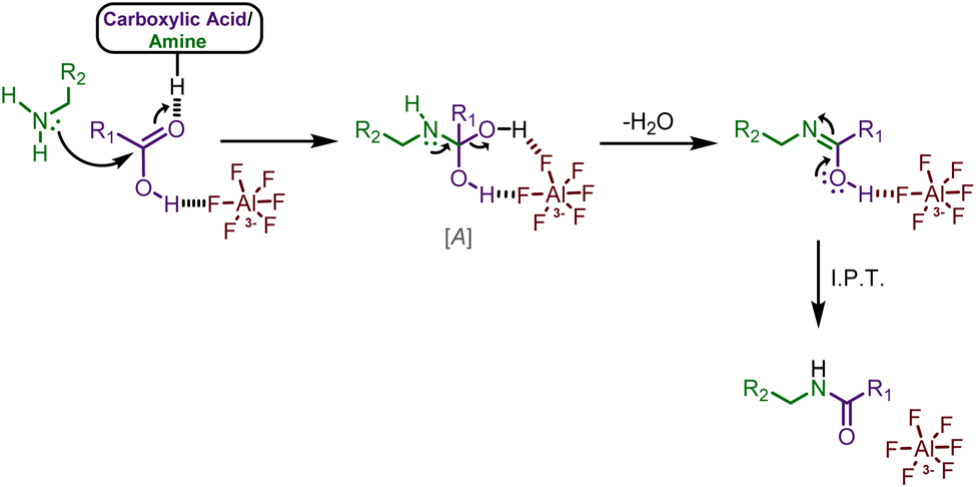
Plausible mechanism of cryolite-mediated amidation.

It is well established that a fluorine atom can engage in strong hydrogen-bonding interactions.^45^ On the basis of reported literature on hydrogen bonding interaction of carboxylic acids for amidation,^46^ we have invoked a fluorine-mediated hydrogen bonding mechanism, with similar strength to weak covalent bonds,^45^*via* the formation of intermediate [A]^46^ in Fig. 2.

To shed light upon the environmental appeal, we calculated certain green chemistry metrics^47^ for the preparation of amide 15 and compared them to recently published green protocols (Table 2). Our method compared well with these reports with an atom economy of 83%, atom efficiency of 81% and reaction mass efficiency of 81% (see SI for calculation details).

**Table 2:** Green chemistry metrics

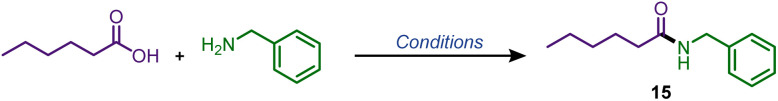			
Metrics	This method	Ref. 49	Ref. 50
Yield	97%	98%	65%
Atom economy	83%	92%	80%
Atom efficiency	81%	90%	52%
Reaction mass efficiency	81%	90%	52%

## Conclusions

In conclusion, a new direct amidation protocol has been reported using catalytic Na_3_AlF_6_ to yield secondary amides in refluxing xylenes and tertiary amides in refluxing toluene. This method has proved to be efficient in providing good to high yields for a range of aromatic and aliphatic substrates without the need for any chromatographic purification. Apart from being efficacious in amidation, cryolite has proved to be fruitful in esterification as well, with yields up to 90–92%, using 1 equiv. of the catalyst in either toluene or the reacting alcohol in 24 h. Cryolite is widely available, easy to handle, with an attractive pricing/mole,^48^ and its demonstrated recyclability makes it an excellent alternative to the many expensive and currently used catalysts. To the best of our knowledge, this procedure marks the first application of cryolite as a catalyst in organic synthesis.

## Author contributions

P. V. Ramachandran: funding acquisition, conceptualization, project administration, review and editing; A. G. Singh: data curation, investigation, methodology, validation, writing; J. Briere: data curation, investigation, methodology; J. V. Kamp: data curation.

## Conflicts of interest

There are no conflicts to declare.

## Supplementary Material

RA-015-D5RA04566E-s001

## Data Availability

The data supporting this article have been included as part of the SI. Supplementary information: Optimization details, experimental procedures, product characterization, and ^1^H, ^13^C, and ^19^F NMR spectra of products. See DOI: https://doi.org/10.1039/d5ra04566e.
