# Supplementary material for: The efficacy of sodium hexafluoroaluminate (cryolite) for direct amidation of carboxylic acids
Source: RSC Adv. 2025 Sep 18;15(41):34122–7. doi: 10.1039/d5ra04566e (PMC12444625; doi:10.1039/d5ra04566e)
Supplement: RA-015-D5RA04566E-s001 [file RA-015-D5RA04566E-s001.pdf]

Supplementary Information (SI) for RSC Advances.
This journal is © The Royal Society of Chemistry 2025

The Efficacy of Sodium Hexafluoroaluminate (Cryolite) for Direct Amidation of Carboxylic Acids

Aman G. Singh, Jacob Briere, Jonah VanDerKamp, and P. Veeraraghavan Ramachandran*

Herbert C. Brown Center for Borane Research, Department of Chemistry, Purdue University, West Lafayette, Indiana 47907, United States

E-mail: chandran@purdue.edu

**Table of Contents:**	**Page:**
**1) Experimental Section**	**S2-S3**
**3) Catalyst Recyclability (SI Table-2)**	**S4**
**4) Product Characterization**	**S5-S14**
**5) References**	**S15**
**6) NMR Data**	**S16-S60**
**7) Green Chemistry Metrics**	**S61-S62**

Experimental Section:

General Information:

Unless otherwise noted, all additions were carried out under open air conditions. ^{11}B, ^{13}C and ^{1}H NMR spectra were recorded at room temperature, on a Bruker 300 MHz or Bruker 400 MHz NMR spectrophotometer as stated. Data are reported as: δ value, multiplicity (*s*=singlet, *d*=doublet, *t*=triplet, *q*=quartet, *p*=pentet, *h*=hextet, *m*=multiplet, *br*=broad) and integration. All solvents for routine isolation of products were reagent-grade. Amines, carboxylic acids, and catalysts were purchased from Sigma-Aldrich and/or Oakwood Chemicals and used without further purification. All reactions requiring heat were brought to temperature using an oil bath and heated stir plate. The cryolite powder prior to the reaction, and the cryolite isolated post-reaction were measured using a XRDynamic 500 diffractometer from Anton Paar using Cu Kα (λ = 1.5406 Å). The samples for XRD measurements were mounted on the fixed sample stage and scanned from 5° to 70° with a step size of 0.01° and a time per step of 20 s.

General procedure for the preparation of amides:

A 100 ml round bottom flask containing a magnetic stir-bar was charged with carboxylic acid (6 mmol, 1.2 eq) and xylenes (5 mL, 1 M with respect to amine). To this stirring mixture, cryolite ((0.5 mmol, 0.1 eq.) or (2.5 mmol, 0.5 eq.)) was introduced followed by addition of the respective amine (5 mmol, 1 eq.). A reflux condenser was attached to the flask and the reaction mixture was brought to reflux, using an oil bath. After completion (24 hours) the reaction mixture was cooled down, diluted with methanol and condensed via rotary evaporation. The crude mixture was diluted with dichloromethane (15mL) and transferred to a separatory funnel. The mixture was first washed with 3M HCl (2 x 10 mL) and then the separated organic layer was further washed with 3M sodium hydroxide solution (2 x 10 mL). The resultant organic layer was dried with sodium sulfate, filtered through cotton, and condensed via rotary evaporation following by drying *in vacuo* for 12 h.

Procedure for the preparation of esters using toluene as solvent:

A 100 ml round bottom flask containing a magnetic stir-bar was charged with carboxylic acid (6 mmol, 1.2 eq), toluene (5 mL, 1 M with respect to alcohol) and cryolite (5 mmol, 1 equiv.). To this stirring mixture, alcohol (5 mmol, 1 eq.) was introduced, and the heterogeneous mixture was then brought to reflux, using an oil bath. After completion (24 hours) the reaction mixture was cooled down, diluted with methanol and condensed via rotary evaporation. The crude mixture was diluted with dichloromethane (15mL) and transferred to a separatory funnel. The mixture was first washed with 3M HCl (2 x 10 mL) and then the separated organic layer was further washed with 3M sodium

hydroxide solution (2 x 10 mL). The resultant organic layer was dried with sodium sulfate, filtered through cotton, and condensed via rotary evaporation following by drying *in vacuo* for 12 h.

Procedure for the preparation of esters using alcohol as solvent:

A 100 ml round bottom flask containing a magnetic stir-bar was charged with carboxylic acid (5 mmol, 1 eq), alcohol (5 mL, 1 M with respect to carboxylic acid) and cryolite (5 mmol, 1 equiv.). This heterogeneous mixture was then brought to reflux, using an oil bath. After completion (24 hours) the reaction mixture was cooled down, diluted with hexanes and condensed via rotary evaporation. The crude mixture was diluted with dichloromethane (15mL) and transferred to a separatory funnel. The mixture was first washed with 3M HCl (2 x 10 mL) and then the separated organic layer was further washed with 3M sodium hydroxide solution (2 x 10 mL) (*Note*: if the organic layer appears to not be *clear* after the base wash, another acid wash is recommended!) . The resultant organic layer was dried with sodium sulfate, filtered through cotton, and condensed via rotary evaporation following by drying *in vacuo* for 12 h.

<u>Catalyst Recyclability Experiment:</u> SI Table 1

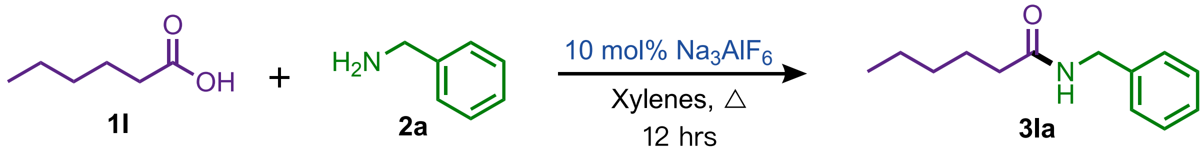

Number of Cycles:	Overall Yield (Isolated)
1st Cycle (Fresh)	97%
2nd Cycle	94%
3rd Cycle	86%
4th Cycle	85%
5th Cycle	80%

<u>Note:</u>

All recycling experiments were carried in one pot, i.e.: once the reaction time was completed, fresh equivalents of carboxylic acid and amine were added to the same reaction flask without altering the amount of cryolite.

<u>XRD Data:</u>

Pre-Reaction Cryolite:

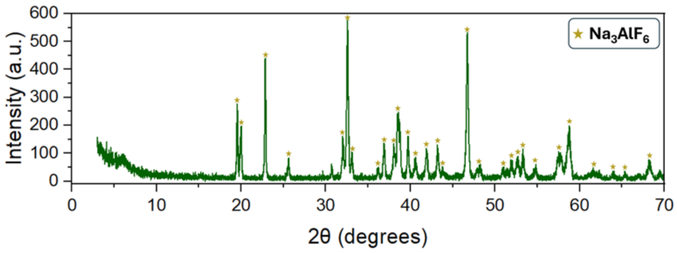

Post-Reaction Cryolite:

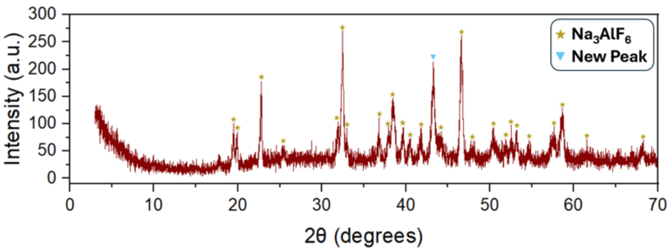

<u>Note</u>: The new observed peak at 43° may have risen due to the product mass from the reaction.

Pre-reaction cryolite XRD is in agreement with the previous report.[1]

Characterization of Amide Products:

N-benzylbenzamide (1)

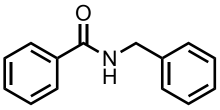

Yield 77%, Mass 0.81g, White Solid.

^{1}H NMR (300 MHz, $CDCl_3$): δ 7.85 – 7.75 (*m,* 2H), 7.55 – 7.28 (*m,* 8H), 6.42 (*s,* 1H), 4.66 (*d, J* = 5.7 Hz, 2H).
^{13}C {^{1}H} NMR (75 MHz, $CDCl_3$) δ 167.4, 138.2, 134.4, 131.6, 128.8, 128.6, 127.9, 127.6, 127.0, 44.1
Characterisation is in agreement with previous reports of this compound. [2]

N-benzyl-4-methylbenzamide (2)

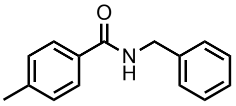

Yield 76%, Mass 0.86g, Pale Orange Solid

^{1}H NMR (300 MHz, $CDCl_3$) δ 7.75 – 7.63 (*m,* 2H), 7.41 – 7.16 (*m,* 7H), 6.41 (*s,* 1H), 4.63 (*d, J* = 5.7 Hz, 2H), 2.39 (*s,* 3H).
^{13}C {^{1}H} NMR (75 MHz, $CDCl_3$) δ 167.3, 142.0, 138.3, 131.5, 129.3, 128.8, 127.9, 127.6, 127.0, 44.1, 21.4.
Characterisation is in agreement with previous reports of this compound. [2]

N-benzyl-4-(trifluoromethyl)benzamide (3)

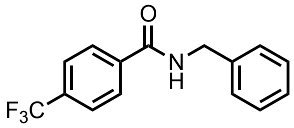

Yield 61%, Mass 0.85g, White Solid

^{1}H NMR (300 MHz, $CDCl_3$) δ 7.90 (d*t, J* = 8.0, 0.8 Hz, 2H), 7.73 – 7.65 (*m,* 2H), 7.39 – 7.28 (*m,* 5H), 6.48 (*s,* 1H), 4.66 (*d, J* = 5.6 Hz, 2H).
^{13}C {^{1}H} NMR (75 MHz, $CDCl_3$) δ 166.1, 137.7, 128.9, 128.0, 127.9, 127.5, 125.7 (*d, J* = 3.4 Hz), 44.4
^{19}F NMR (282 MHz, $CDCl_3$) δ -62.97.
Characterisation is in agreement with previous reports of this compound. [2]

N-benzyl-4-chlorobenzamide (4)

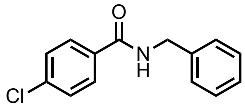

Yield 74%, Mass 0.91g, White Solid

^{1}H NMR (300 MHz, CDCl3) δ 7.72 (*d, J* = 8.7 Hz, 2H), 7.45 – 7.23 (*m,* 7H), 6.50 (*s,* 1H), 4.62 (*d, J* = 5.7 Hz, 2H).
^{13}C {^{1}H} NMR (75 MHz, $CDCl_3$) δ 166.3, 138.0, 137.8, 132.7, 128.8, 128.4, 127.9, 127.7, 44.2
Characterisation is in agreement with previous reports of this compound. [2]

N-benzyl-4-bromobenzamide (5)

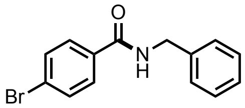

Yield 70%, Mass 1.01g, Off-white Solid.

^{1}H NMR (300 MHz, $CDCl_3$) δ 7.65 (*d, J* = 8.6 Hz, 2H), 7.55 (*d, J* = 8.5 Hz, 2H), 7.41 – 7.29 (*m,* 5H), 6.49 (*s,* 1H), 4.61 (*d, J* = 5.6 Hz, 2H).
^{13}C {^{1}H} NMR (75 MHz, $CDCl_3$) δ 166.4, 137.9, 133.2, 131.8, 128.9, 128.6, 128.0, 127.8, 126.3, 44.2
Characterisation is in agreement with previous reports of this compound.[2]

N-benzyl-4-iodobenzamide (6)

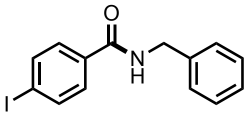

Yield 70%, Mass 1.17g, Off-white Solid.

^{1}H NMR (300 MHz, DMSO) δ 9.22 (*t, J* = 6.0 Hz, 1H), 7.98 (*d, J* = 8.4 Hz, 2H), 7.80 (*d, J* = 8.5 Hz, 2H), 7.51 – 7.29 (*m,* 5H), 4.59 (*d, J* = 6.0 Hz, 2H).
^{13}C {^{1}H} NMR (75 MHz, DMSO) δ 166.0, 140.0, 137.7, 134.2, 129.7, 128.8, 127.7, 127.2, 99.3, 43.1
Characterisation is in agreement with previous reports of this compound.[2]

N-benzyl-4-methoxybenzamide (7)

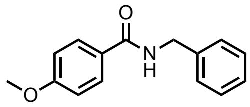

Yield 64%, Mass 0.76g, White Solid.

^{1}H NMR (300 MHz, $CDCl_3$): δ 7.76 (*d, J* = 8.8 Hz, 2H), 7.39 – 7.23 (*m,* 5H), 6.96 – 6.86 (*m,* 2H), 6.38 (*s,* 1H), 4.63 (*d, J* = 5.6 Hz, 2H), 3.84 (*s,* 3H)
^{13}C {^{1}H} NMR (75 MHz, $CDCl_3$): δ 166.9, 162.2, 138.4, 128.8, 127.9, 127.6, 126.7, 113.8, 55.4, 44.1
Characterisation is in agreement with previous reports of this compound. [3]

***N*-benzyl-2-hydroxy-2-phenylacetamide (8)**

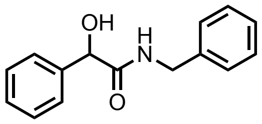

Yield 93%, Mass 1.12g, Yellow Solid.
^{1}H NMR (300 MHz, $CDCl_3$) δ 7.45 – 7.24 (*m,* 8H), 7.18 (d*d, J* = 7.7, 1.9 Hz, 2H), 6.53 (*s,* 1H), 5.05 (*d, J* = 2.5 Hz, 1H), 4.43 (d*d, J* = 5.9, 4.2 Hz, 2H), 3.69 (*d, J* = 3.4 Hz, 1H).
^{13}C {^{1}H} NMR (75 MHz, $CDCl_3$) δ 172.1, 139.4, 137.7, 128.9, 128.7, 127.6, 126.9, 74.3, 43.5
Characterisation is in agreement with previous reports of this compound. [4]

N-benzyl-2-methylbenzamide (9)

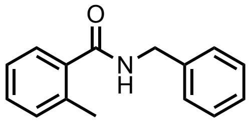

Yield 62%, Mass 0.70g, Brown Solid.

^{1}H NMR (300 MHz, $CDCl_3$) δ 7.44 – 7.11 (*m,* 9H), 6.06 (*s,* 1H), 4.62 (*d, J* = 5.7 Hz, 2H), 2.46 (*s,* 3H).
^{13}C {^{1}H} NMR (75 MHz, $CDCl_3$) δ 169.9, 138.2, 131.0, 130.0, 128.8, 127.9, 127.6, 126.7, 125.7, 43.9, 19.9.
Characterisation is in agreement with previous reports of this compound. [4]

***N*-(4-chlorobenzyl)benzamide (10)**

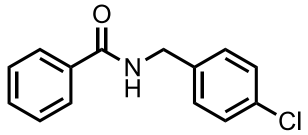

Yield 78%, Mass 0.96g, White Solid.

^{1}H NMR (300 MHz, $CDCl_3$): δ 7.84 – 7.74 (*m,* 2H), 7.57 – 7.38 (*m,* 3H), 7.35 – 7.24 (*m,* 4H), 6.52 (*s,* 1H), 4.60 (*d, J* = 5.8 Hz, 2H).
^{13}C {^{1}H} NMR (75 MHz, $CDCl_3$) δ 167.4, 136.8, 134.2, 133.4, 131.7, 129.2, 128.9, 128.7, 127.0, 43.4
Characterisation is in agreement with previous reports of this compound. [5]

***N*-(4-methoxybenzyl)benzamide (11)**

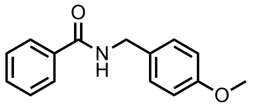

Yield 73%, Mass 0.88g, Brown Solid.

^{1}H NMR (300 MHz, $CDCl_3$) δ 7.81 – 7.74 (*m,* 2H), 7.54 – 7.35 (*m,* 3H), 7.31 – 7.23 (*m,* 2H), 6.92 – 6.84 (*m,* 2H), 6.46 (*s,* 1H), 4.56 (*d, J* = 5.6 Hz, 2H), 3.79 (*s,* 3H).
^{13}C {^{1}H} NMR (75 MHz, $CDCl_3$) δ 167.3, 159.1, 134.5, 131.5, 130.3, 129.3, 128.6, 127.0, 114.2, 55.3, 43.6.
Characterisation is in agreement with previous reports of this compound.[6]

***N*-(4-chlorobenzyl)-4-(trifluoromethyl)benzamide (12)**

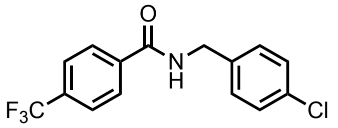

Yield 78%, Mass 1.23g, White Solid.

^{1}H NMR (300 MHz, $CDCl_3$) δ 7.89 (dd*t, J* = 7.8, 1.6, 0.9 Hz, 2H), 7.77 – 7.64 (*m,* 2H), 7.39 – 7.23 (*m,* 4H), 6.48 (*s,* 1H), 4.62 (*d, J* = 5.8 Hz, 2H).
^{13}C {^{1}H} NMR (75 MHz, $CDCl_3$) δ 166.1, 136.3, 133.7, 129.3, 129.0, 127.5, 125.7, 43.6
^{19}F NMR (282 MHz, $CDCl_3$) δ -62.99.
Characterisation is in agreement with previous reports of this compound. [7]

N-benzylacetamide (13)

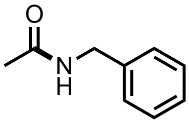

Yield 48%, Mass 0.36g, Brown Solid.

^{1}H NMR (300 MHz, $CDCl_3$) δ 7.32 – 7.11 (*m,* 5H), 6.08 (*s,* 1H), 4.31 (*d, J* = 5.7 Hz, 2H), 1.90 (*s,* 3H).
^{13}C {^{1}H} NMR (75 MHz, $CDCl_3$) δ 170.1, 138.3, 128.7, 127.8, 127.5, 43.7, 23.2
Characterisation is in agreement with previous reports of this compound.[4]

***N*-benzylformamide (14)**

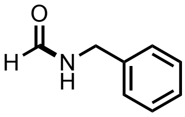

Yield 51%, Mass 0.39g, Off-White Solid.

^{1}H NMR (300 MHz, $CDCl_3$) δ 8.16 – 8.08 (*m,* 1H), 7.34 – 7.13 (*m,* 5H), 6.30 – 6.14 (*m,* 1H), 4.36 (*d, J* = 5.9 Hz, 2H).
^{13}C {^{1}H} NMR (75 MHz, $CDCl_3$) δ 161.2, 137.7, 128.8, 127.8, 127.0, 42.1
Characterisation is in agreement with previous reports of this compound.[4]

***N*-benzylhexanamide (15)**

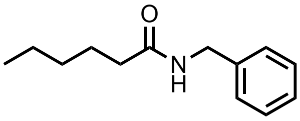

Yield 97%, Mass 1.00g, Yellow Solid.

^{1}H NMR (300 MHz, $CDCl_3$): δ 7.28 – 7.12 (*m,* 5H), 6.08 (*s,* 1H), 4.31 (*d, J* = 5.7 Hz, 2H), 2.10 (*t, J* = 7.6 Hz, 2H), 1.55 (d*q, J* = 9.1, 7.3 Hz, 2H), 1.35 – 1.08 (*m,* 4H), 0.88 – 0.76 (*m,* 3H).
^{13}C {^{1}H} NMR (75 MHz, $CDCl_3$) δ 173.0, 138.4, 128.7, 127.8, 127.5, 44.6, 36.8, 31.5, 25.5, 22.4, 13.9.
Characterisation is in agreement with previous reports of this compound. [8]

N-benzylcyclohexanecarboxamide (16)

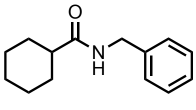

Yield 86%, Mass 0.93g, Brown Solid.

^{1}H NMR (300 MHz, $CDCl_3$): δ 7.32 – 7.13 (*m,* 5H), 5.68 (*s,* 1H), 4.35 (*d, J* = 5.6 Hz, 2H), 2.03 (t*t, J* = 11.7, 3.5 Hz, 1H), 1.88 – 1.54 (*m,* 5H), 1.39 (q*d, J* = 11.9, 3.1 Hz, 2H), 1.29 – 1.06 (*m,* 3H).
^{13}C {^{1}H} NMR (75 MHz, $CDCl_3$): δ 175.9, 138.6, 128.7, 127.7, 127.5, 45.6, 43.4, 29.7, 25.8
Characterisation is in agreement with previous reports of this compound. [9]

N-benzyl-2-phenylacetamide (17)

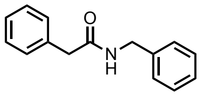

Yield 81%, Mass 0.92g, White Solid.

^{1}**H NMR** (300 MHz, $CDCl_3$): δ 7.38 – 7.15 (*m,* 8H), 7.15 – 7.06 (*m,* 2H), 5.63 (*s,* 1H), 4.34 (*d, J* = 5.8 Hz, 2H), 3.55 (*s,* 2H).
^{13}**C {^{1}H} NMR** (75 MHz, $CDCl_3$): δ 170.9, 138.1, 134.9, 129.5, 129.1, 128.7, 127.5, 127.4, 43.9, 43.6
Characterisation is in agreement with previous reports of this compound. [2]

N-benzyl-3-phenylpropanamide (18)

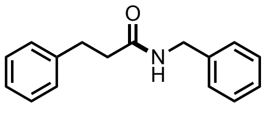

Yield 68%, Mass 0.82g, Brown Solid.

^{1}**H NMR** (300 MHz, $CDCl_3$) δ 7.29 – 7.01 (*m,* 10H), 5.52 (*s,* 1H), 4.33 (*d, J* = 5.7 Hz, 2H), 2.93 (*t, J* = 7.6 Hz, 2H), 2.45 (d*d, J* = 8.3, 7.0 Hz, 2H).
^{13}**C {^{1}H} NMR** (75 MHz, $CDCl_3$) δ 171.99, 140.81, 138.22, 128.66, 128.57, 128.43, 127.73, 127.44, 126.27, 43.56, 38.45, 31.75.
Characterisation is in agreement with previous reports of this compound.[2]

***N*-phenylhexanamide (19)**

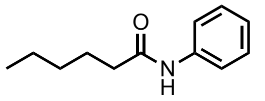

Yield 79%, Mass 0.76g, Dark Brown Solid.

^{1}**H NMR** (300 MHz, $CDCl_3$) δ 7.52 (*d, J* = 7.9 Hz, 2H), 7.37 – 7.28 (*m,* 2H), 7.23 (*s,* 1H), 7.10 (*t, J* = 7.4 Hz, 1H), 2.35 (*t, J* = 7.6 Hz, 2H), 1.74 (*t, J* = 7.5 Hz, 2H), 1.36 (dp, *J* = 7.6, 3.9 Hz, 4H), 0.97 – 0.85 (*m,* 3H).
^{13}**C {^{1}H} NMR** (75 MHz, $CDCl_3$) δ 171.4, 138.0, 129.0, 124.2, 119.8, 37.8, 31.4, 25.3, 22.4, 13.9.
Characterisation is in agreement with previous reports of this compound. [4]

N,2-diphenylacetamide (20)

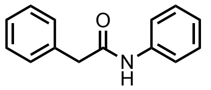

Yield 82%, Mass 0.87g, Light Brown Solid.

^{1}**H NMR** (300 MHz, $CDCl_3$) δ 7.47 – 7.21 (*m,* 9H), 7.17 – 7.01 (*m,* 2H), 3.73 (*s,* 2H).
^{13}**C {^{1}H} NMR** (75 MHz, $CDCl_3$) δ 169.1, 137.6, 134.4, 129.5, 129.3, 128.9, 127.7, 124.5, 119.8, 44.9.
Characterisation is in agreement with previous reports of this compound. [4]

N-cyclohexylhexanamide (21)

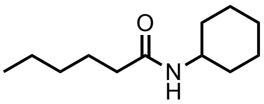

Yield 90%, Mass 0.88g, Dark Brown Solid.

^{1}H NMR (300 MHz, $CDCl_3$) *δ* 5.30 (*s,* 1H), 3.77 (td*t*, *J* = 10.6, 8.0, 4.0 Hz, 1H), 2.19 – 2.07 (*m,* 2H), 1.98 – 1.85 (*m,* 2H), 1.77 – 1.55 (*m,* 5H), 1.46 – 1.25 (*m,* 6H), 1.24 – 1.01 (*m,* 3H), 0.95 – 0.84 (*m,* 3H).
^{13}C {^{1}H} NMR (75 MHz, $CDCl_3$) *δ* 172.2, 48.0, 37.1, 33.3, 31.5, 25.6, 24.9, 22.4, 14.0.
Characterisation is in agreement with previous reports of this compound. [10]

***N*-cyclohexyl-2-phenylacetamide (22)**

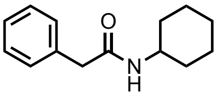

Yield 83%, Mass 0.90g, Brown Solid.

^{1}H NMR (300 MHz, $CDCl_3$) *δ* 7.45 – 7.19 (*m,* 5H), 5.25 (*s,* 1H), 3.87 – 3.68 (*m,* 1H), 3.54 (*s,* 2H), 1.93 – 1.75 (*m,* 2H), 1.59 (t*t*, *J* = 13.1, 4.3 Hz, 3H), 1.45 – 1.22 (*m,* 2H), 1.05 (td*d*, *J* = 23.5, 10.2, 4.6 Hz, 3H).
^{13}C {^{1}H} NMR (75 MHz, $CDCl_3$) *δ* 170.0, 135.2, 129.4, 129.0, 127.3, 48.2, 44.0, 32.9, 25.5, 24.7.
Characterisation is in agreement with previous reports of this compound. [11]

N-cyclohexylcyclohexanecarboxamide (23)

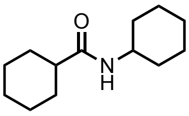

Yield 74%, Mass 0.77g, Brown Solid.

^{1}H NMR (300 MHz, $CDCl_3$) *δ* 3.76 (td*t*, *J* = 10.6, 8.1, 3.9 Hz, 1H), 2.02 (t*t*, *J* = 11.8, 3.4 Hz, 1H), 1.95 – 1.53 (*m,* 10H), 1.51 – 1.01 (*m,* 10H).
^{13}C {^{1}H} NMR (75 MHz, $CDCl_3$) *δ* 175.1, 47.7, 45.7, 33.3, 29.8, 25.8, 25.6, 24.9.
Characterisation is in agreement with previous reports of this compound. [5]

1-morpholinohexan-1-one (24)

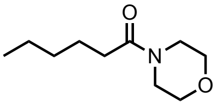

Yield 78%, Mass 0.72g, Orange Liquid.

^{1}H NMR (300 MHz, $CDCl_3$) *δ* 3.73 – 3.38 (*m,* 8H), 2.37 – 2.26 (*m,* 2H), 1.71 – 1.55 (*m,* 2H), 1.33 (d*t*, *J* = 7.3, 3.8 Hz, 4H), 0.99 – 0.84 (*m,* 3H).
^{13}C {^{1}H} NMR (75 MHz, $CDCl_3$) *δ* 171.9, 66.8, 46.1, 33.1, 31.6, 25.0, 22.5, 14.0.
Characterisation is in agreement with previous reports of this compound. [5]

1-morpholino-2-phenylethan-1-one (25)

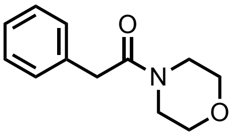

Yield 81%, Mass 0.83g, Dark Brown Solid.

^{1}H NMR (300 MHz, $CDCl_3$) δ 7.38 – 7.30 (*m,* 2H), 7.30 – 7.22 (*m,* 3H), 3.74 (*s,* 2H), 3.65 (*s,* 4H), 3.53 – 3.41 (*m,* 4H).
^{13}C {^{1}H} NMR (75 MHz, $CDCl_3$) δ 169.6, 134.8, 128.8, 128.5, 126.9, 66.8, 66.5, 46.5, 42.1, 40.8
Characterisation is in agreement with previous reports of this compound. [12]

N-benzyl-2-hydroxypropanamide (26)

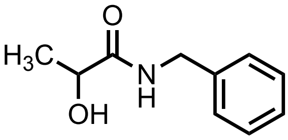

Yield 68%, Mass 0.61g, White Solid.

^{1}H NMR (300 MHz, $CDCl_3$) δ 7.39 – 7.23 (*m,* 5H), 6.81 (*s,* 1H), 4.46 (*d, J* = 5.9 Hz, 2H), 4.29 (d*d, J* = 6.9, 3.1 Hz, 1H), 2.63 (*s,* 1H), 1.47 (*d, J* = 6.8 Hz, 3H).
^{13}C {^{1}H} NMR (75 MHz, $CDCl_3$) δ 174.1, 138.0, 128.8, 127.8, 127.6, 68.5, 43.2, 21.4.
Characterisation is in agreement with previous reports of this compound [13]

N-benzyl-3,3,3-trifluoropropanamide (27)

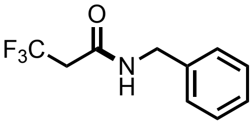

Yield 69%, Mass 0.75g, Brown Solid.

^{1}H NMR (300 MHz, $CDCl_3$) δ 7.34 – 7.13 (*m,* 5H), 6.09 (*s,* 1H), 4.38 (d*d, J* = 13.2, 5.7 Hz, 2H), 3.03 (*q, J* = 10.6 Hz, 2H).
^{13}C {^{1}H} NMR (75 MHz, $CDCl_3$) δ 162.6, 137.3, 128.8, 127.8, 125.9, 44.0, 41.4.
^{19}F NMR (282 MHz, $CDCl_3$) δ -62.91 (*t, J* = 10.6 Hz).
Characterisation is in agreement with previous reports of this compound [14]

N-phenyl-3,3,3-trifluoropropanamide (28)

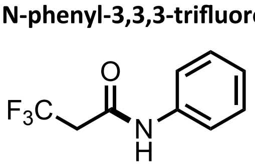

Yield 59%, Mass 0.60g, Brown Solid.

^{1}H NMR (300 MHz, $CDCl_3$) δ 7.81 (*s,* 1H), 7.54 – 7.44 (*m,* 2H), 7.32 (d*d, J* = 8.7, 7.1 Hz, 2H), 7.21 – 7.10 (*m,* 1H), 3.22 (*q, J* = 10.4 Hz, 2H).

^{13}C {^{1}H} NMR (75 MHz, $CDCl_3$) δ 161.0, 136.9, 129.1, 125.3, 120.5, 42.5 (*q, J* = 29.2 Hz).
^{19}F NMR (282 MHz, $CDCl_3$) δ -62.84 (*t, J* = 10.4 Hz).
Characterisation is in agreement with previous reports of this compound [14]

***N*-(4-chlorobenzyl)hexanamide (29)**

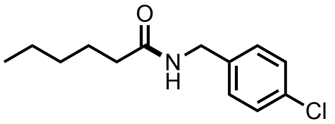

Yield 93%, Mass 1.11g, Yellow Solid.

^{1}H NMR (300 MHz, $CDCl_3$) δ 7.25 – 7.18 (*m,* 2H), 7.12 (*d, J* = 8.5 Hz, 2H), 5.92 (*s,* 1H), 4.31 (*d, J* = 5.8 Hz, 2H), 2.12 (*t, J* = 7.6 Hz, 2H), 1.64 – 1.50 (*m,* 2H), 1.32 – 1.17 (*m,* 4H), 0.89 – 0.74 (*m,* 3H).
^{13}C {^{1}H} NMR (75 MHz, $CDCl_3$) δ 173.2, 137.1, 133.2, 129.1, 128.8, 42.8, 36.7, 31.5, 25.4, 22.4, 14.0
Characterisation is in agreement with previous reports of this compound [15]

----

ethyl (*tert*-butoxycarbonyl)glycylphenylalaninate (30)

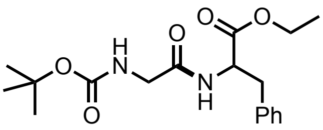

Yield 57%, Mass 0.99g, Yellow Solid.

^{1}H NMR (300 MHz, $CDCl_3$) δ 7.25 – 7.12 (*m,* 3H), 7.07 – 7.01 (*m,* 2H), 6.55 (*d, J* = 7.9 Hz, 1H), 5.11 (*s,* 1H), 4.78 (d*t, J* = 7.9, 5.9 Hz, 1H), 4.08 (*q, J* = 7.1 Hz, 2H), 3.71 (d*d, J* = 10.7, 5.5 Hz, 2H), 3.04 (d*d, J* = 5.9, 1.4 Hz, 2H), 1.37 (*s,* 9H), 1.15 (*t, J* = 7.2 Hz, 3H).
^{13}C {^{1}H} NMR (75 MHz, $CDCl_3$) δ 171.4, 169.2, 156.0, 135.9, 129.4, 128.7, 127.2, 80.3, 61.7, 53.2, 44.3, 38.1, 28.4, 14.2
Characterisation is in agreement with previous reports of this compound [4]

***N*-(quinolin-8-yl)hexanamide (31)**

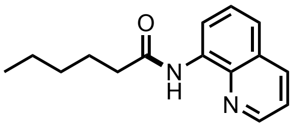

Yield 8%, Mass 0.05g, Dark Brown Liquid.

^{1}H NMR (400 MHz, $CDCl_3$) δ 9.81 (*s,* 1H), 8.90 – 8.71 (*m,* 2H), 8.21 – 8.08 (*m,* 1H), 7.60 – 7.40 (*m,* 3H), 2.56 (*t, J* = 7.6 Hz, 2H), 1.83 (*t, J* = 7.4 Hz, 2H), 1.40 (d*t, J* = 8.9, 5.0 Hz, 4H), 1.03 – 0.87 (*m,* 3H).
^{13}C {^{1}H} NMR (101 MHz, $CDCl_3$) δ 172.1, 148.2, 138.5, 136.5, 134.7, 128.1, 127.6, 121.7, 121.4, 116.5, 38.3, 31.6, 25.5, 22.6, 14.1.

Characterisation is in agreement with previous reports of this compound [16]

***N-(5-hydroxypentyl)hexanamide* (32)**

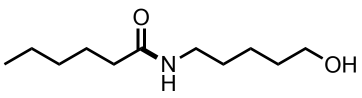

Yield 46%, Mass 0.46g, White Solid.

^{1}H NMR (300 MHz, $CDCl_3$) *δ* 3.58 (*t, J* = 6.3 Hz, 2H), 3.19 (t*d, J* = 6.9, 5.8 Hz, 2H), 2.16 – 2.02 (*m,* 3H), 1.70 (*s,* 1H), 1.63 – 1.10 (*m,* 12H), 0.91 – 0.74 (*m,* 3H).
^{13}C {^{1}H} NMR (75 MHz, $CDCl_3$) *δ* 173.5, 62.3, 39.3, 36.8, 32.2, 31.5, 29.4, 25.5, 23.1, 22.4, 13.9
No reported characterisation is available for this compound.

Benzyl 2-hydroxy-2-phenylacetate (33)

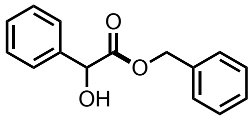

Yield 92%, Mass 1.11g, White Solid.

^{1}H NMR (300 MHz, $CDCl_3$) *δ* 7.39 – 7.07 (*m,* 10H), 5.16 (*d, J* = 12.2 Hz, 2H), 5.06 (*d, J* = 12.3 Hz, 1H).
^{13}C {^{1}H} NMR (75 MHz, $CDCl_3$) *δ* 173.5, 138.2, 135.0, 128.6, 128.6, 128.5, 128.5, 128.0, 126.6, 73.0, 67.7
Characterisation is in agreement with previous reports of this compound. [17]

Butyl 2-hydroxy-2-phenylacetate (34)

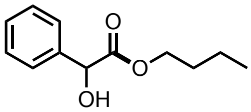

Yield 83%, Mass 0.87g, Colorless Liquid.

^{1}H NMR (300 MHz, $CDCl_3$) *δ* 7.43 – 7.22 (*m,* 5H), 5.09 (*d, J* = 5.5 Hz, 1H), 4.20 – 4.01 (*m,* 2H), 3.40 (*d, J* = 5.8 Hz, 1H), 1.59 – 1.42 (*m,* 2H), 1.18 (d*q, J* = 14.5, 7.3 Hz, 2H), 0.78 (*t, J* = 7.4 Hz, 3H).
^{13}C {^{1}H} NMR (75 MHz, $CDCl_3$) *δ* 173.8, 138.5, 128.5, 128.4, 126.5, 72.9, 66.1, 30.4, 18.9, 13.5
Characterisation is in agreement with previous reports of this compound. [18]

Butyl 4-nitrobenzoate (35)

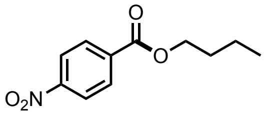

Yield 41%, Mass 0.45g, Yellow Solid

^{1}H NMR (300 MHz, $CDCl_3$) *δ* 8.32 – 8.26 (*m,* 2H), 8.24 – 8.18 (*m,* 2H), 4.38 (*t, J* = 6.6 Hz, 2H), 1.85 – 1.72 (*m,* 2H), 1.57 – 1.41 (*m,* 2H), 0.99 (*t, J* = 7.4 Hz, 3H).
^{13}C {^{1}H} NMR (75 MHz, $CDCl_3$) *δ* 164.8, 150.5, 135.9, 130.7, 123.5, 65.8, 30.6, 19.2, 13.8
No reported characterisation is available for this compound.

Benzyl hexanoate (36)

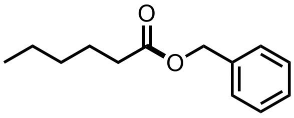

Yield 78%, Mass 0.80g, Dark Brown Liquid.

^{1}**H NMR** (300 MHz, $CDCl_3$) δ 7.46 – 7.29 (*m,* 5H), 5.12 (*s,* 2H), 2.36 (*t, J* = 7.5 Hz, 2H), 1.75 – 1.58 (*m,* 2H), 1.31 (t*t, J* = 7.6, 3.1 Hz, 4H), 0.89 (h, *J* = 2.8 Hz, 3H).
^{13}**C {^{1}H} NMR** (75 MHz, $CDCl_3$) δ 173.7, 136.2, 128.6, 128.2, 66.1, 34.3, 31.3, 24.7, 22.3, 13.9
Characterisation is in agreement with previous reports of this compound.[19]

Butyl hexanoate (37)

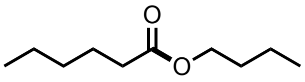

Yield 53%, Mass 0.46g, Colorless Liquid.

^{1}**H NMR** (300 MHz, $CDCl_3$) δ 4.00 (*t, J* = 6.7 Hz, 2H), 2.22 (*t, J* = 7.5 Hz, 2H), 1.65 – 1.48 (*m,* 4H), 1.38 – 1.18 (*m,* 6H), 0.84 (d*t, J* = 11.0, 7.1 Hz, 6H).
^{13}**C {^{1}H} NMR** (75 MHz, $CDCl_3$) δ 174.0, 64.1, 34.4, 31.3, 30.7, 24.7, 22.3, 19.2, 13.9, 13.7
Characterisation is in agreement with previous reports of this compound.[20]

Butyl heptanoate (38)

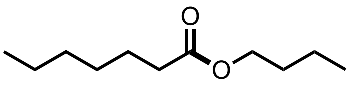

Yield 59%, Mass 0.54g, Colorless Liquid.

^{1}**H NMR** (300 MHz, $CDCl_3$) δ 4.07 (*t, J* = 6.7 Hz, 2H), 2.29 (*t, J* = 7.5 Hz, 2H), 1.70 – 1.53 (*m,* 4H), 1.47 – 1.23 (*m,* 8H), 0.91 (d*t, J* = 14.4, 7.1 Hz, 6H).
^{13}**C {^{1}H} NMR** (75 MHz, $CDCl_3$) δ 174.0, 64.1, 34.4, 31.5, 30.7, 28.8, 25.0, 22.5, 19.2, 14.0, 13.7
Characterisation is in agreement with previous reports of this compound.[21]

Butyl nonanoate (39)

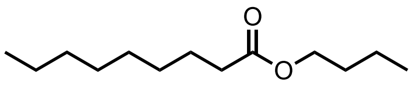

Yield 78%, Mass 0.84g, Colorless Liquid.

^{1}**H NMR** (300 MHz, $CDCl_3$) δ 4.00 (*t, J* = 6.6 Hz, 2H), 2.22 (*t, J* = 7.5 Hz, 2H), 1.62 – 1.47 (*m,* 4H), 1.39 – 1.14 (*m,* 12H), 0.94 – 0.75 (*m,* 6H).
^{13}**C {^{1}H} NMR** (75 MHz, $CDCl_3$) δ 174.0, 64.1, 34.4, 31.8, 30.7, 29.2, 29.2, 29.1, 25.0, 22.6, 19.2, 14.1, 13.7
Characterisation is in agreement with previous reports of this compound.[22]

Butyl decanoate (40)

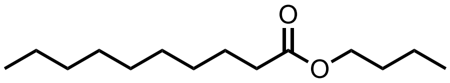

Yield 65%, Mass 0.74g, Colorless Liquid.

^{1}H NMR (300 MHz, $CDCl_3$) δ 4.07 (*t, J* = 6.7 Hz, 2H), 2.29 (*t, J* = 7.5 Hz, 2H), 1.61 (t*t, J* = 8.7, 5.3 Hz, 4H), 1.46 – 1.20 (*m,* 14H), 0.90 (d*t, J* = 16.2, 7.1 Hz, 6H).
^{13}C {^{1}H} NMR (75 MHz, $CDCl_3$) δ 174.0, 64.1, 34.4, 31.9, 30.7, 29.4, 29.3, 29.2, 25.0, 22.7, 19.2, 14.1, 13.7
Characterisation is in agreement with previous reports of this compound.[23]

References:

1. J. He, Y. Li, X. Xue, H. Ru, X. Huang and H. Yang, *RSC Adv.*, 2017, **7**, 14053-14059.
2. P. V. Ramachandran and H. J. Hamann, *Org. Lett.*, 2021, **23**, 2938-2942.
3. P. V. Ramachandran, A. Singh, H. Walker and H. J. Hamann, *Molecules,* 2024, **29**, 268.
4. A. A. Alawaed and P. V. Ramachandran, *Org. Biomol. Chem.*, 2024, **22**, 1915-1919.
5. A. G. Singh and P. V. Ramachandran, *Journal of Fluorine Chemistry*, 2024, **278**, 110328.
6. P. N. Muskawar, K. Thenmozhi and P. R. Bhaga*t, Applied Catalysis A: General*, 2015, **493**, 158-167.
7. P.-L. Lagueux-Tremblay, A. Fabrikant and B. A. Arndtsen, *ACS Catal.*, 2018, **8**, 5350-5354.
8. S. E. Ki*m,* H. Hah*m,* S. Ki*m,* W. Jang, B. Jeon, Y. Kim and M. Ki*m, Asian J. Org. Chem.*, 2016, **5**, 222-231.
9. H. Lundberg, F. Tinnis and H. Adolfsson, *Synlett,* 2012, **23**, 2201-2204.
10. C. Li, M. Wang, X. Lu, L. Zhang, J. Jiang and L. Zhang, *ACS Sustainable Chemistry & Engineering*, 2020, **8**, 4353-4361.
11. W. Yu, S. Yang, F. Xiong, T. Fan, Y. Feng, Y. Huang, J. Fu and T. Wang, *Org. Biomol. Chem.*, 2018, **16**, 3099-3103.
12. H. Lundberg, F. Tinnis and H. Adolfsson, *Chem. Eur. J.*, 2012, **18**, 3822-3826.
13. M. Trincado, K. Kühlein and H. Grützmacher, *Chem. Eur. J.*, 2011, **17**, 11905-11913.
14. X. Du, W.-M. Zhang, X.-G. Zhang and H.-Y. Tu, *Adv. Synth. Catal.*, 2022, **364**, 2546-2550.
15. S. C. Ghosh, C. C. Li, H. C. Zeng, J. S. Y. Ngia*m,* A. M. Seayad and A. Chen, *Adv. Synth. Catal.*, 2014, **356**, 475-484.
16. Y. Huang, X. Lv, H.-R. Tong, W. He, Z. Bai, H. Wang, G. He and G. Chen, *Org. Lett.*, 2024, **26**, 94-99.
17. C.-T. Chen and Y. S. Muno*t, J. Org. Chem.*, 2005, **70**, 8625-8627.
18. H. Prydderch, A. Haiβ, M. Spulak, B. Quilty, K. Kümmerer, A. Heise and N. Gathergoo*d, RSC Adv.*, 2017, **7**, 2115-2126.
19. A. Chan and K. A. Scheid*t, Org. Lett.*, 2005, **7**, 905-908.
20. Z. Huang, J. E. Reilly and R. N. Buckle, *Synlett,* 2007, **2007**, 1026-1030.
21. G. Pandey, S. Koley, R. Talukdar and P. K. Sahani, *Org. Lett.*, 2018, **20**, 5861-5865.
22. J.-C. Hsieh, Y.-H. Chu, K. Muralirajan and C.-H. Cheng, *Chem. Commun.*, 2017, **53**, 11584-11587.
23. H. Baek, M. Minakawa, Y. M. A. Yamada, J. W. Han and Y. Uozumi, *Scientific Reports,* 2016, **6**, 25925.

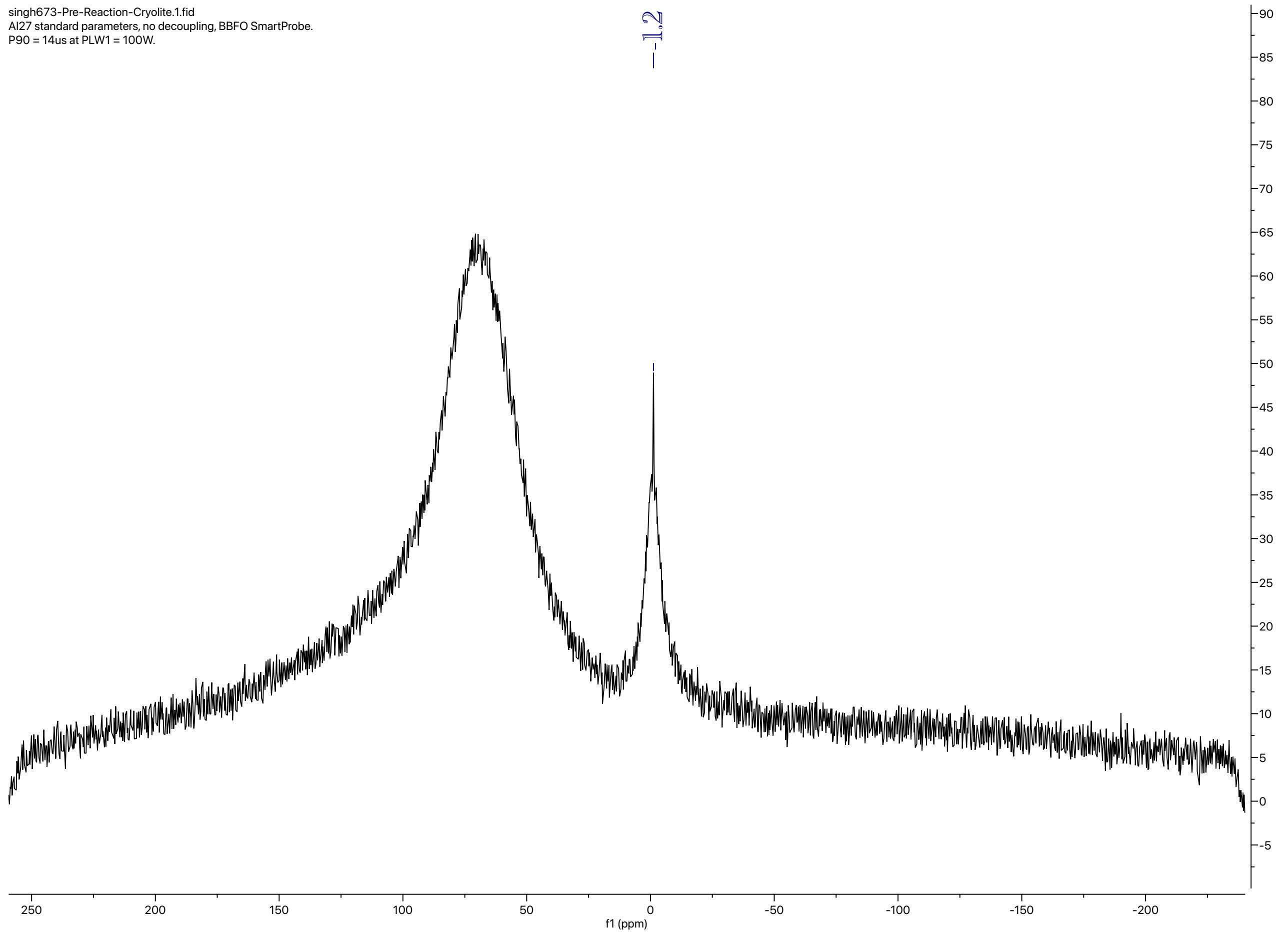

^{27}Al NMR (104 MHz, D_2O) Pre-Reaction Cryolite

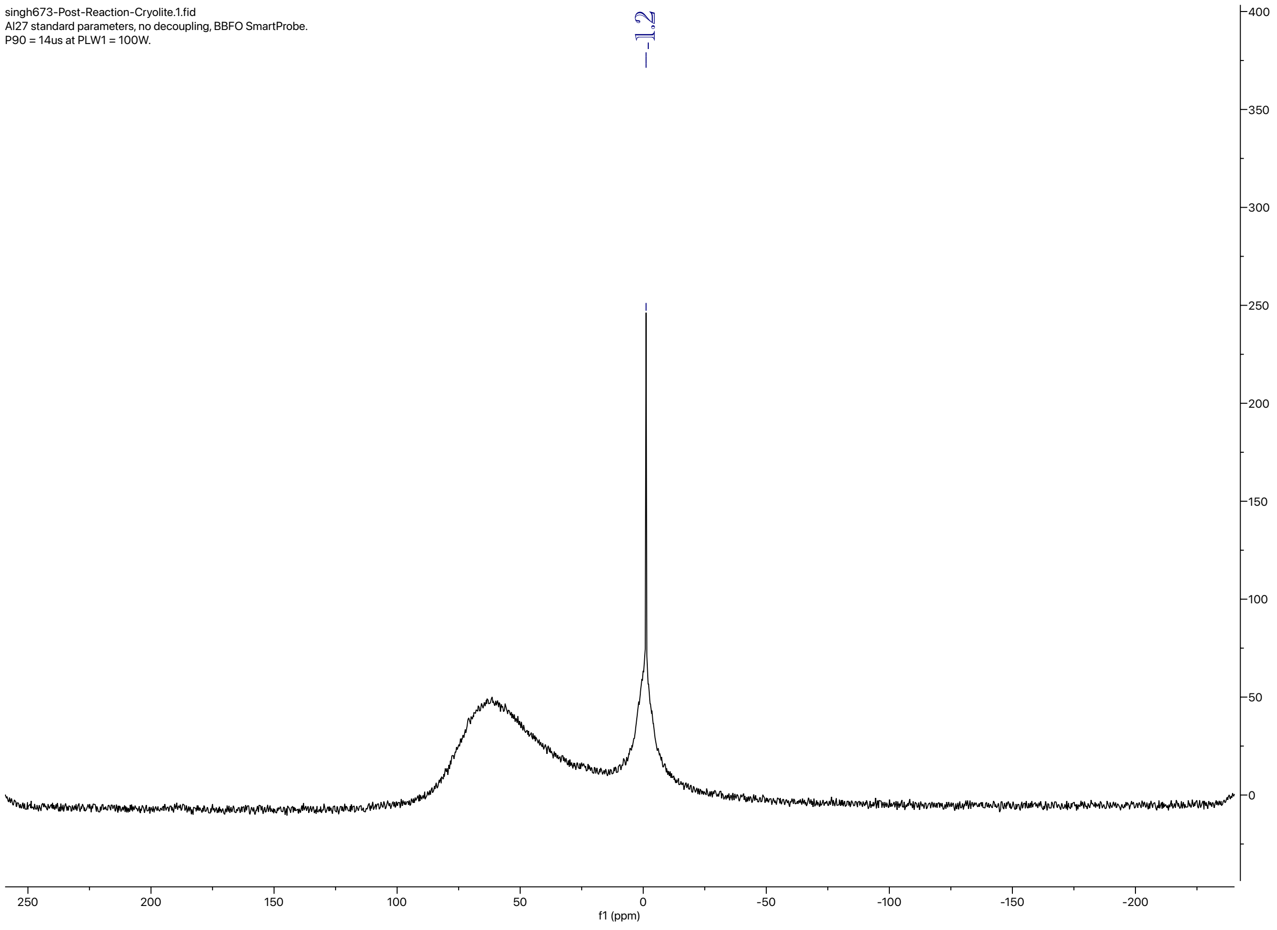

^{27}Al NMR (104 MHz, D_2O) Post-Reaction Cryolite

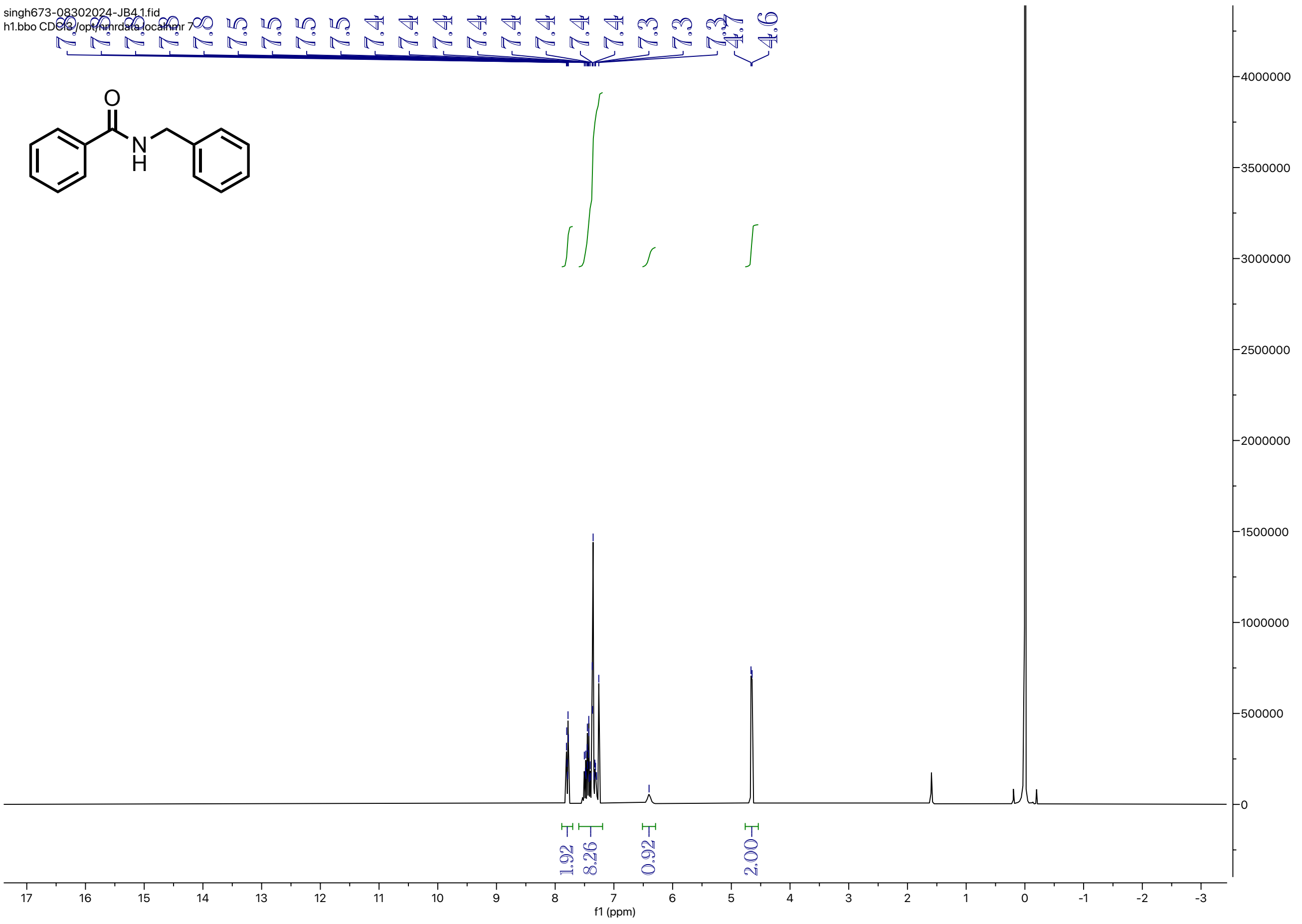

^{1}H NMR (300 MHz, Chloroform-*d*) *N*-benzylbenzamide (**1**)

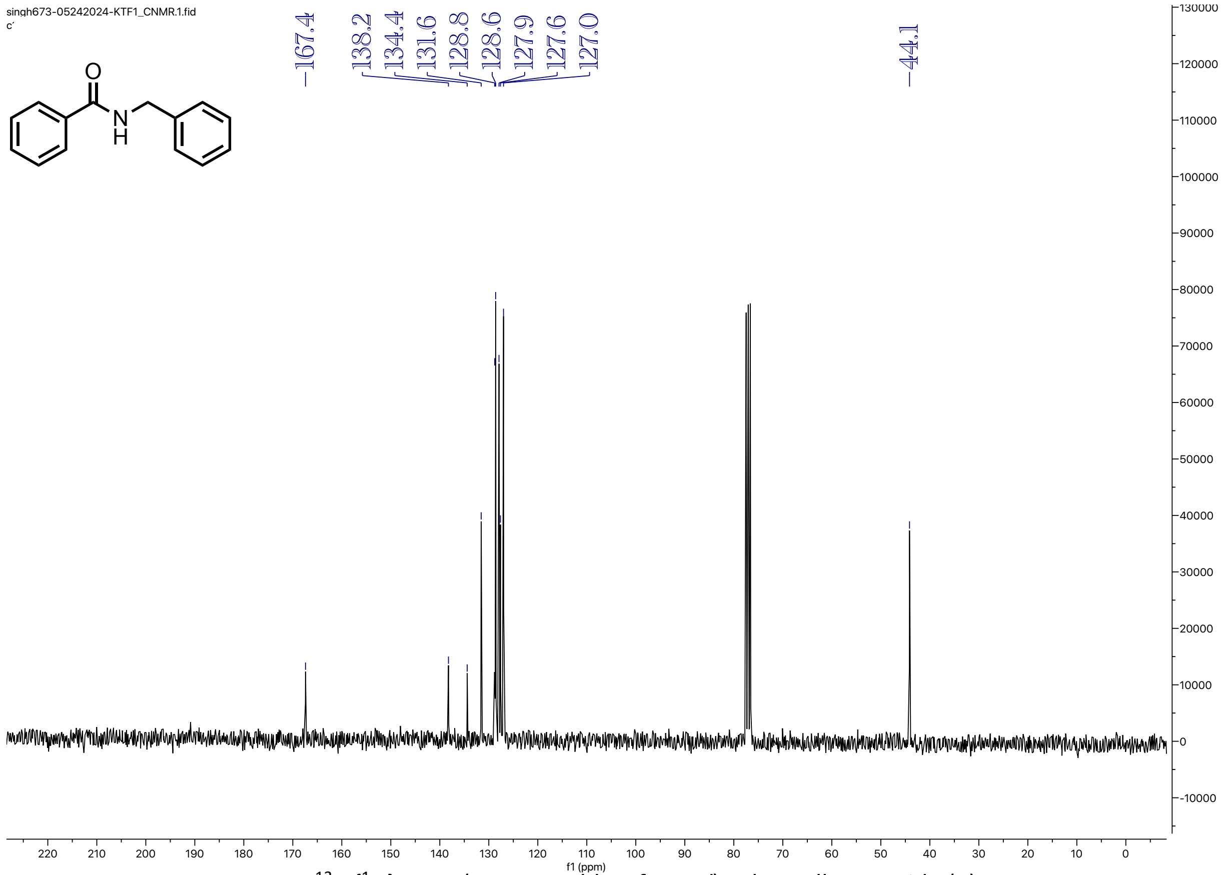

^{13}C {^{1}H} NMR (75 MHz, Chloroform-*d*) *N*-benzylbenzamide (**1**)

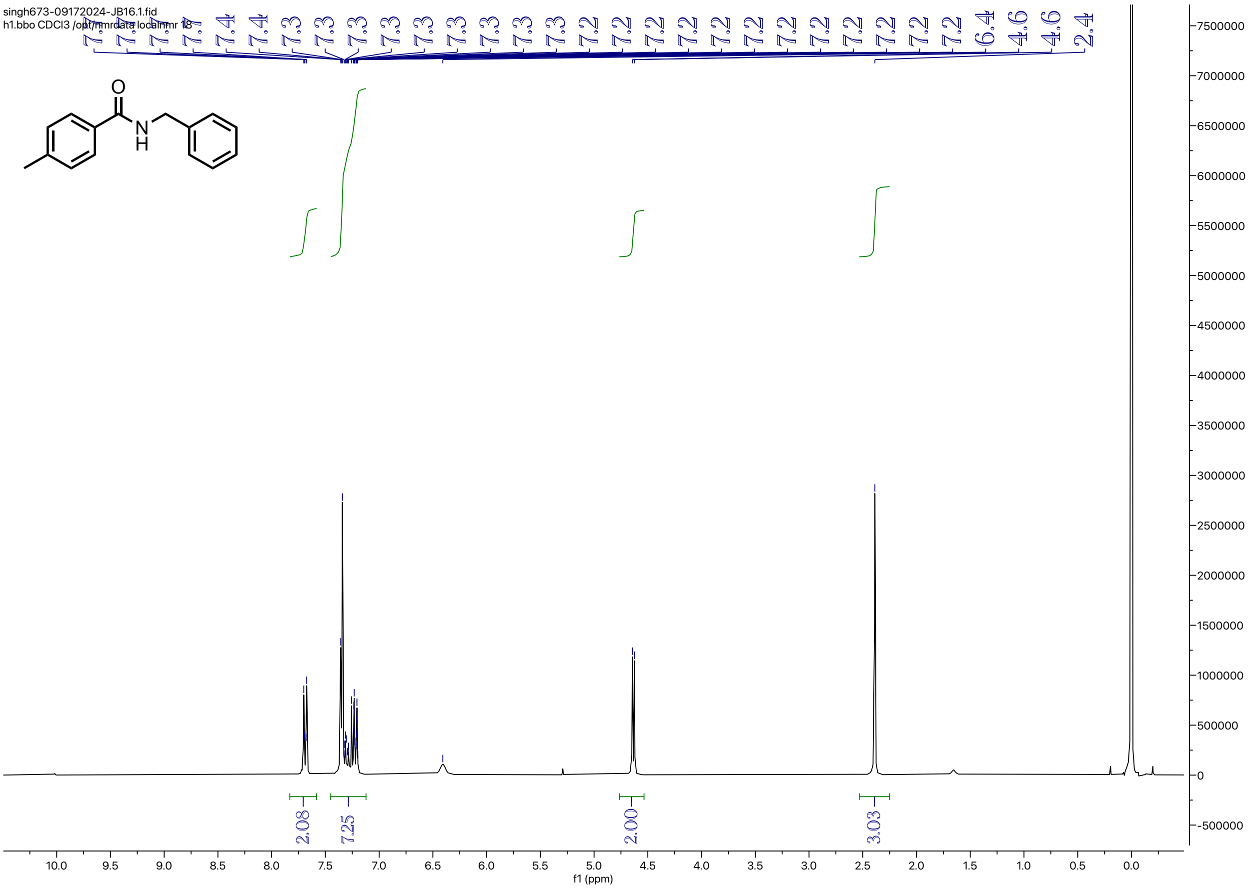

^{1}H NMR (300 MHz, Chloroform-*d*) *N*-benzyl-4-methylbemzamide (**2**)

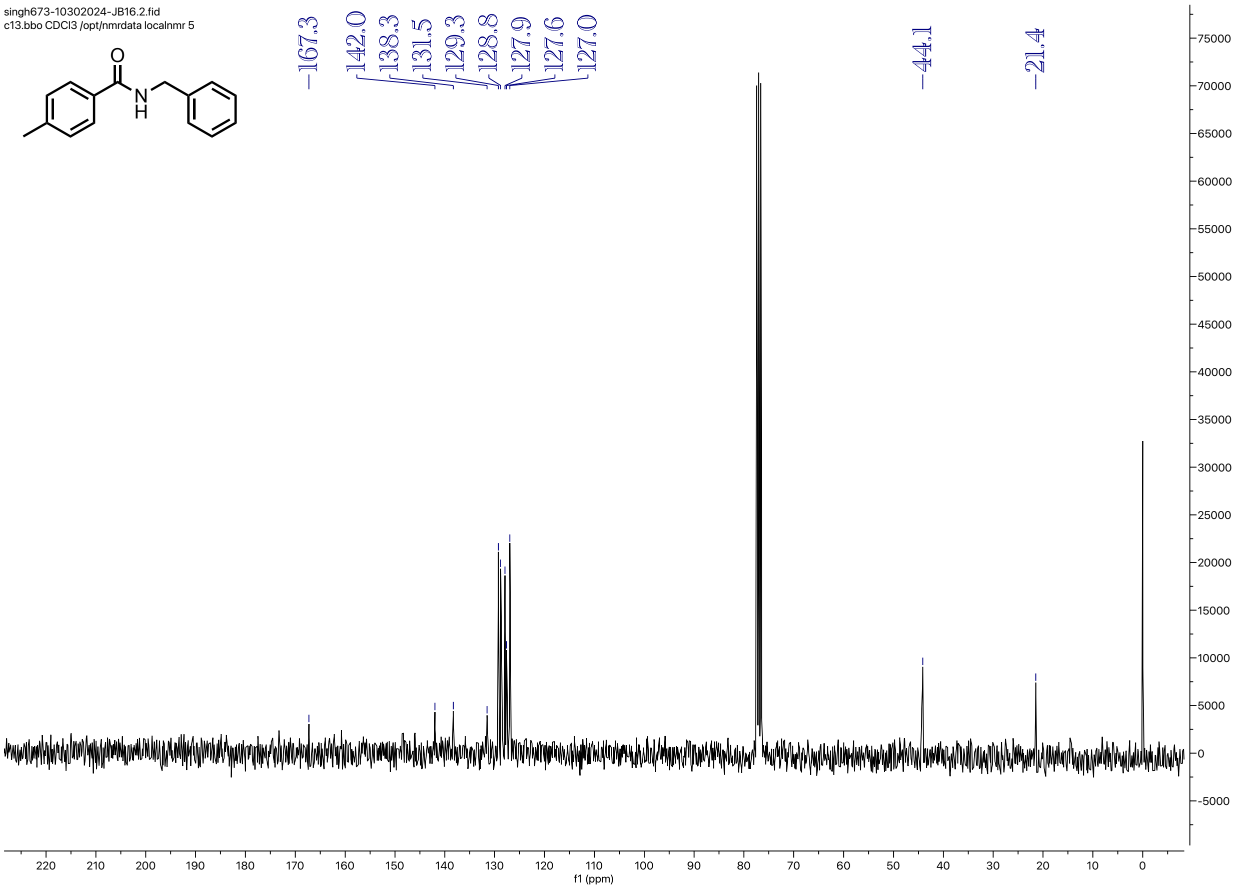

^{13}C {^{1}H} NMR (75 MHz, Chloroform-*d*) *N*-benzyl-4-methylbemzamide (**2**)

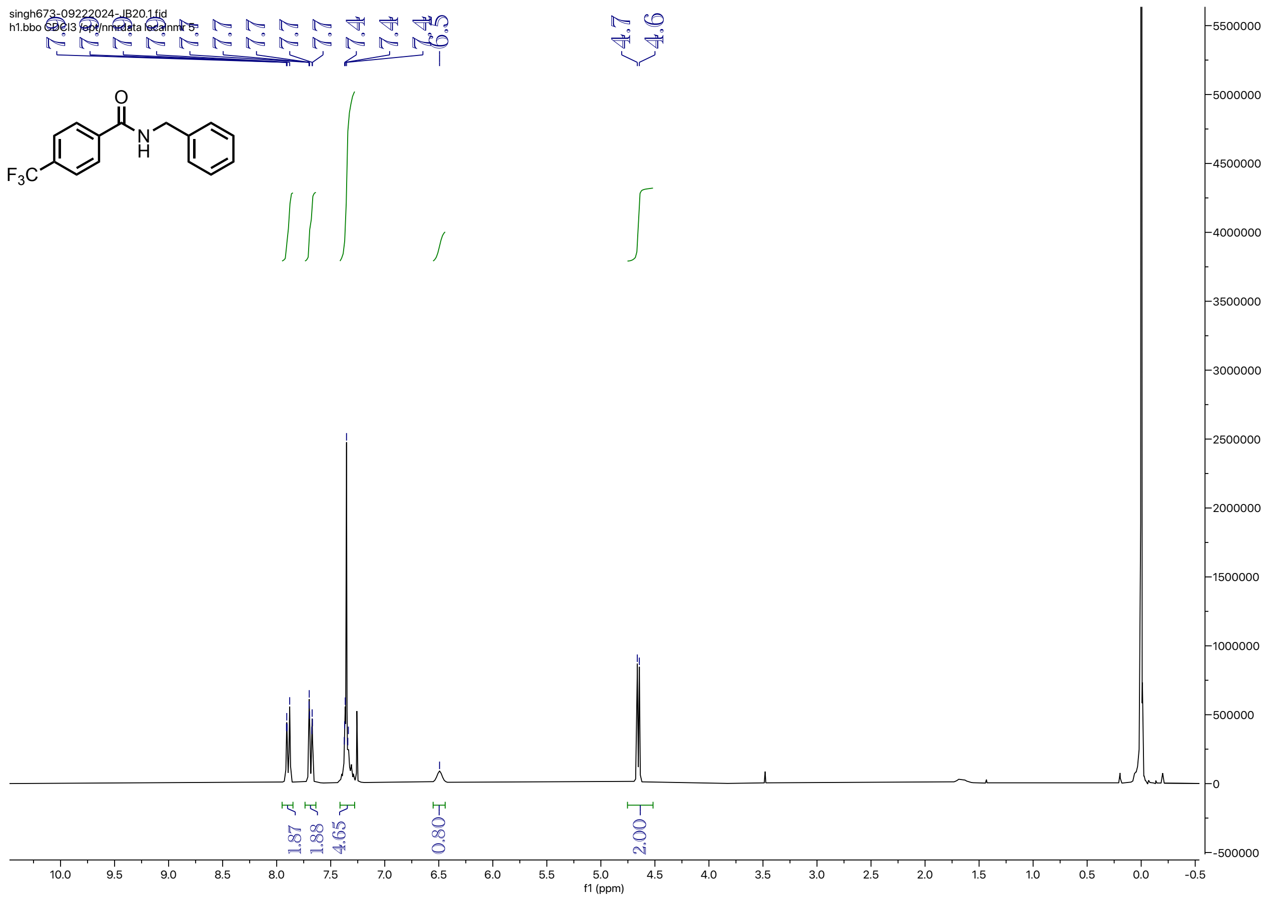

^{1}H NMR (300 MHz, Chloroform-*d*) *N*-benzyl-4-(trifluoromethyl)benzamide (**3**)

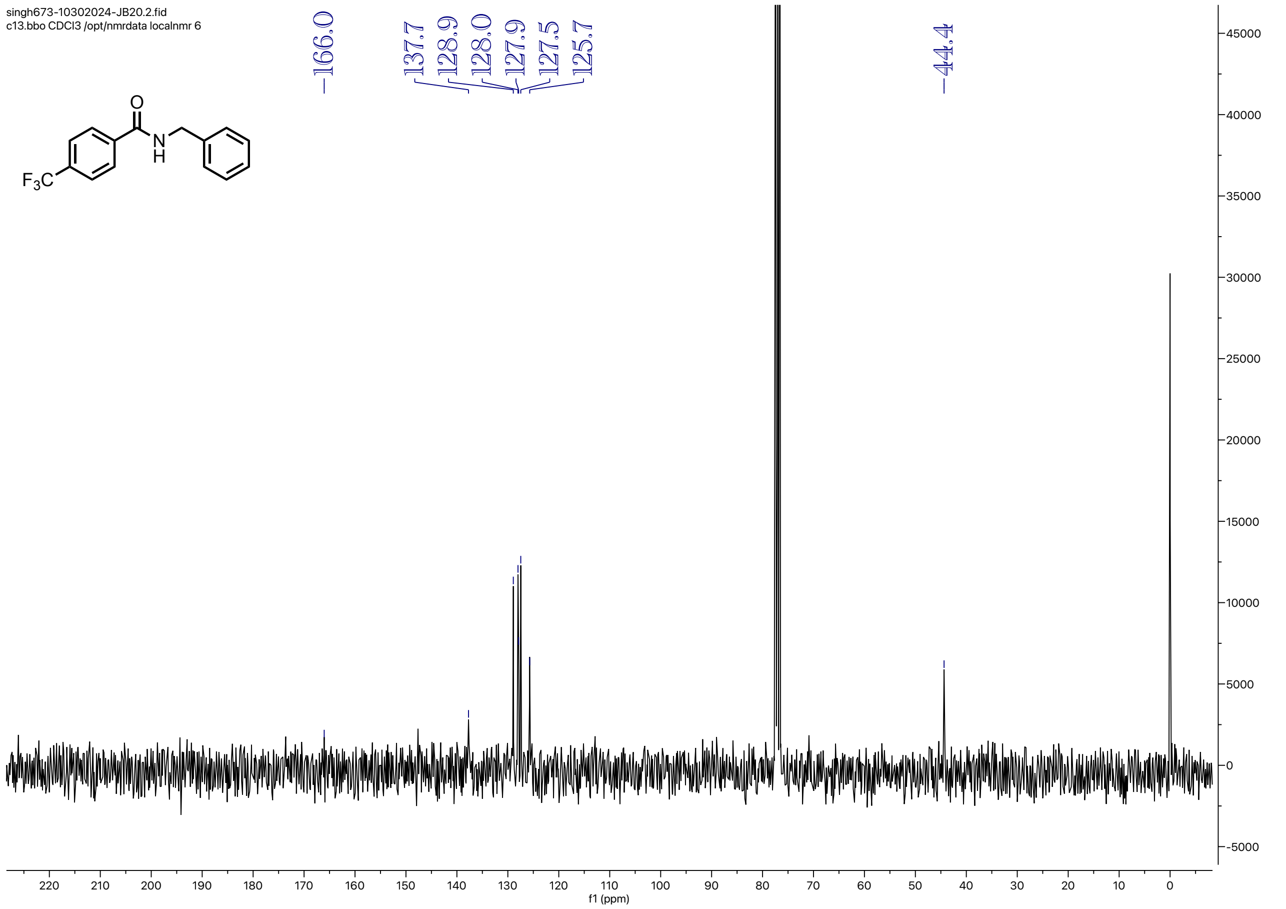

^{13}C {^{1}H} NMR (75 MHz, Chloroform-*d*) *N*-benzyl-4-(trifluoromethyl)benzamide (**3**)

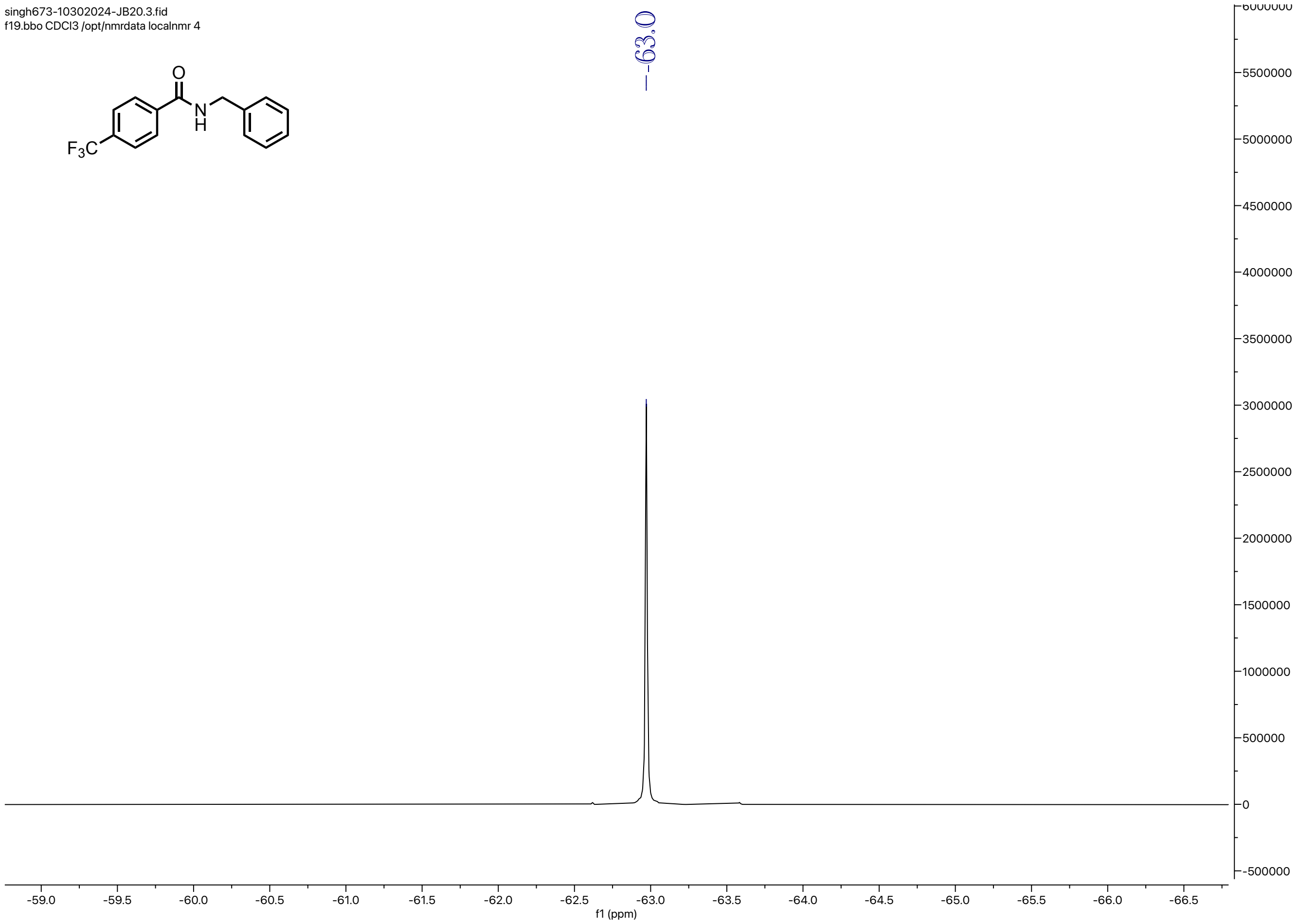

^{19}F NMR (282 MHz, Chloroform-*d*) *N*-benzyl-4-(trifluoromethyl)benzamide (**3**)

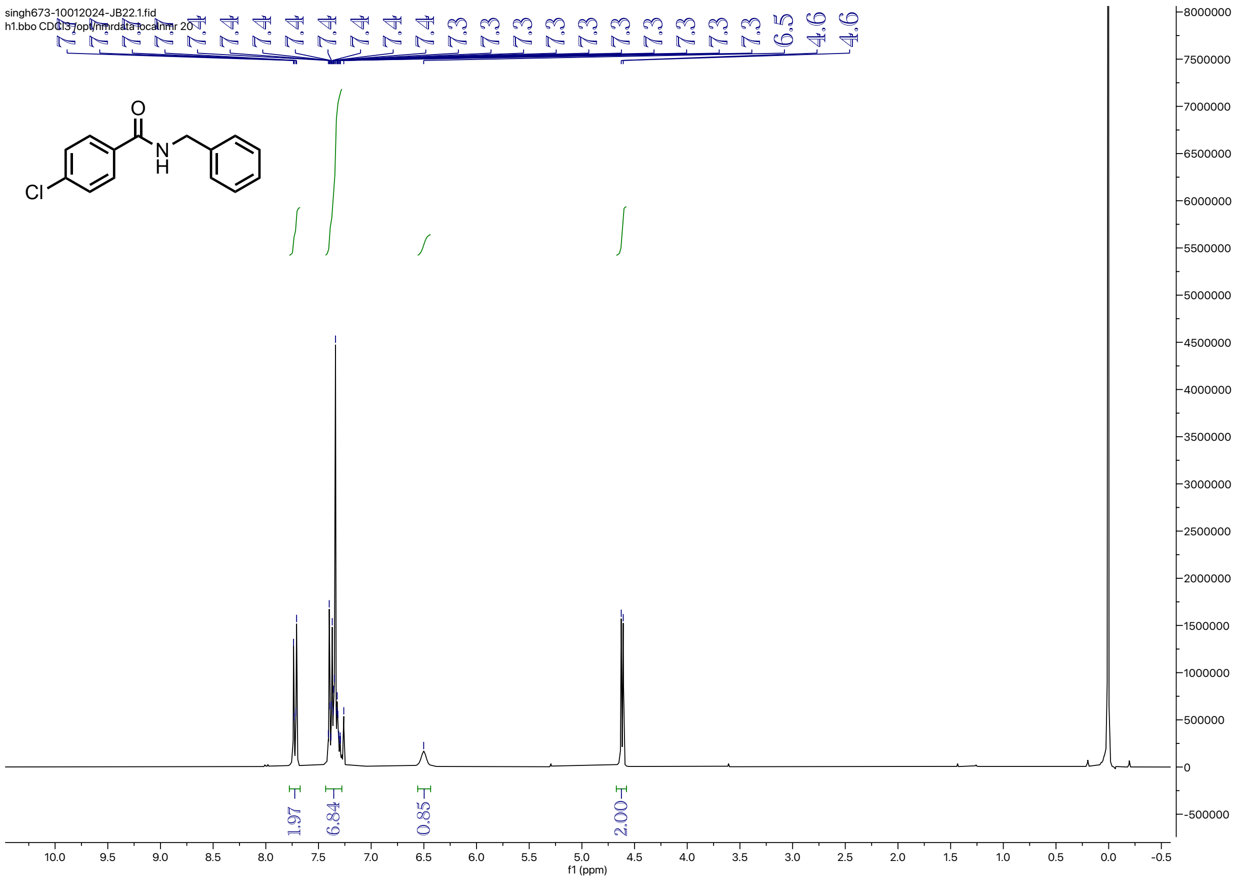

^{1}H NMR (300 MHz, Chloroform-*d*) *N*-benzyl-4-chlorobenzamide (**4**)

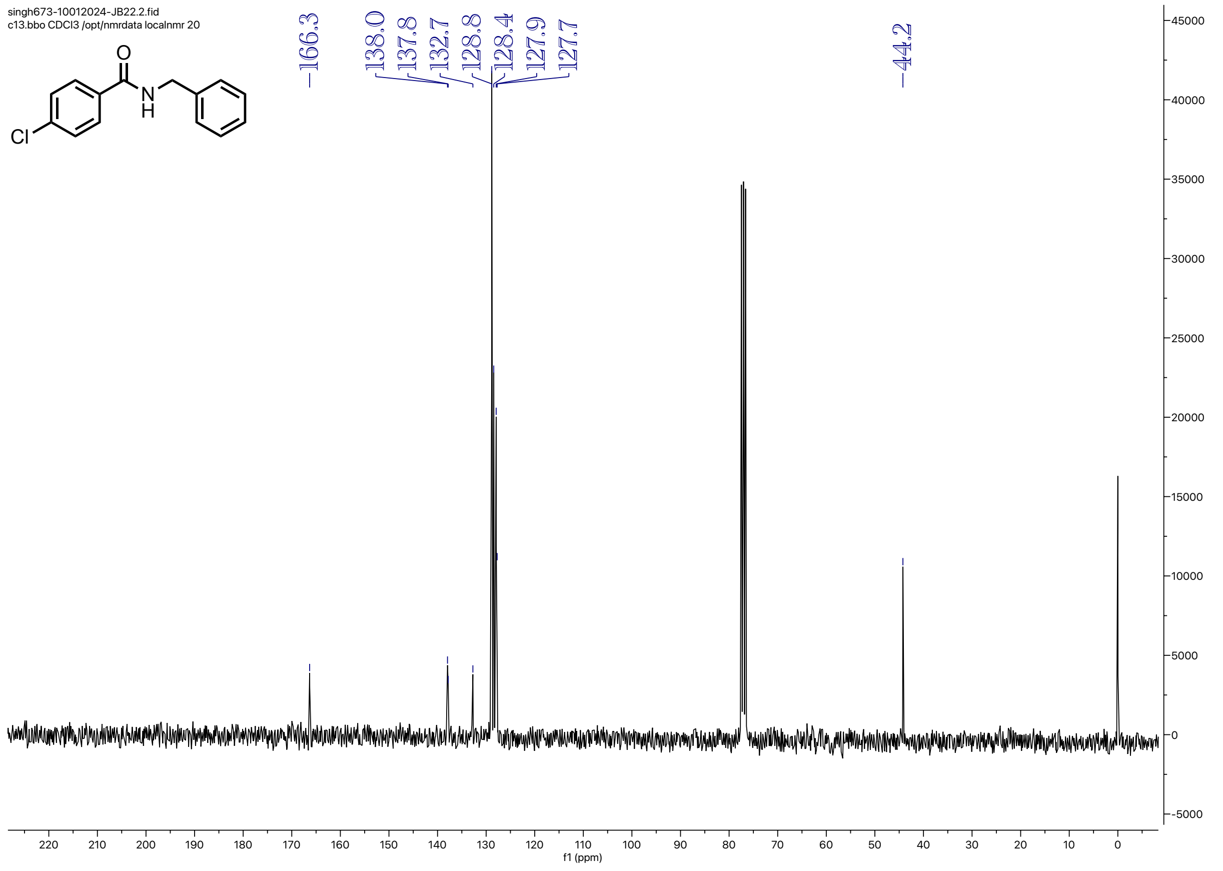

^{13}C {^{1}H} NMR (75 MHz, Chloroform-*d*) *N*-benzyl-4-chlorobenzamide (**4**)

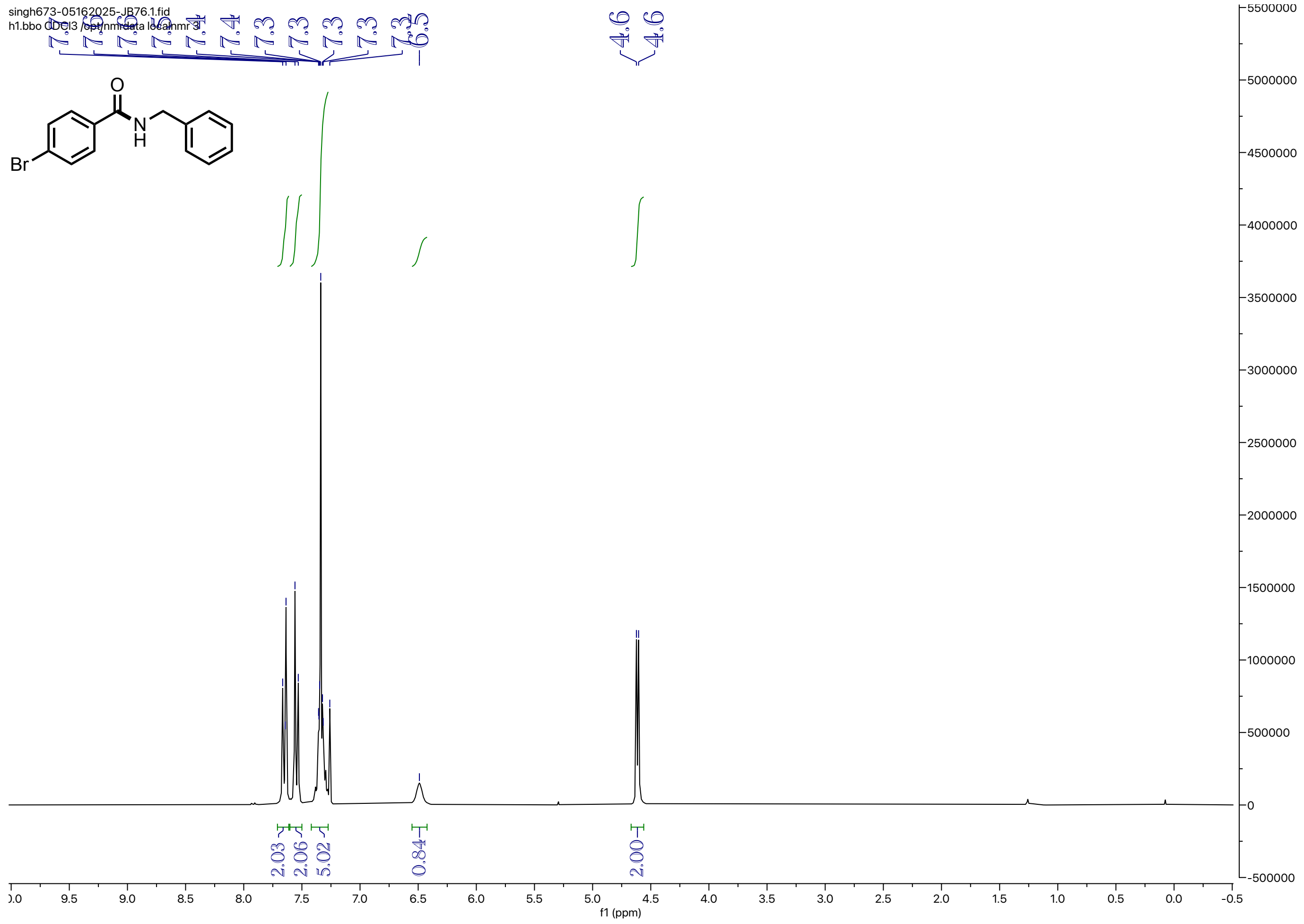

^{1}H NMR (300 MHz, Chloroform-*d*) *N*-benzyl-4-bromobenzamide (**5**)

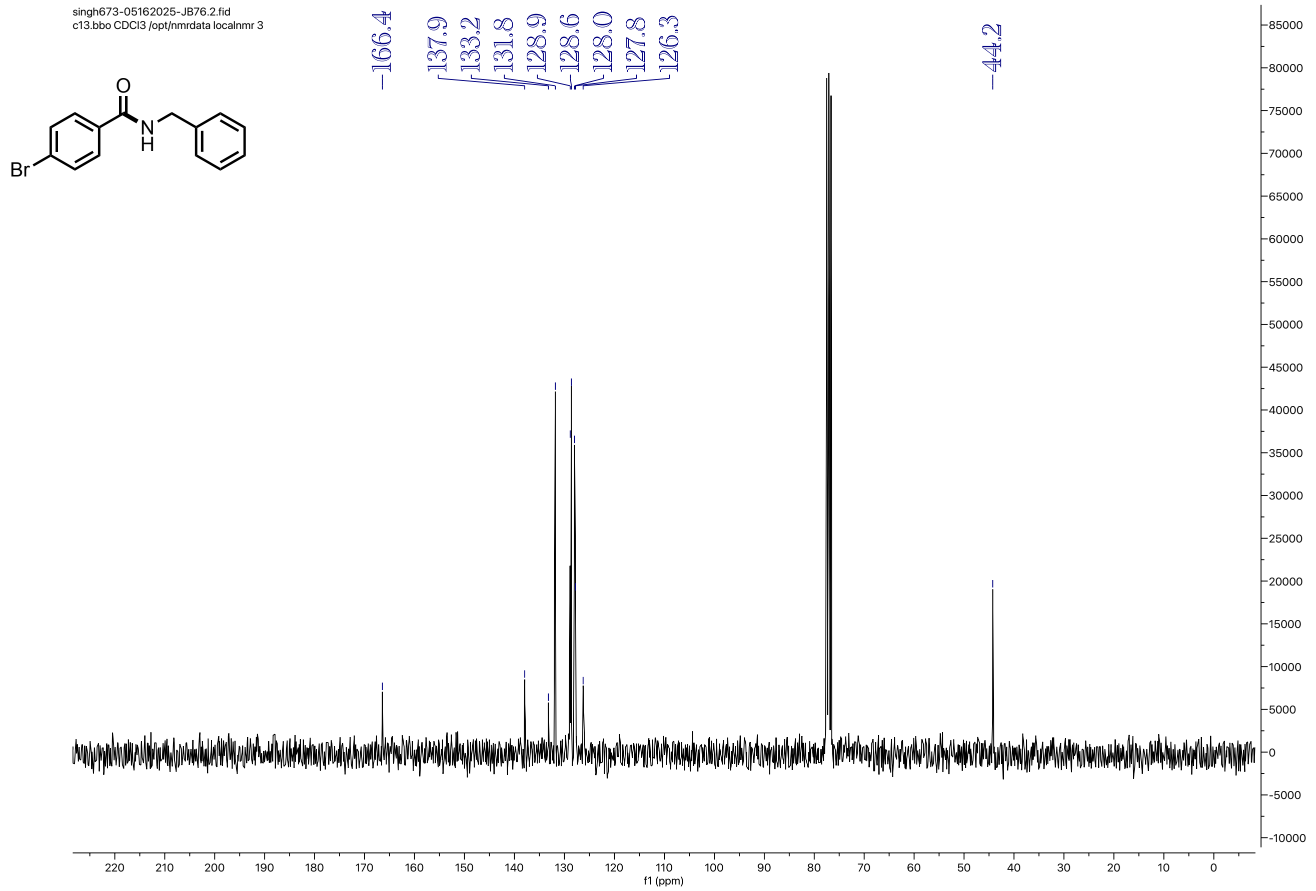

^{13}C {^{1}H} NMR (75 MHz, Chloroform-*d*) *N*-benzyl-4-bromobenzamide (**5**)

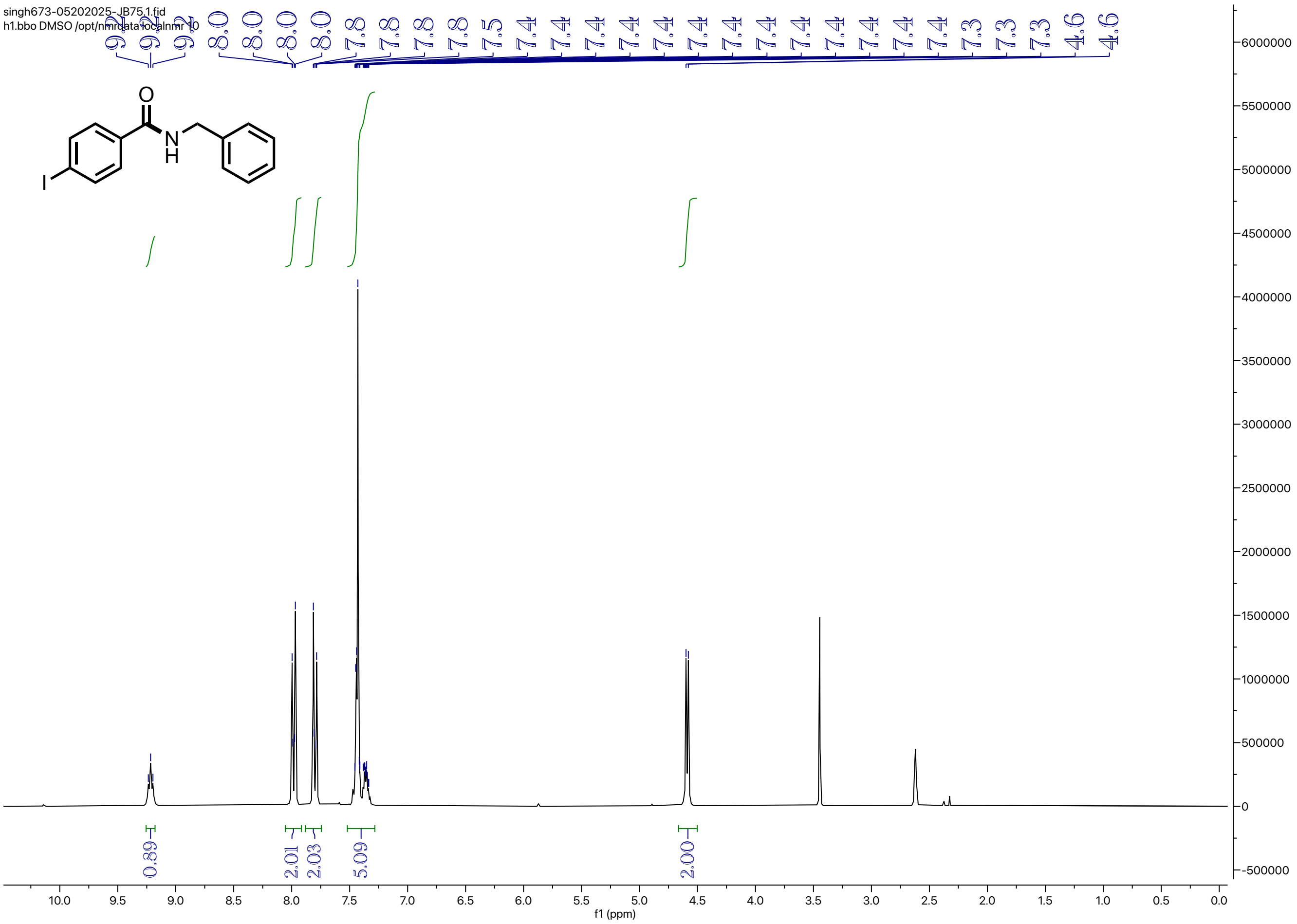

^{1}H NMR (300 MHz, DMSO-*d6*) *N*-benzyl-4-iodobenzamide (**6**)

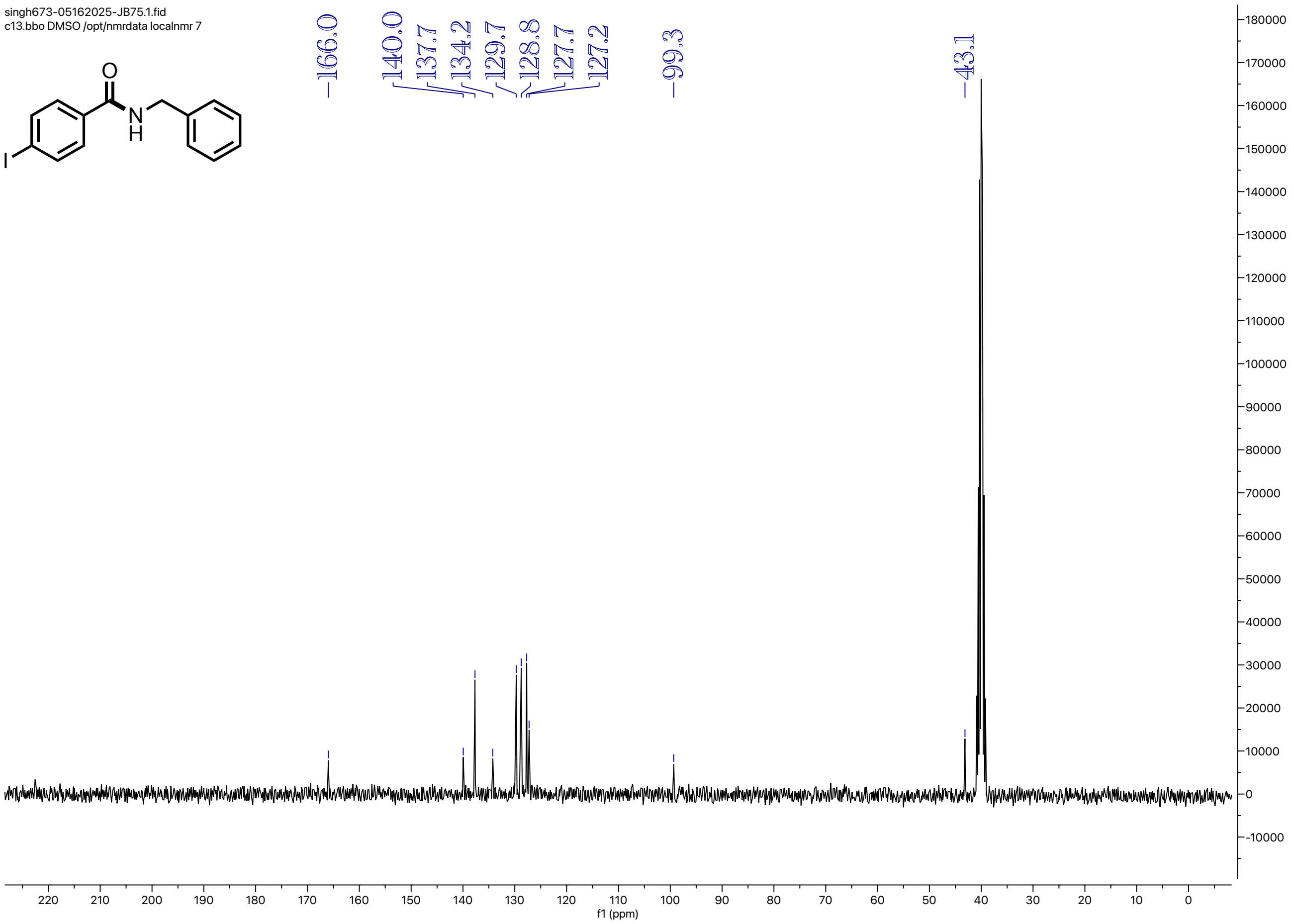

^{13}C {^{1}H} NMR (75 MHz, DMSO-*d6*) *N*-benzyl-4-iodobenzamide (**6**)

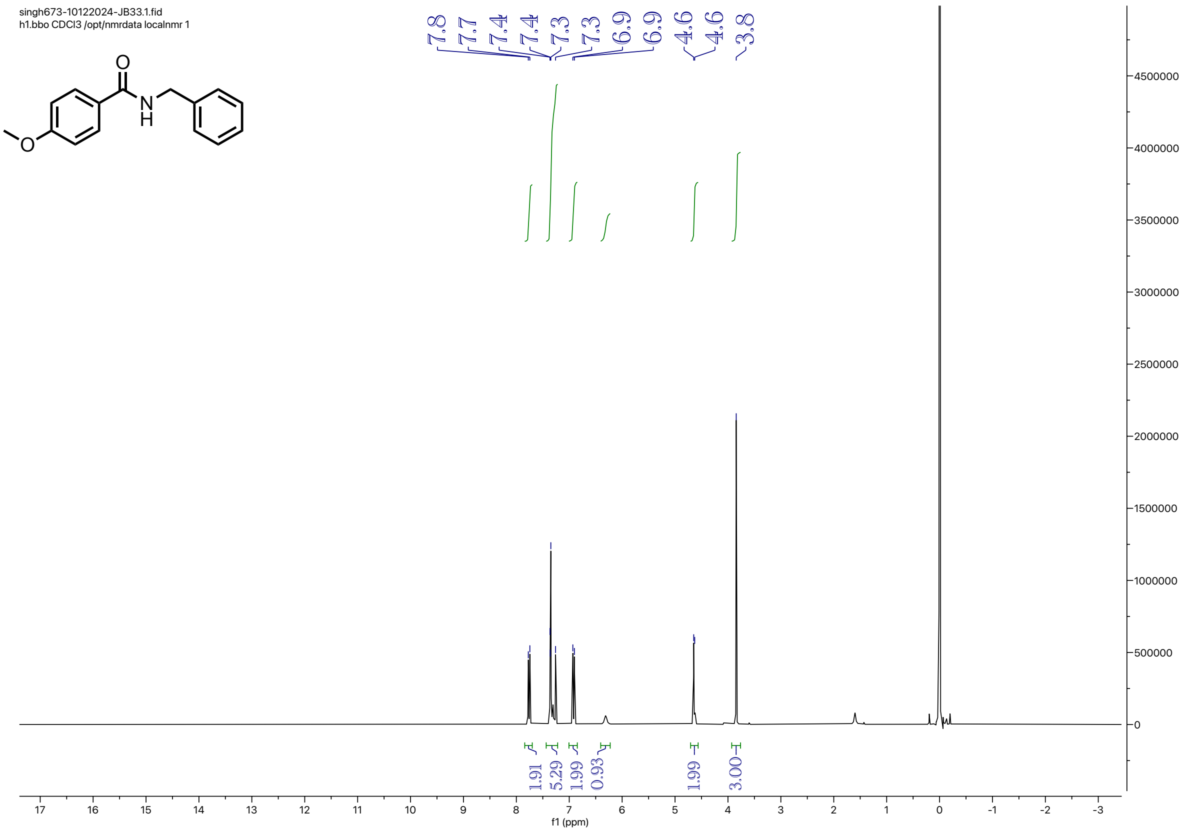

^{1}H NMR (300 MHz, Chloroform-*d*) *N*-benzyl-4-methoxybenzamide (**7**)

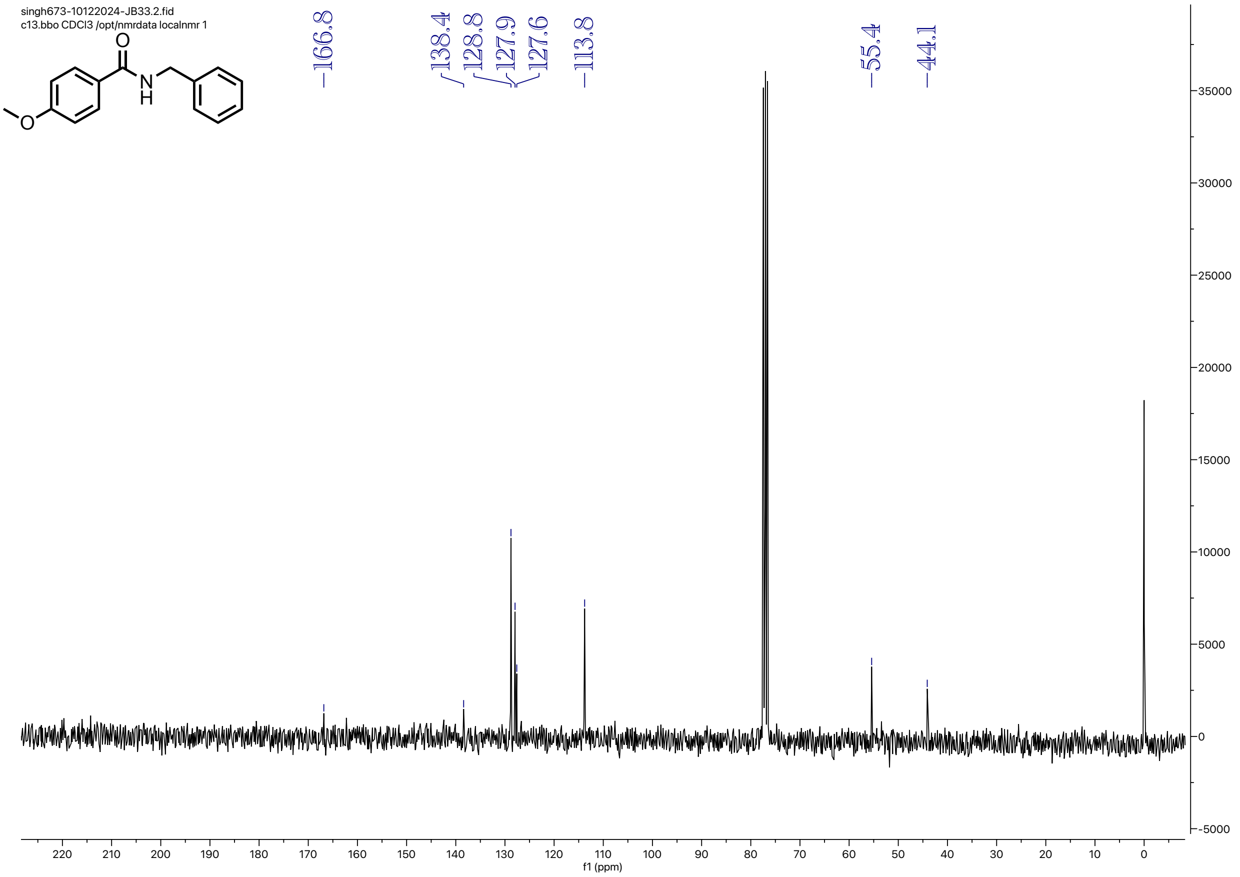

^{13}C {^{1}H} NMR (75 MHz, Chloroform-*d*) *N*-benzyl-4-methoxybenzamide (**7**)

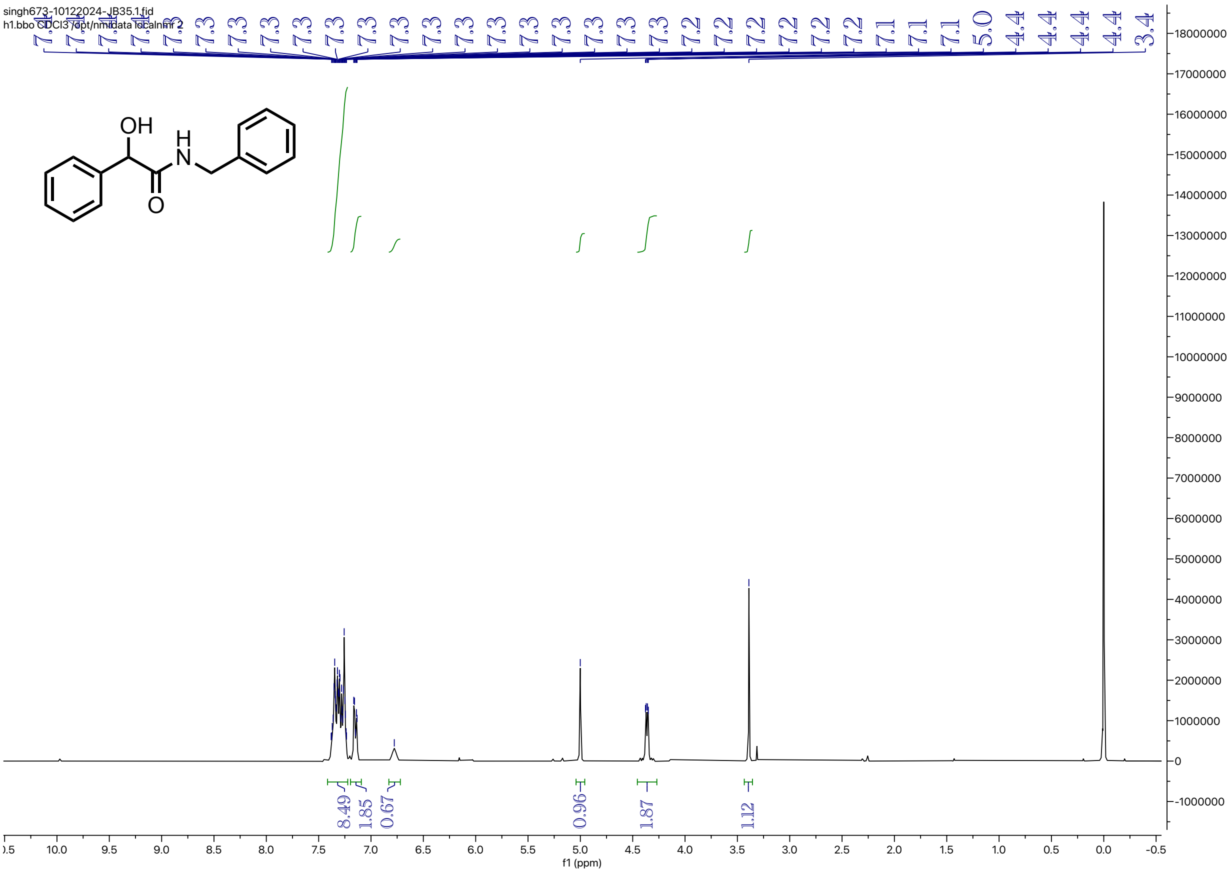

^{1}H NMR (300 MHz, Chloroform-*d*) *N*-benzyl-2-hydroxy-2-phenylacetamide (**8**)

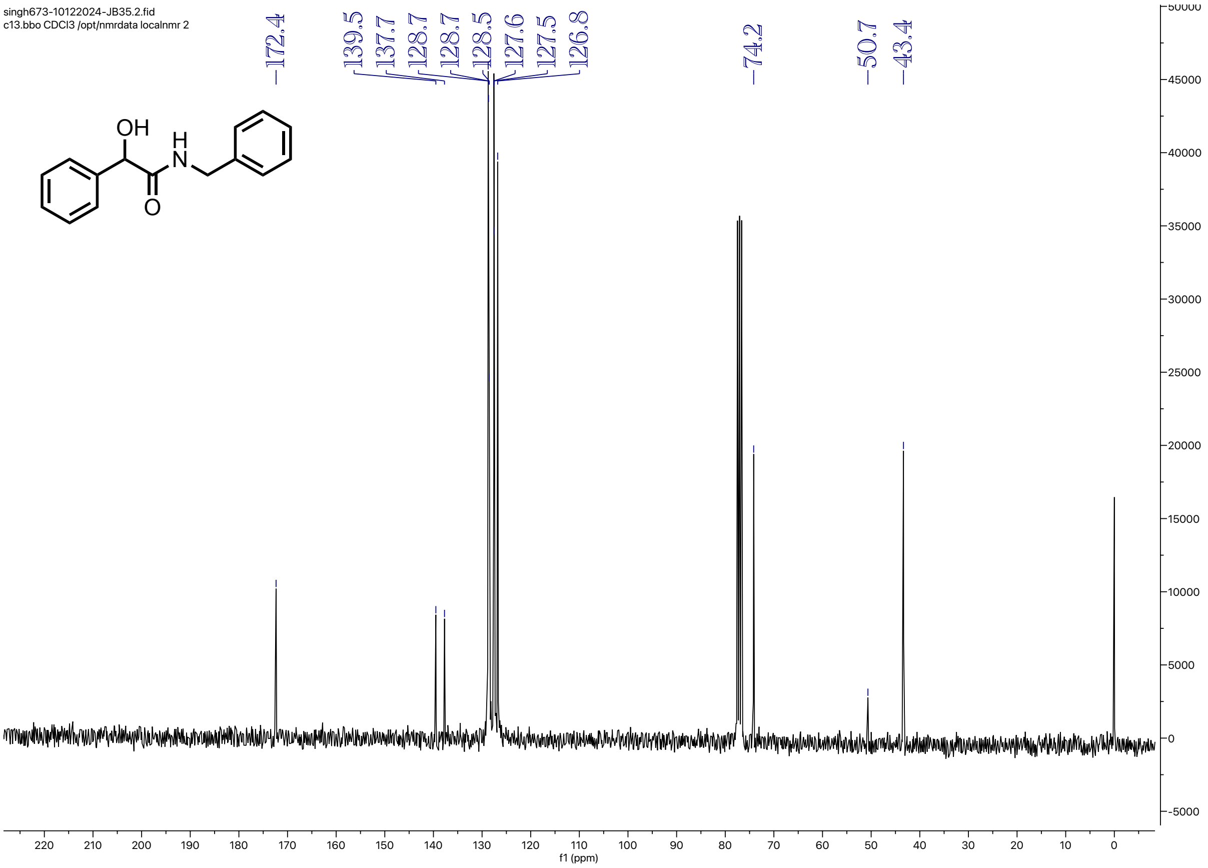

^{13}C {^{1}H} NMR (75 MHz, Chloroform-*d*) *N*-benzyl-2-hydroxy-2-phenylacetamide (**8**)

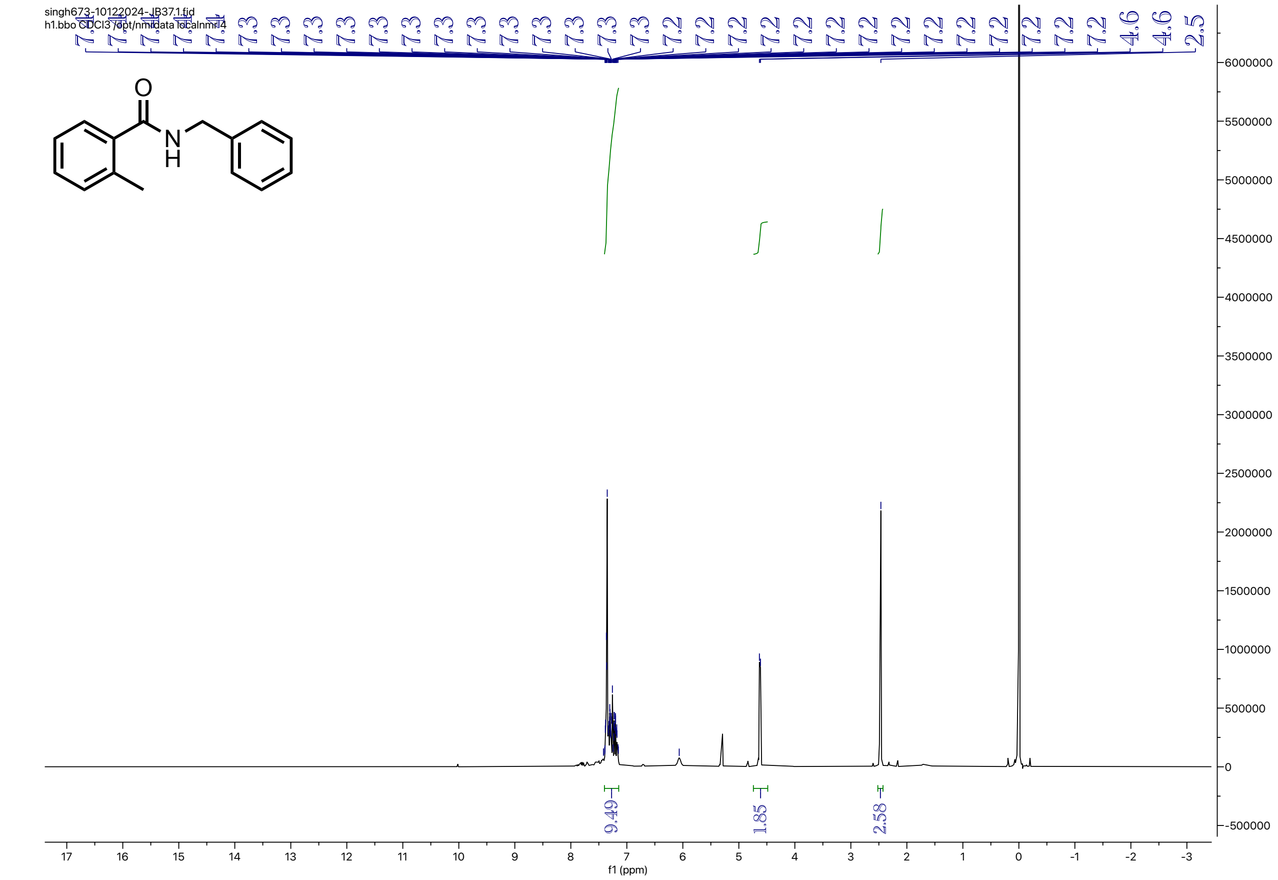

^{1}H NMR (300 MHz, Chloroform-*d*) *N*-benzyl-2-methylbenzamide (**9**)

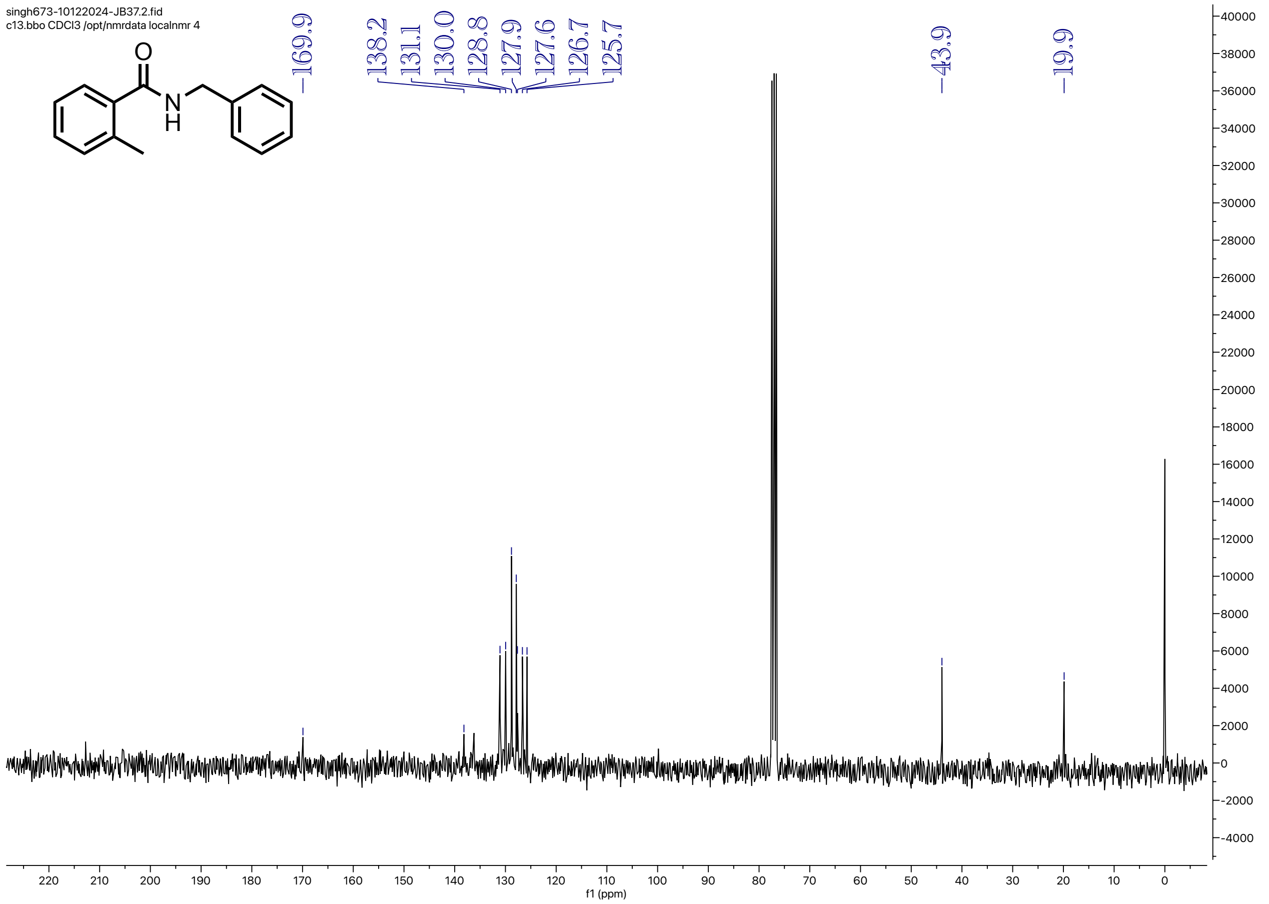

^{13}C {^{1}H} NMR (75 MHz, Chloroform-*d*) *N*-benzyl-2-methylbenzamide (**9**)

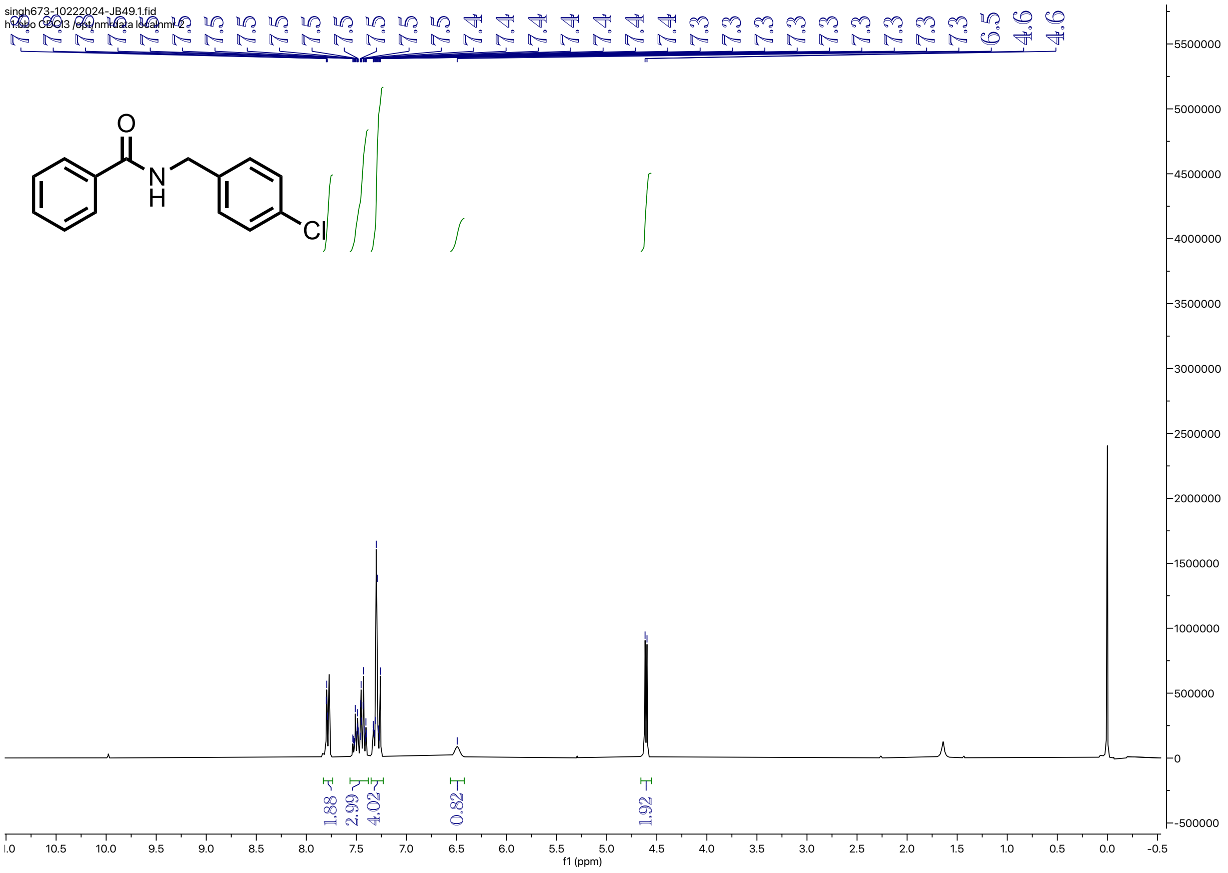

^{1}H NMR (300 MHz, Chloroform-*d*) *N*-(4-chlorobenzyl)benzamide (**10**)

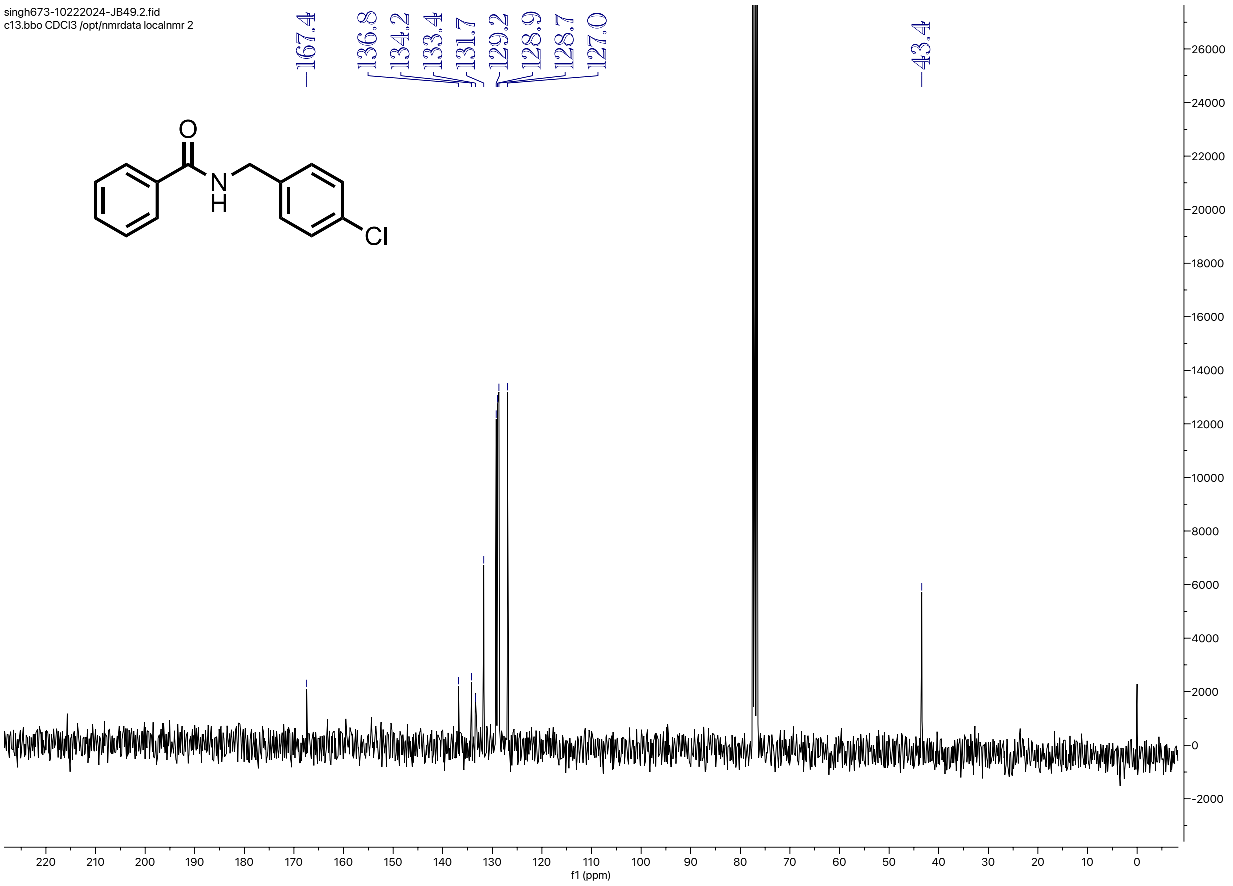

^{13}C {^{1}H} NMR (75 MHz, Chloroform-*d*) *N*-(4-chlorobenzyl)benzamide (**10**)

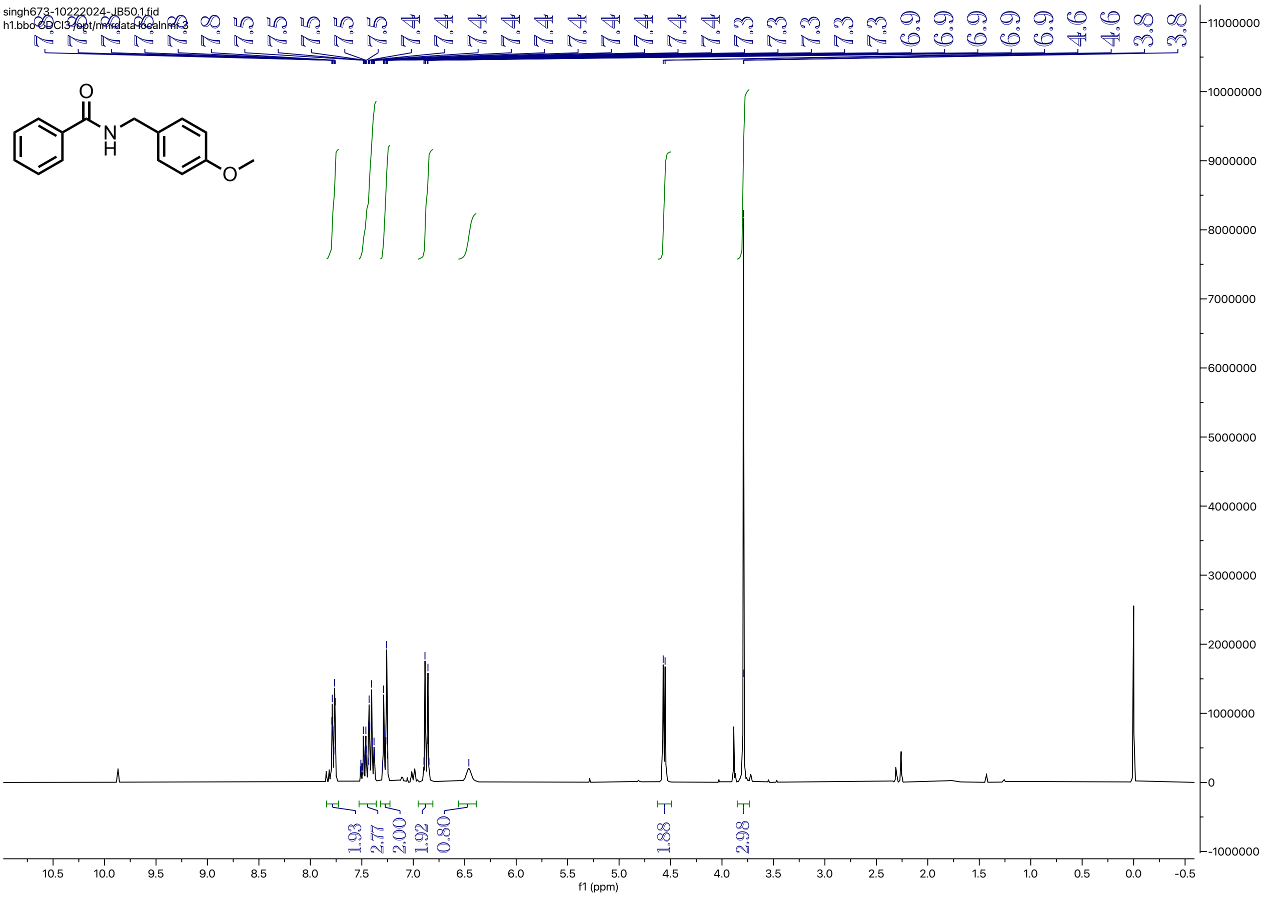

^{1}H NMR (300 MHz, Chloroform-*d*) *N*-(4-methoxybenzyl)benzamide (**11**)

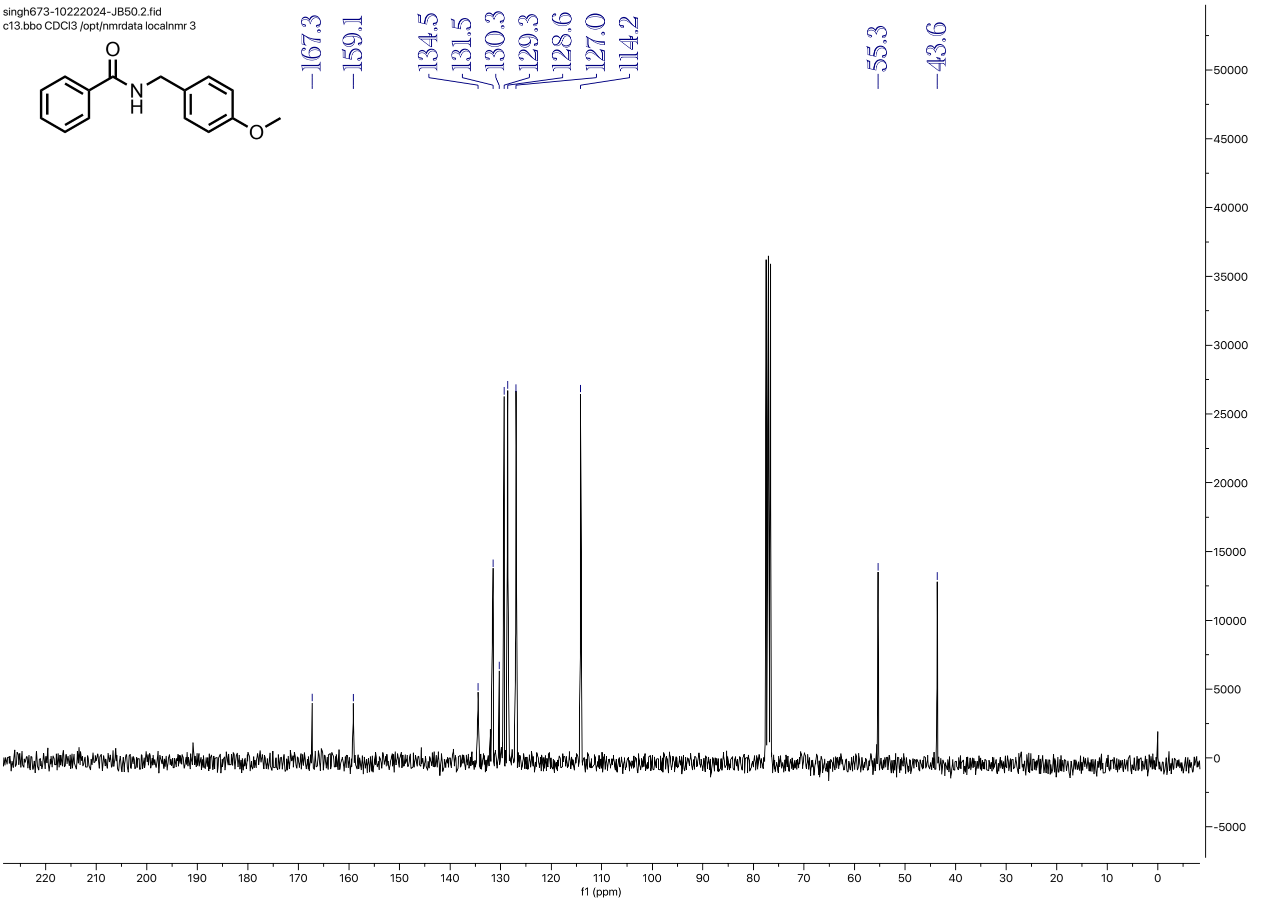

^{13}C {^{1}H} NMR (75 MHz, Chloroform-*d*) *N*-(4-methoxybenzyl)benzamide (**11**)

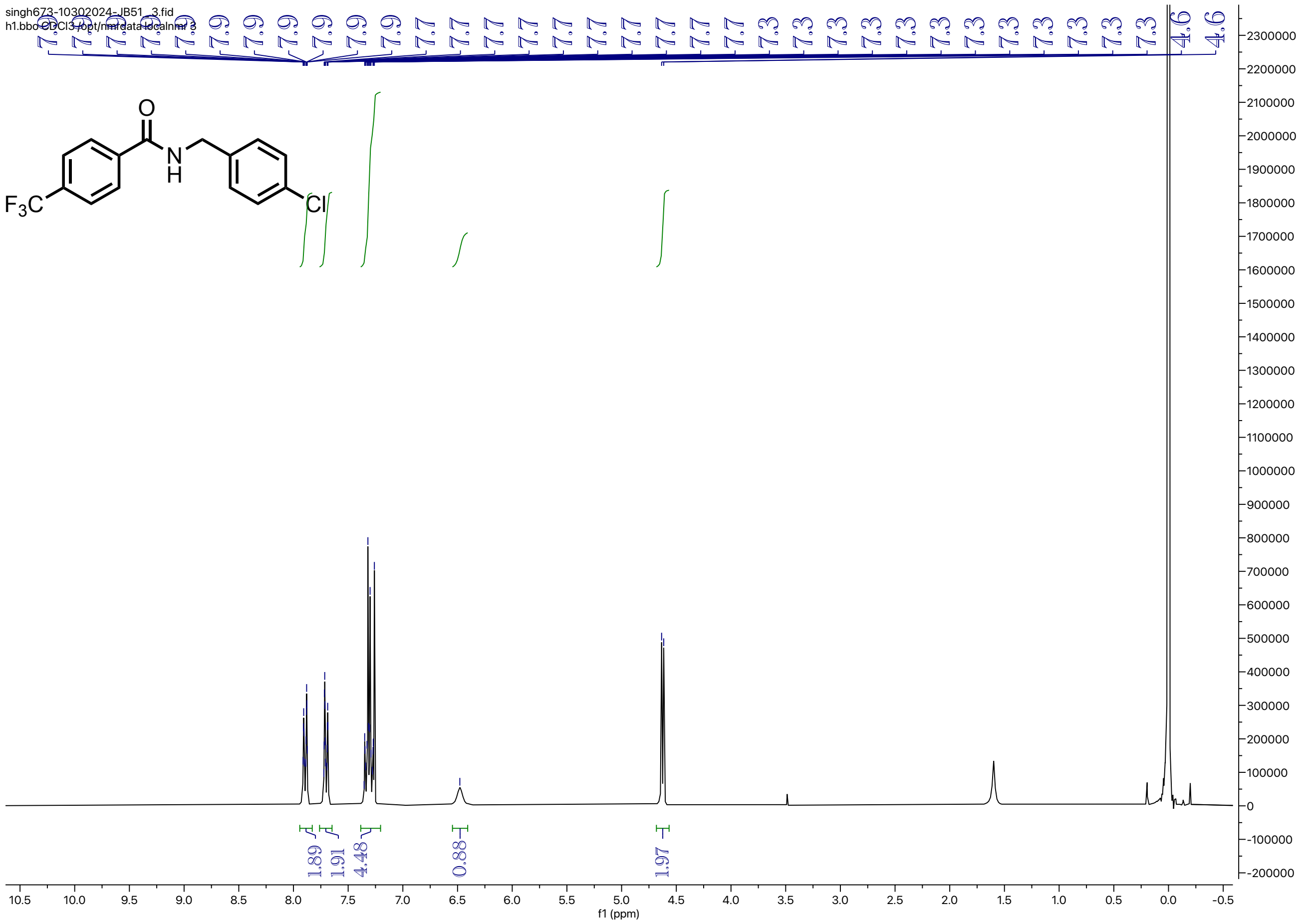

^{1}H NMR (300 MHz, Chloroform-*d*) *N*-(4-chlorobenzyl)-4-(trifluoromethyl)benzamide (**12**)

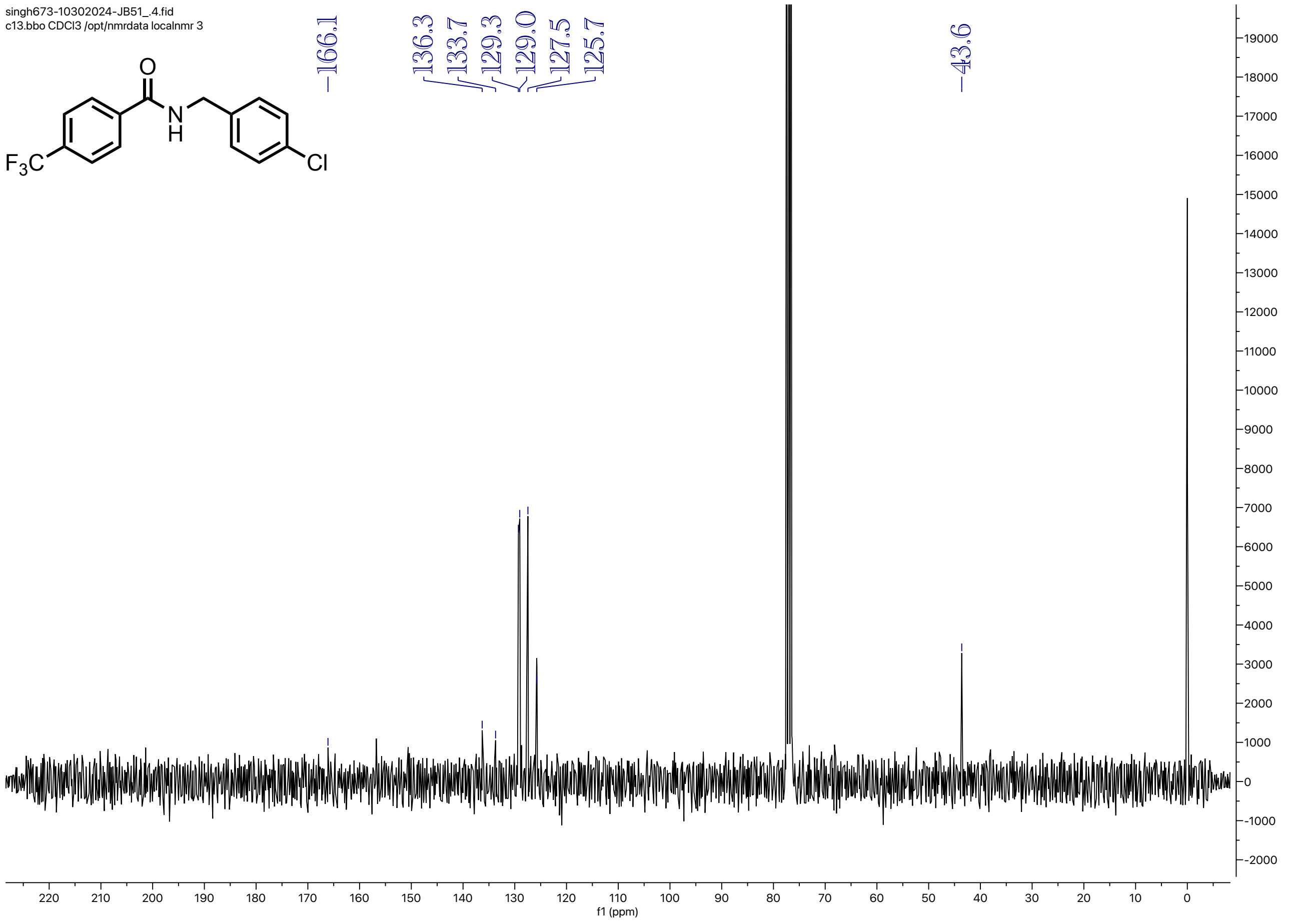

^{13}C {^{1}H} NMR (75 MHz, Chloroform-*d*) *N*-(4-chlorobenzyl)-4-(trifluoromethyl)benzamide (**12**)

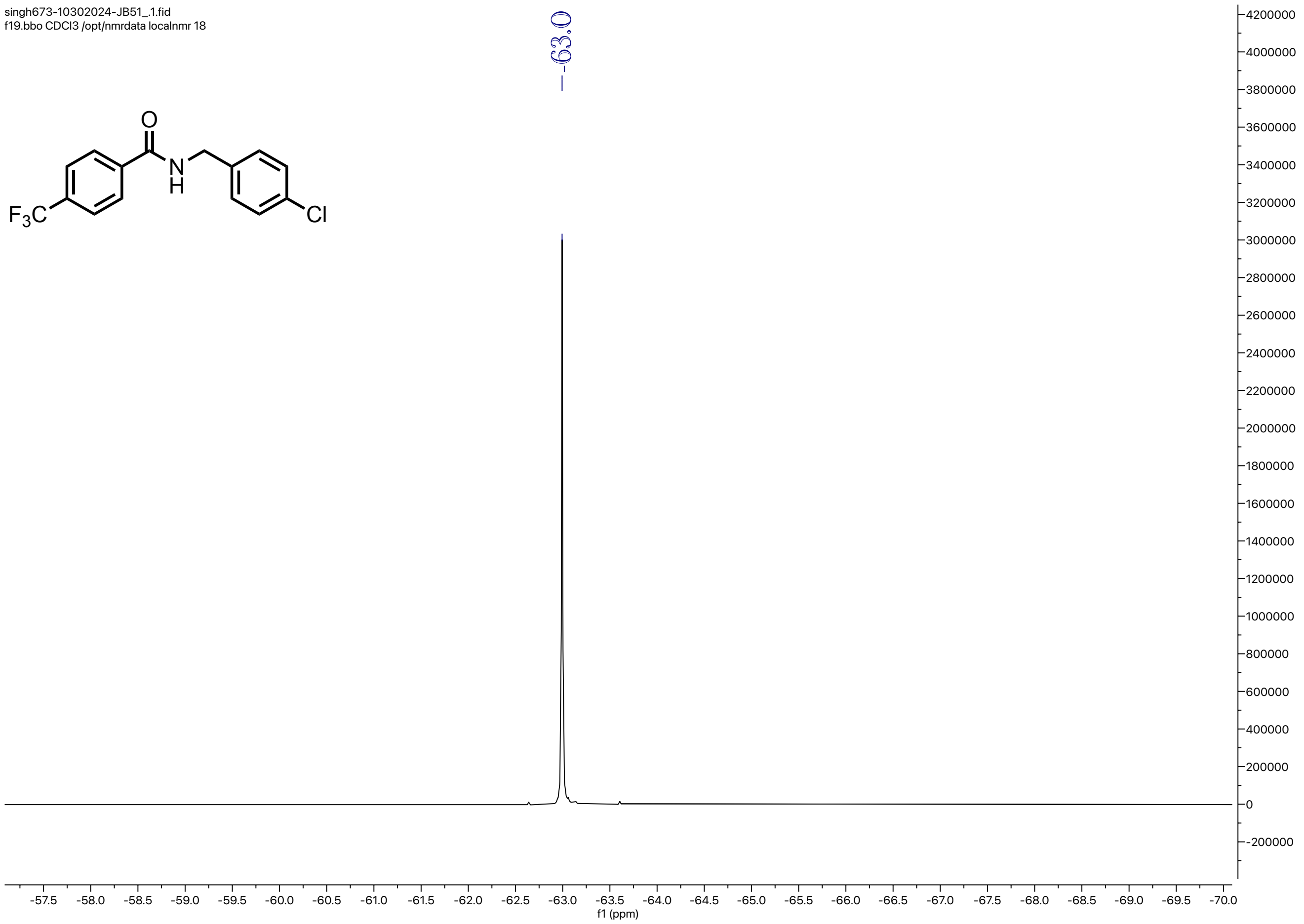

^{19}FNMR (282 MHz, Chloroform-*d*) *N*-(4-chlorobenzyl)-4-(trifluoromethyl)benzamide (**12**)

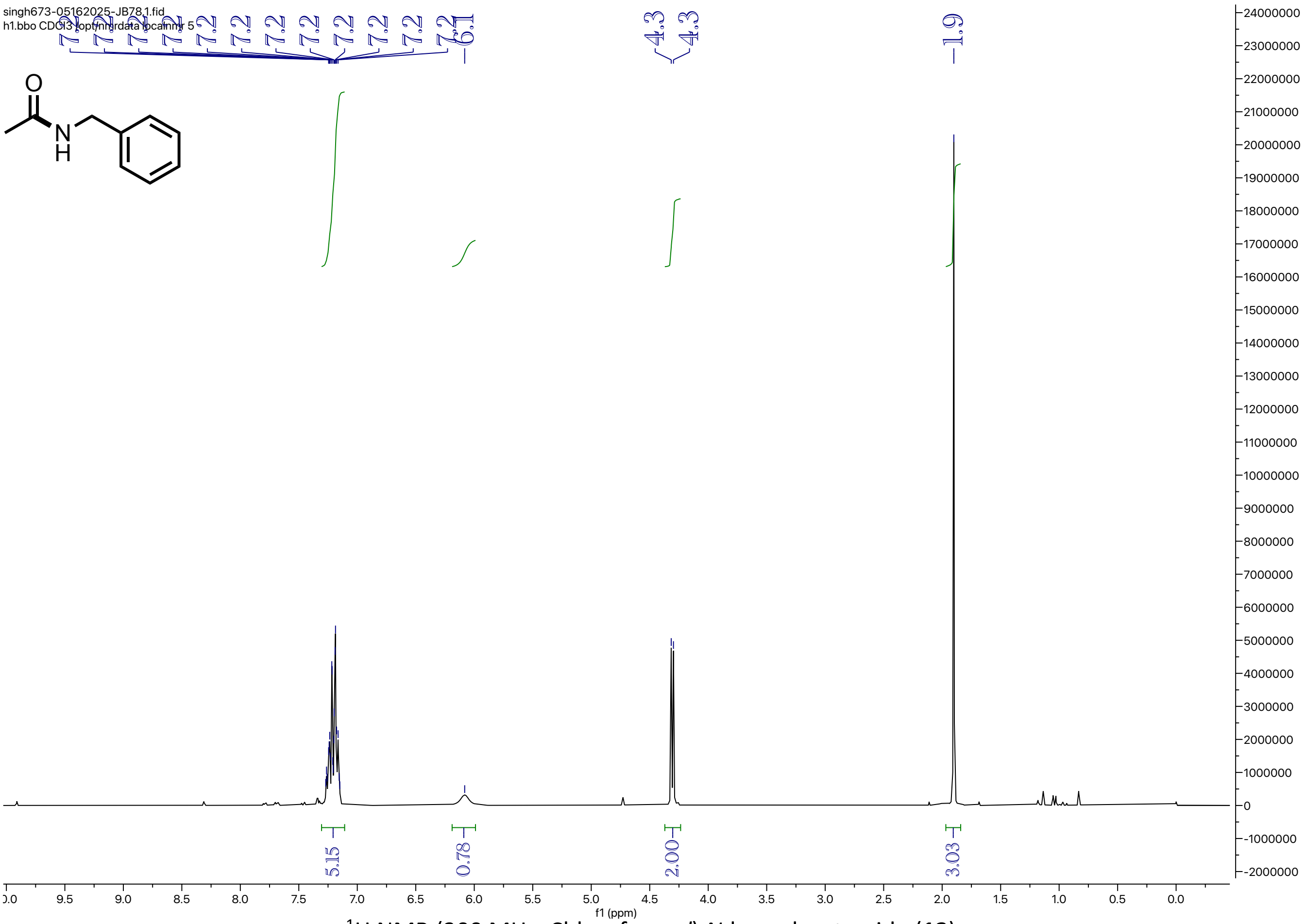

^{1}H NMR (300 MHz, Chloroform-*d*) *N*-benzylacetamide (**13**)

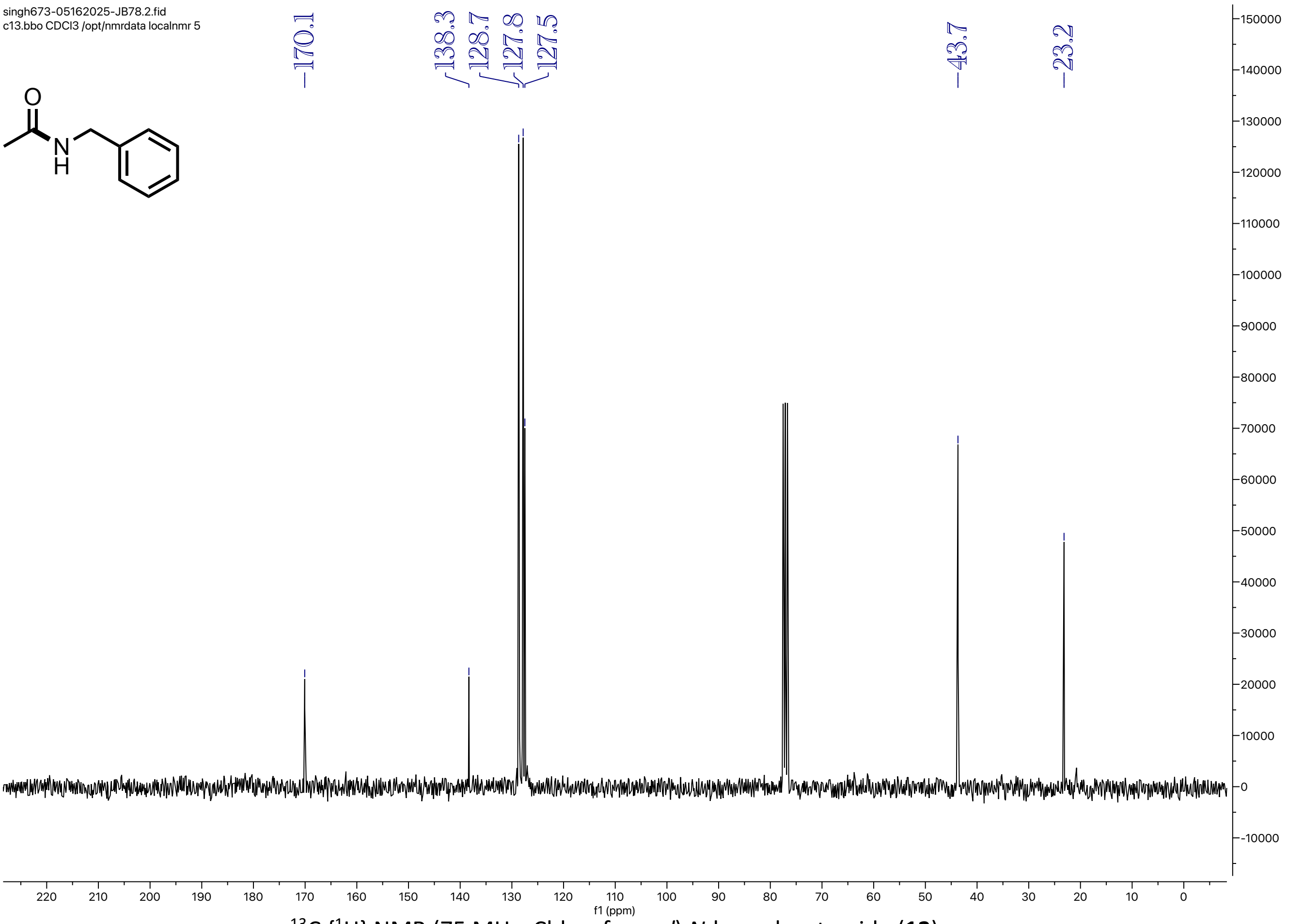

^{13}C {^{1}H} NMR (75 MHz, Chloroform-*d*) *N*-benzylacetamide (**13**)

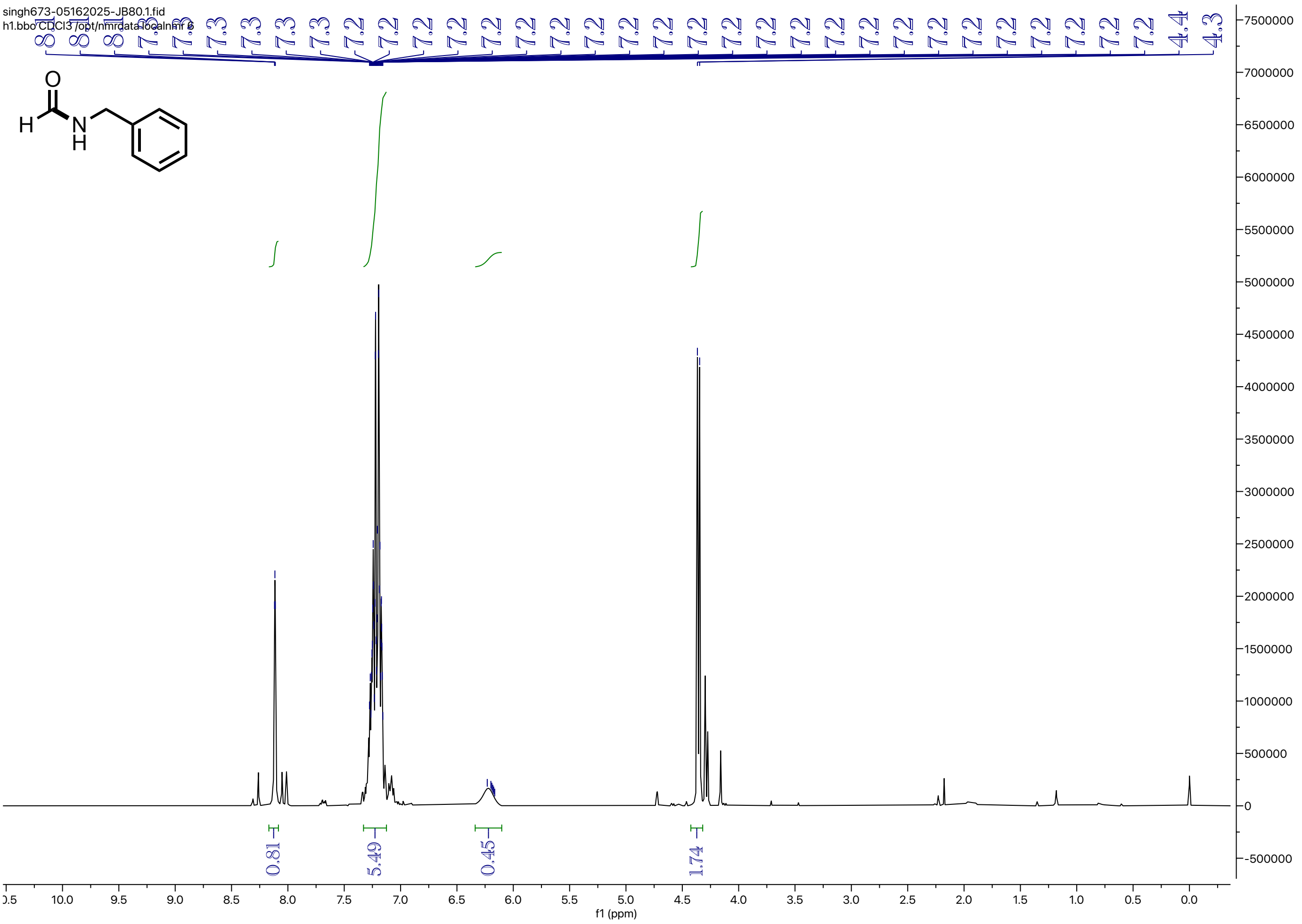

^{1}H NMR (300 MHz, Chloroform-*d*) *N*-benzylformamide (**14**)

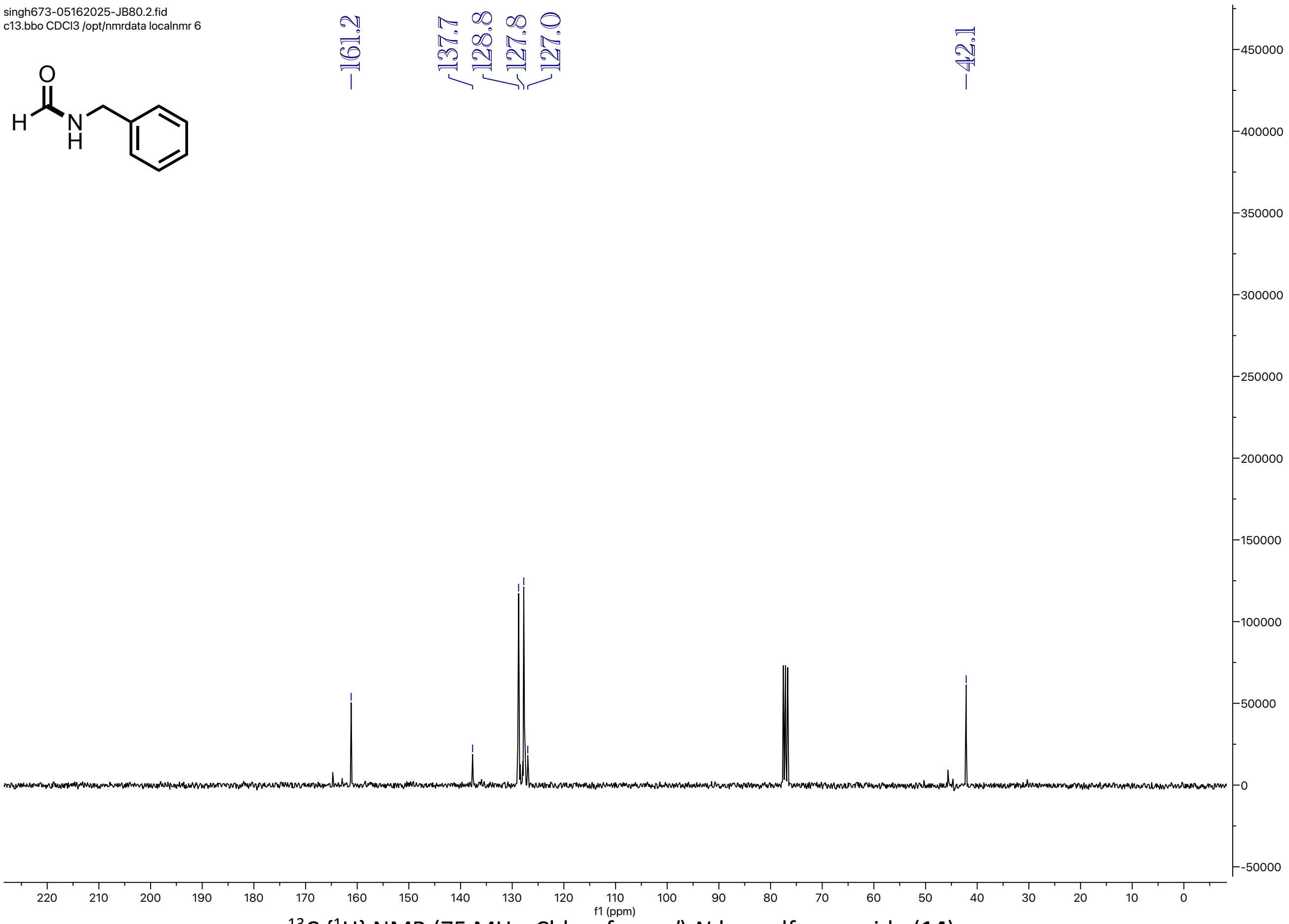

^{13}C {^{1}H} NMR (75 MHz, Chloroform-*d*) *N*-benzylformamide (**14**)

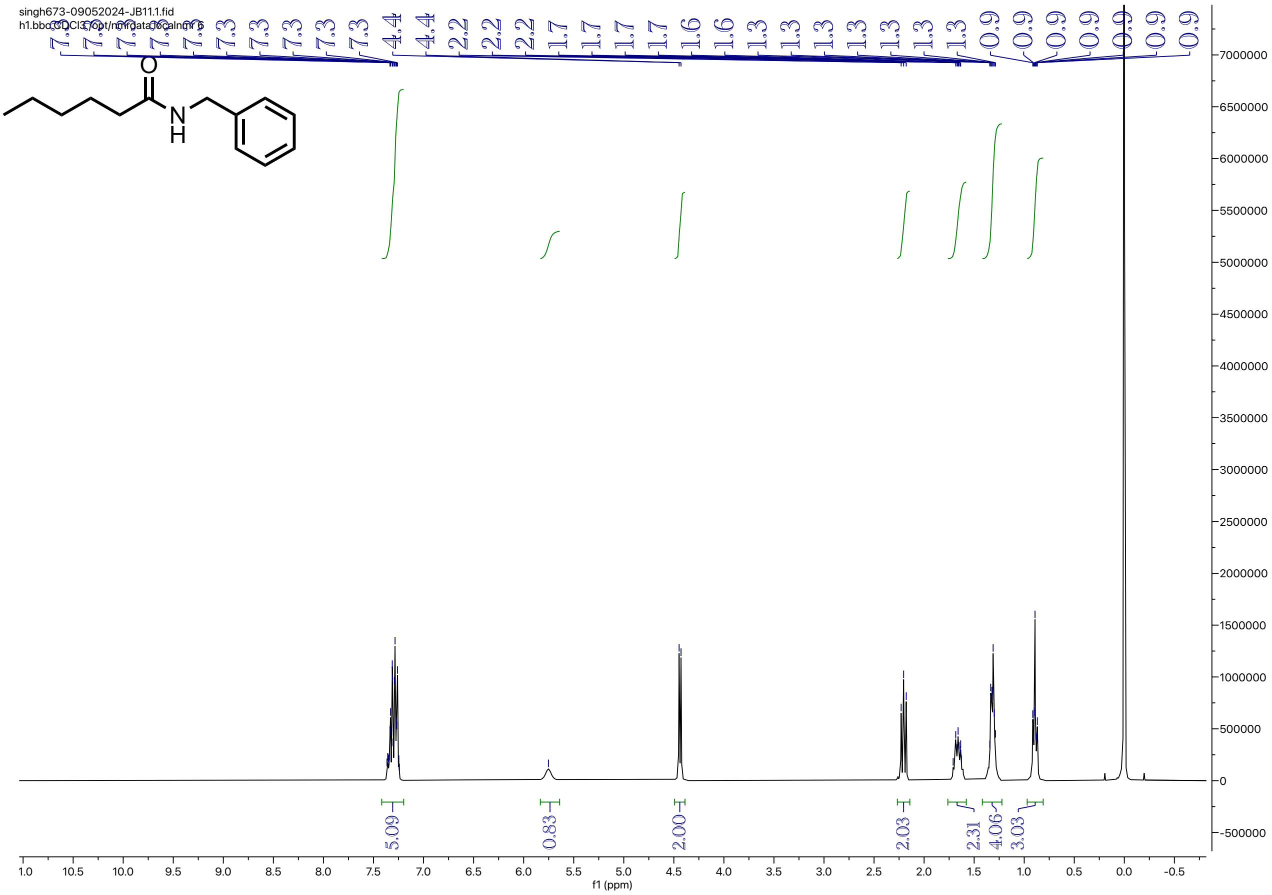

^{1}H NMR (300 MHz, Chloroform-*d*) *N*-benzylhexanamide (**15**)

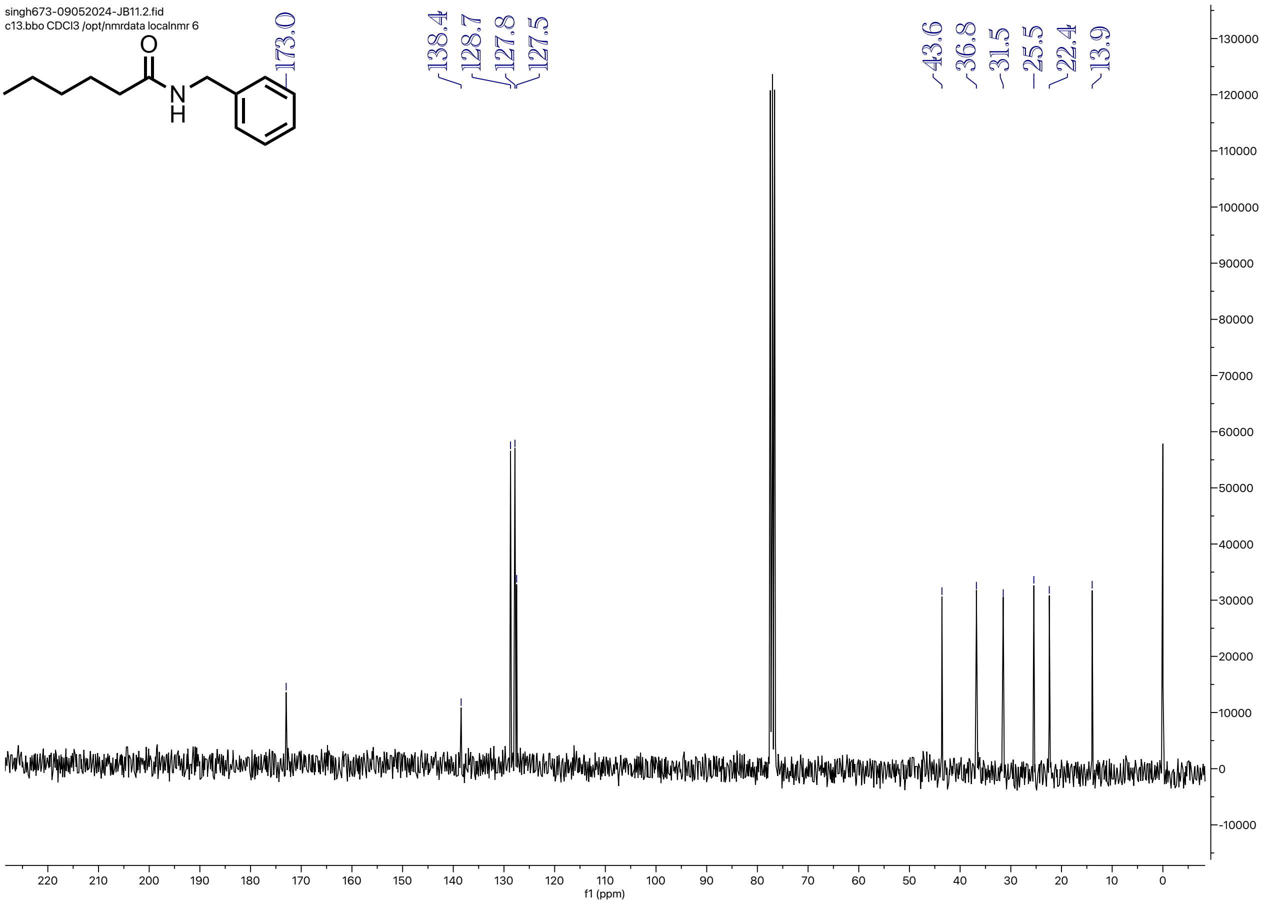

^{13}C {^{1}H} NMR (75 MHz, Chloroform-*d*) *N*-benzylhexanamide (**15**)

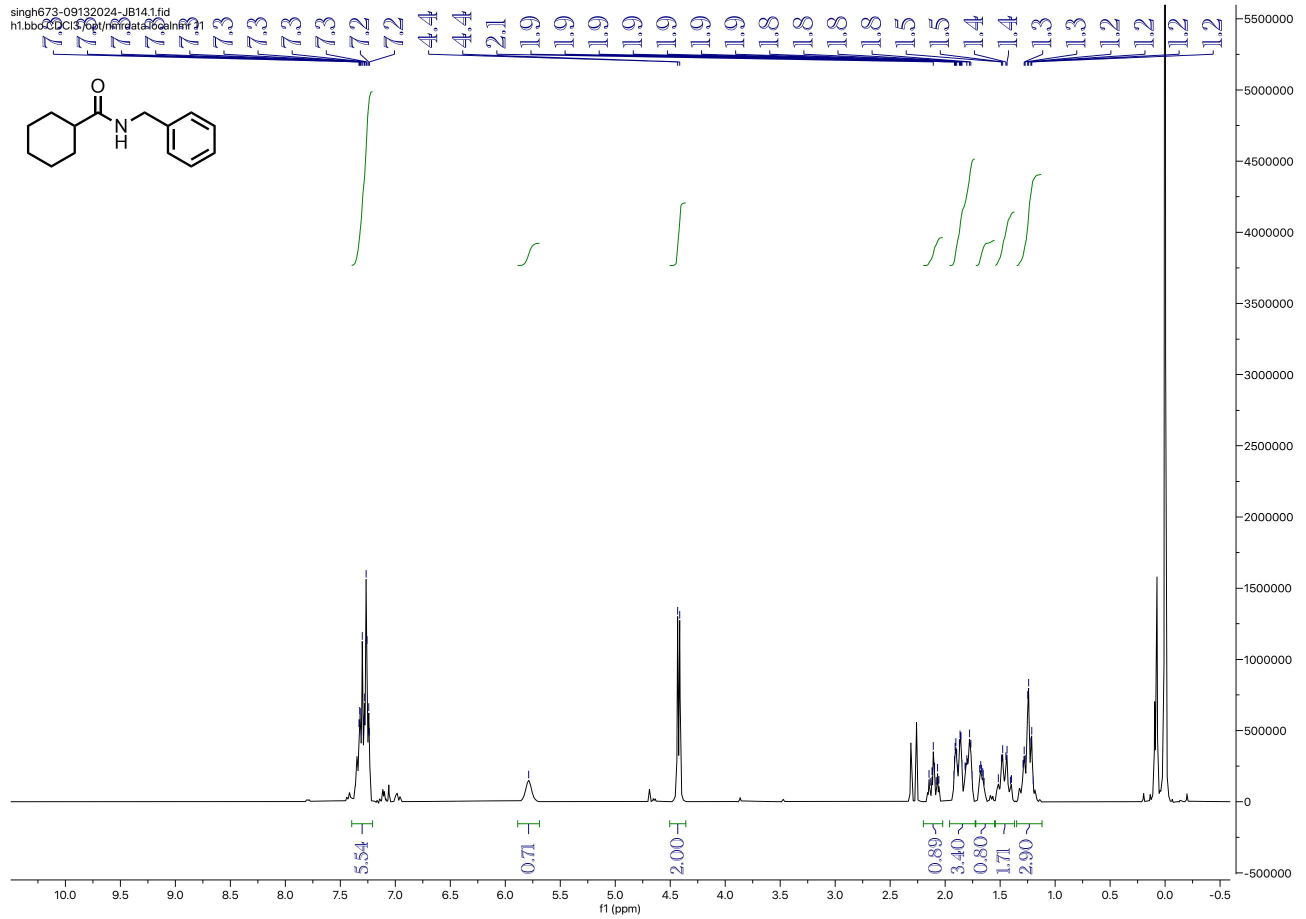

^{1}H NMR (300 MHz, Chloroform-*d*) *N*-benzylcyclohexanecarboxamide (**16**)

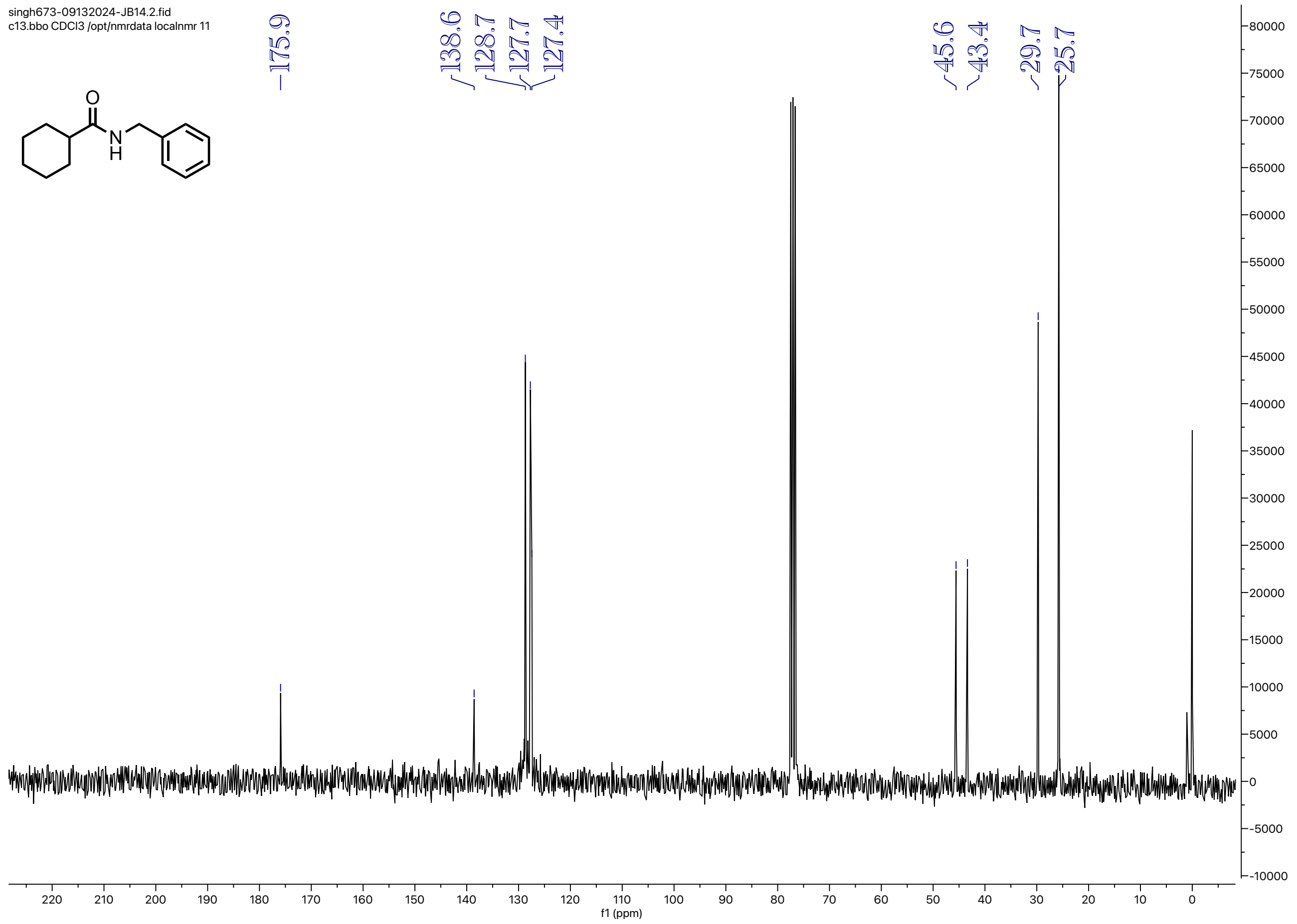

^{13}C {^{1}H} NMR (75 MHz, Chloroform-*d*) *N*-benzylcyclohexanecarboxamide (**16**)

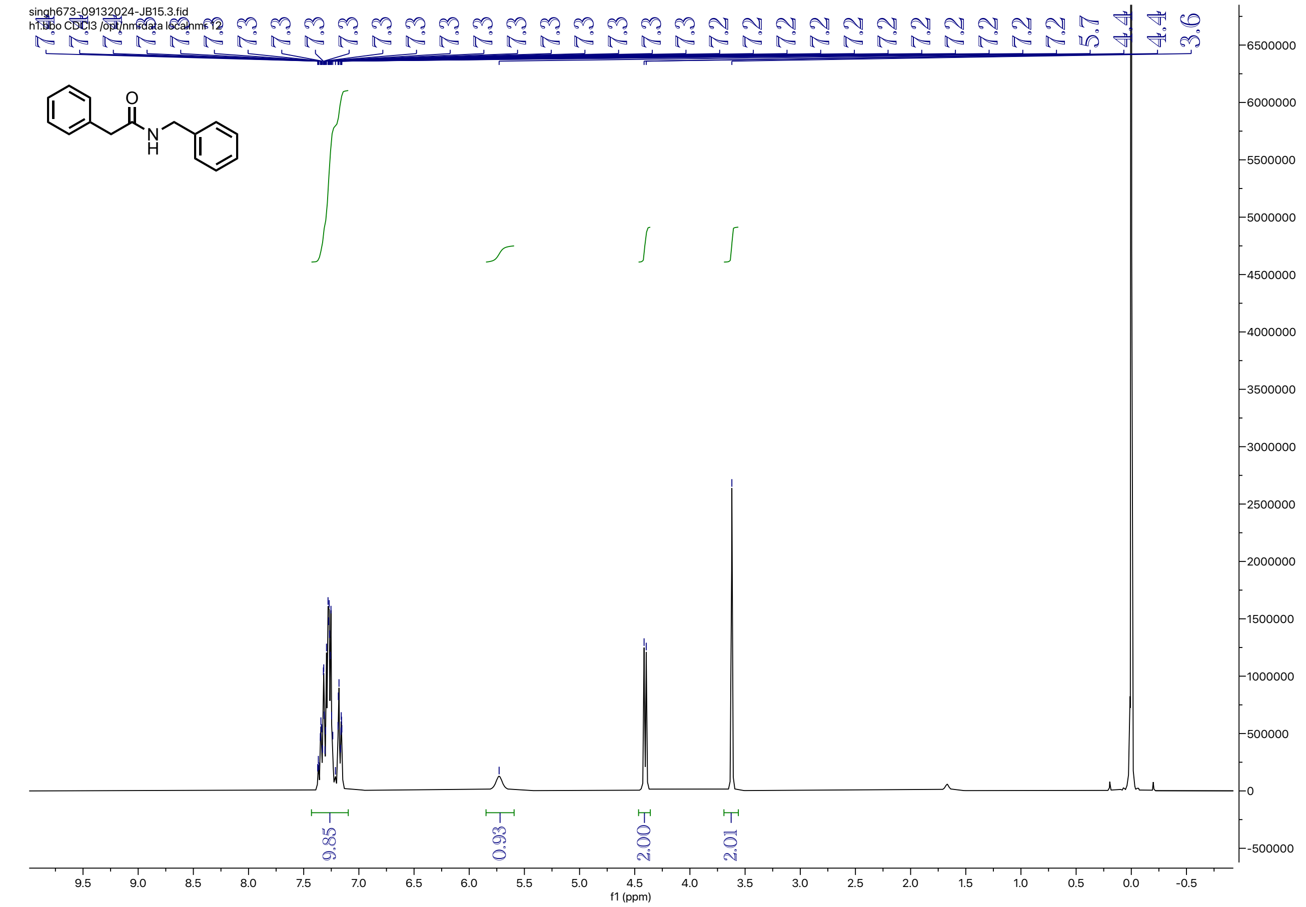

^{1}H NMR (300 MHz, Chloroform-*d*) *N*-benzyl-2-phenylacetamide (**17**)

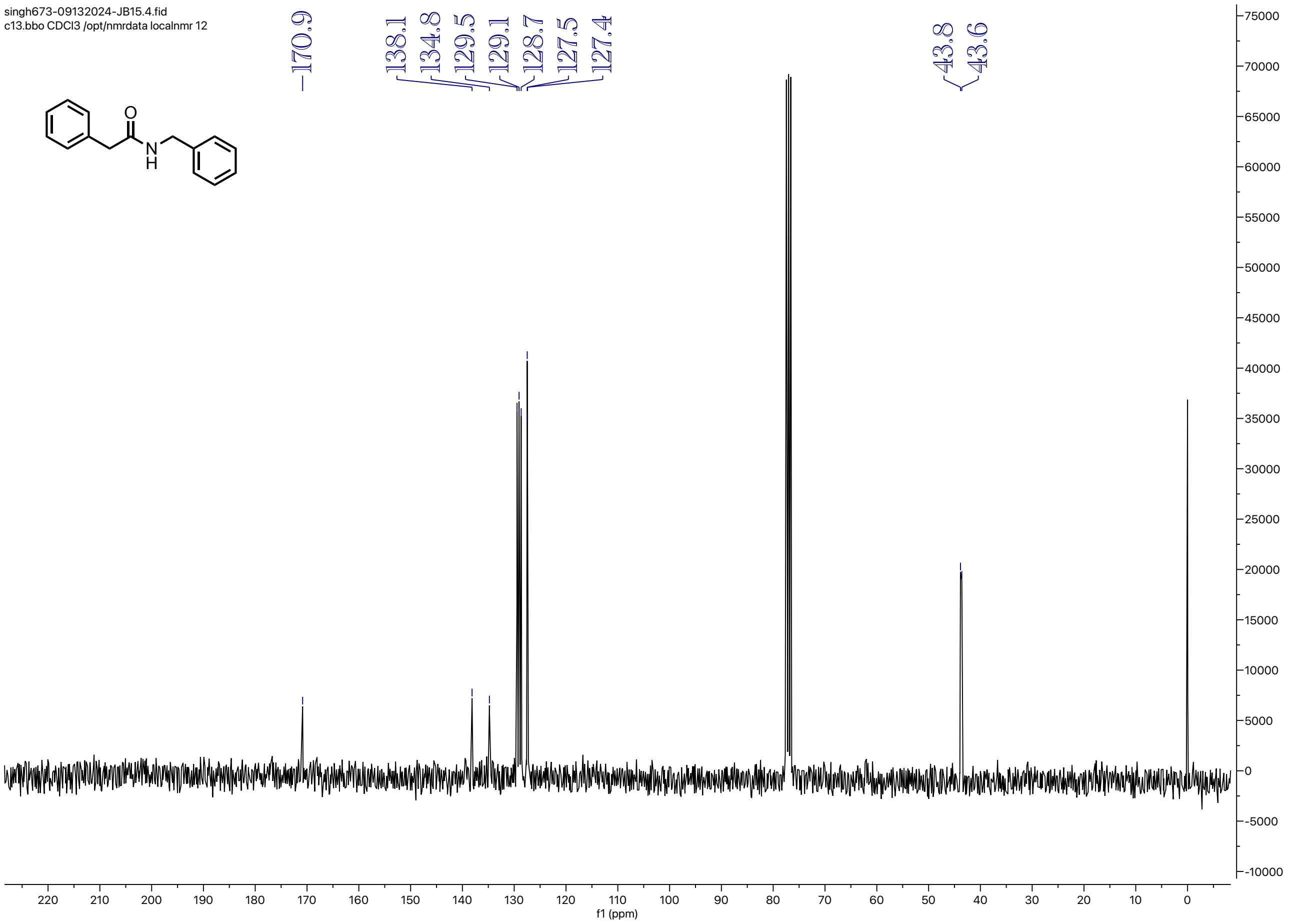

^{13}C {^{1}H} NMR (75 MHz, Chloroform-*d*) *N*-benzyl-2-phenylacetamide (**17**)

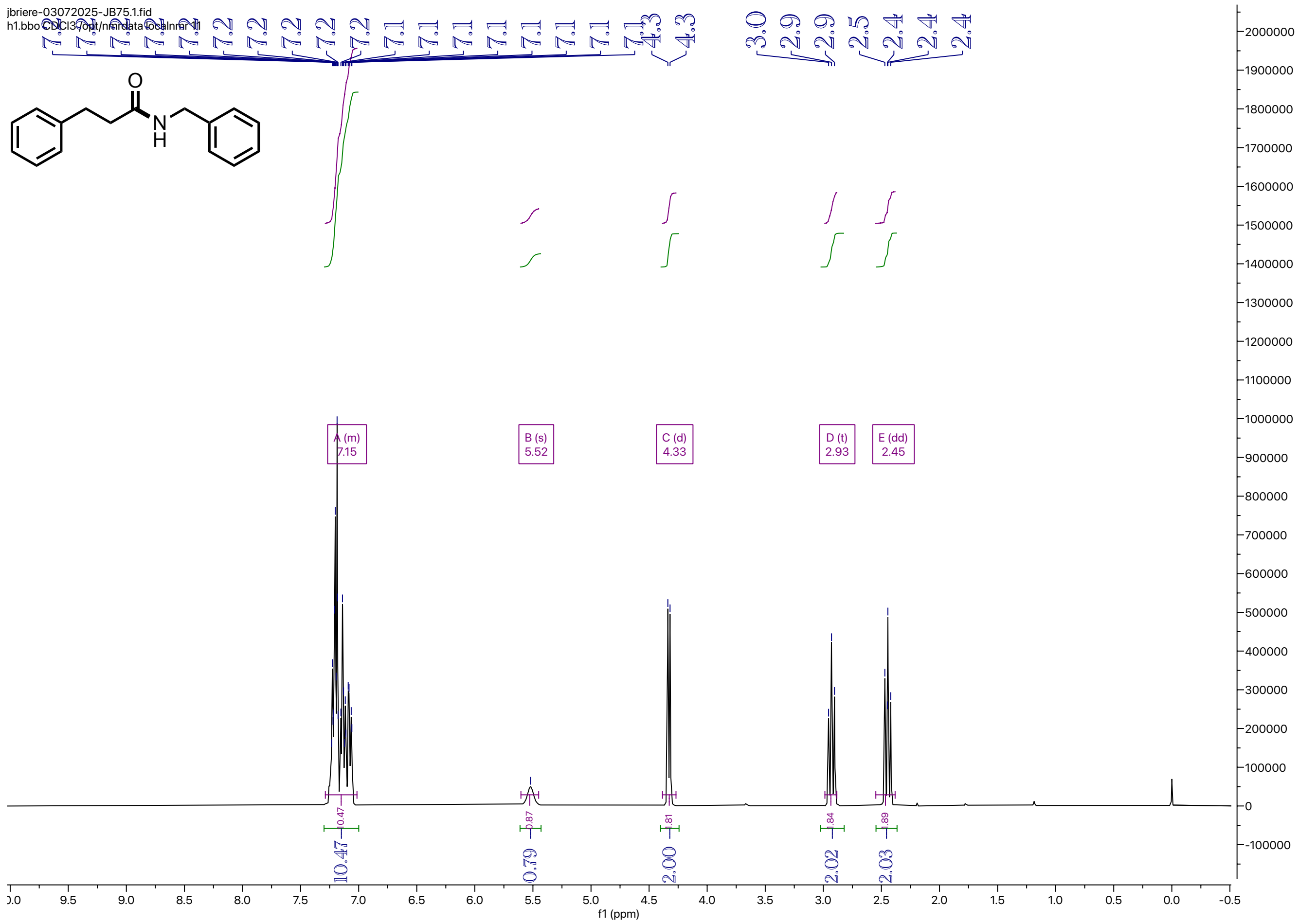

^{1}H NMR (300 MHz, Chloroform-*d*) *N*-benzyl-2-phenylacetamide (**18**)

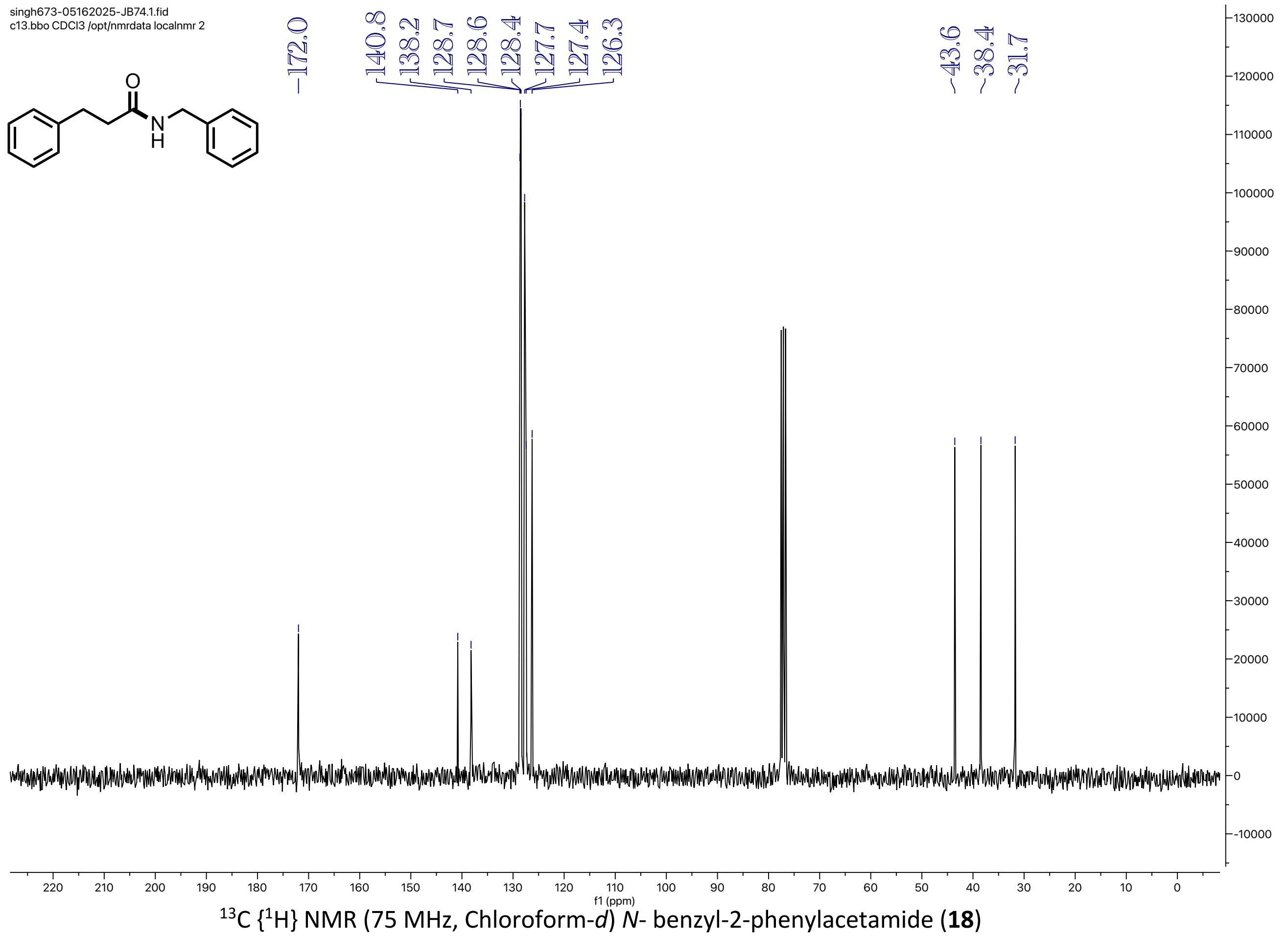

^{13}C {^{1}H} NMR (75 MHz, Chloroform-*d*) *N*- benzyl-2-phenylacetamide (**18**)

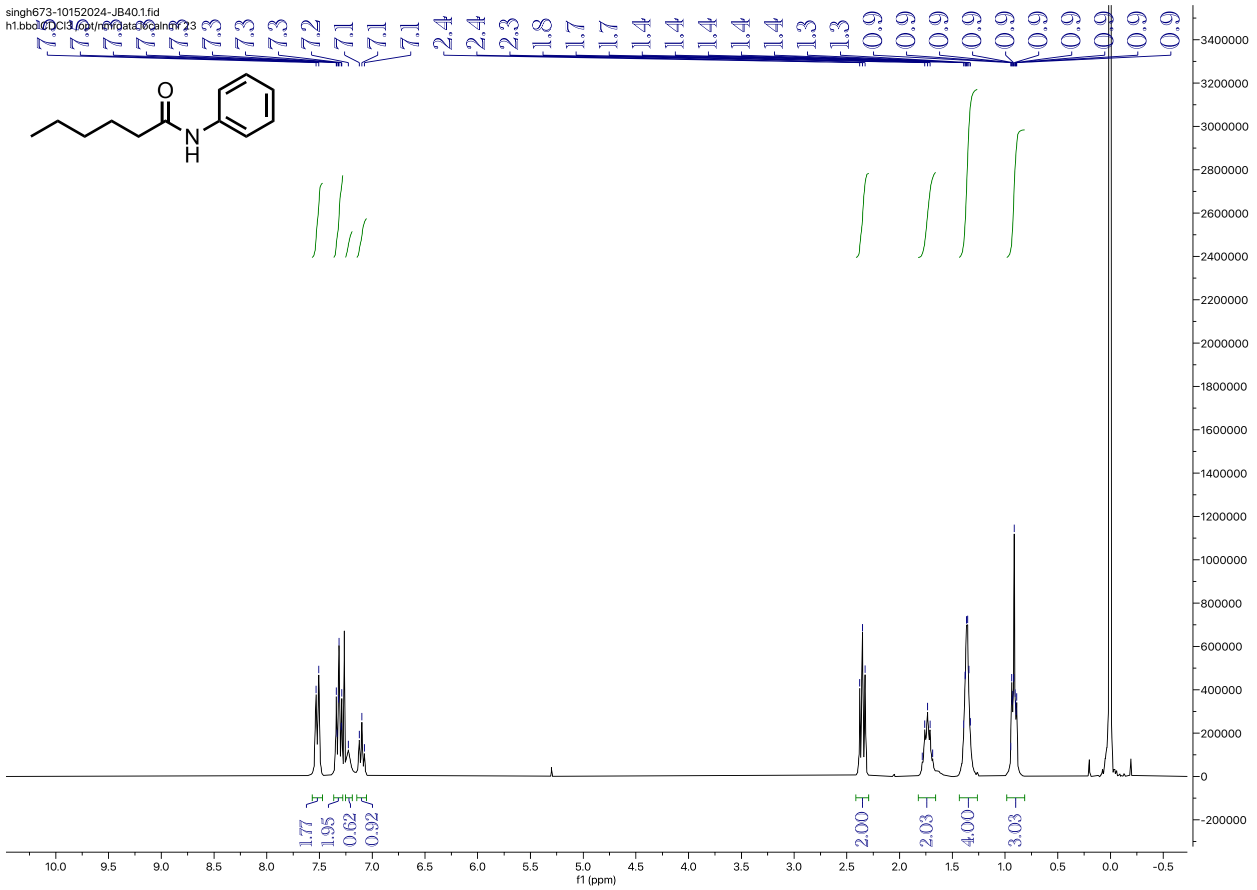

^{1}H NMR (300 MHz, Chloroform-*d*) *N*-phenylhexanamide (**19**)

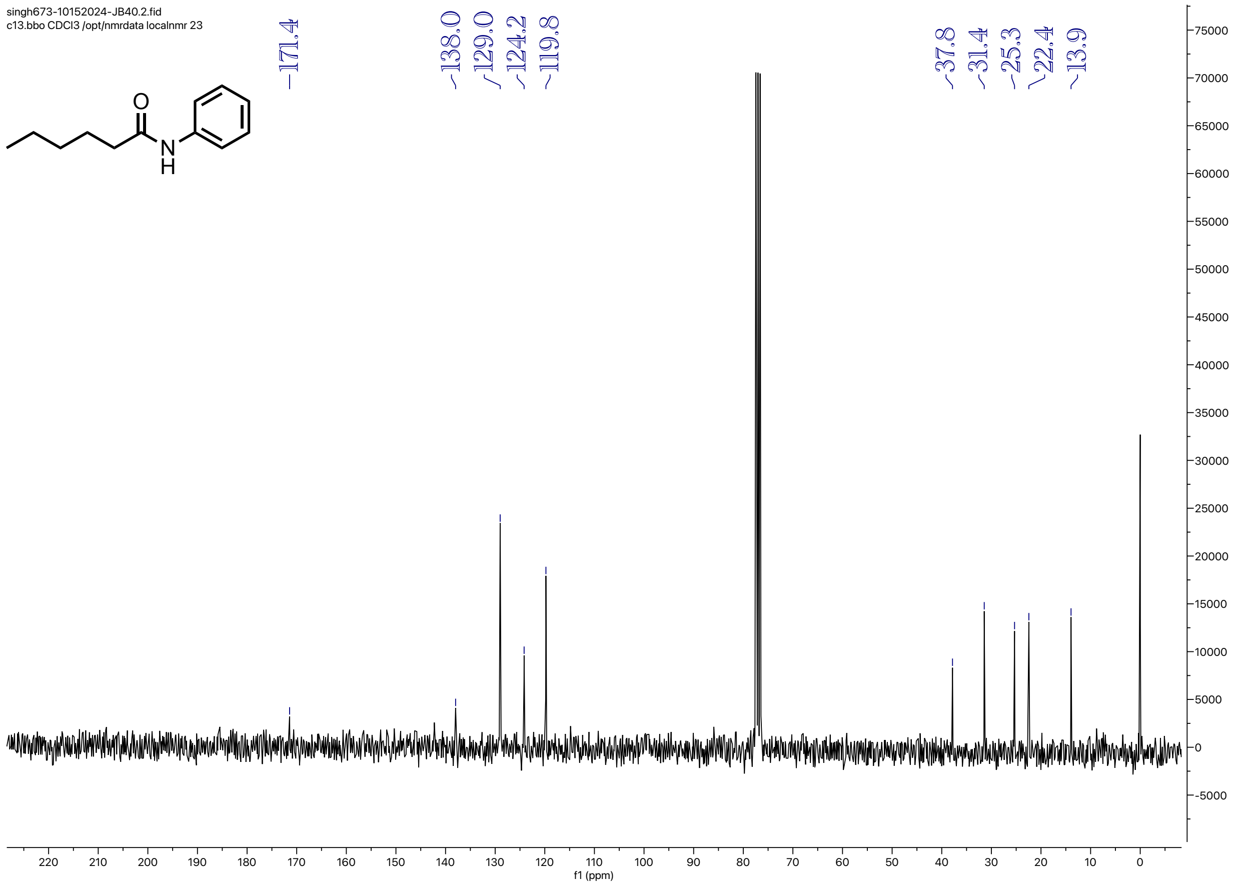

^{13}C {^{1}H} NMR (75 MHz, Chloroform-*d*) *N*-phenylhexanamide (**19**)

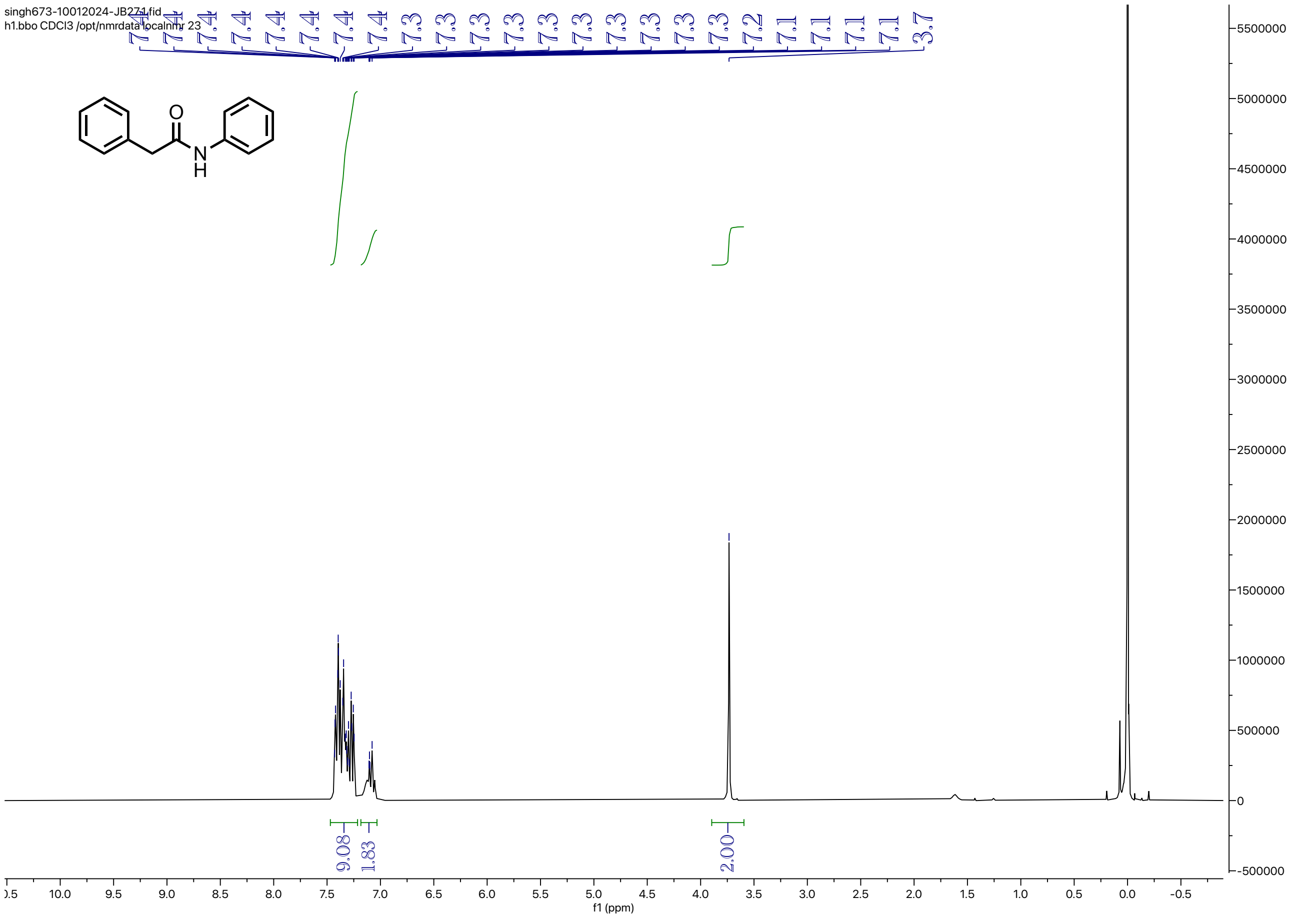

^1H NMR (300 MHz, Chloroform-*d*) *N*,2-diphenylacetamide (**20**)

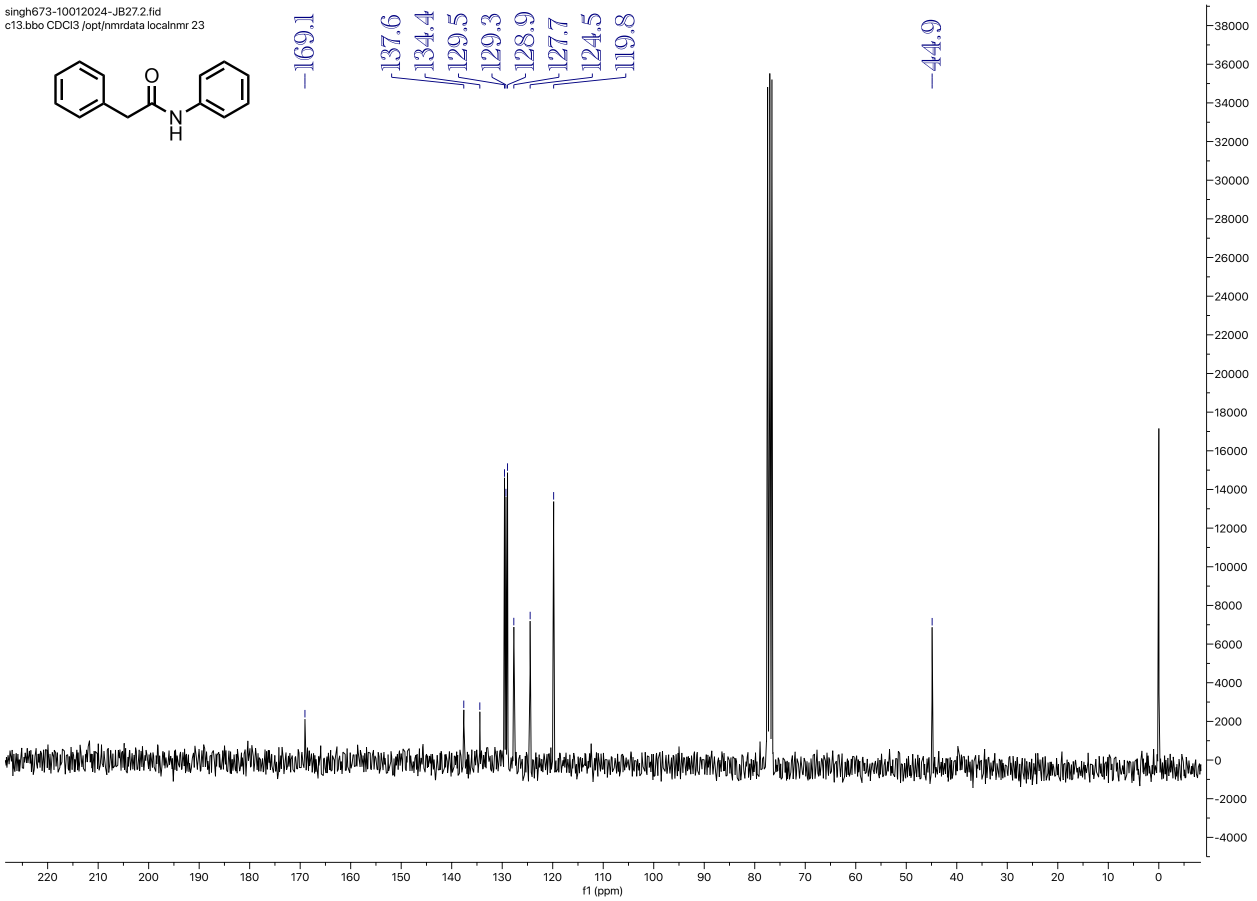

^{13}C {^1H} NMR (75 MHz, Chloroform-*d*) *N*,2-diphenylacetamide (**20**)

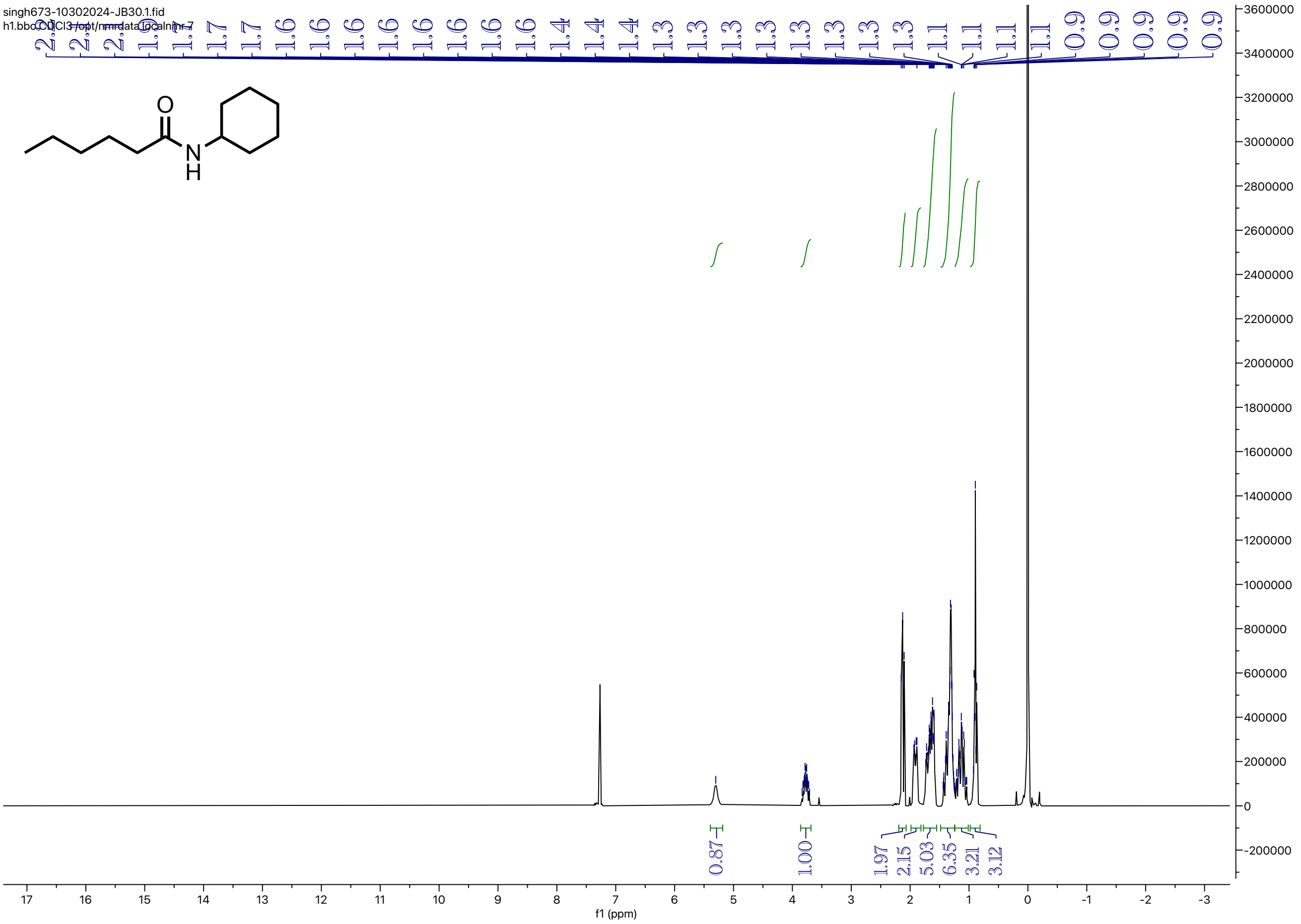

^{1}H NMR (300 MHz, Chloroform-*d*) *N*-cyclohexylhexanamide (**21**)

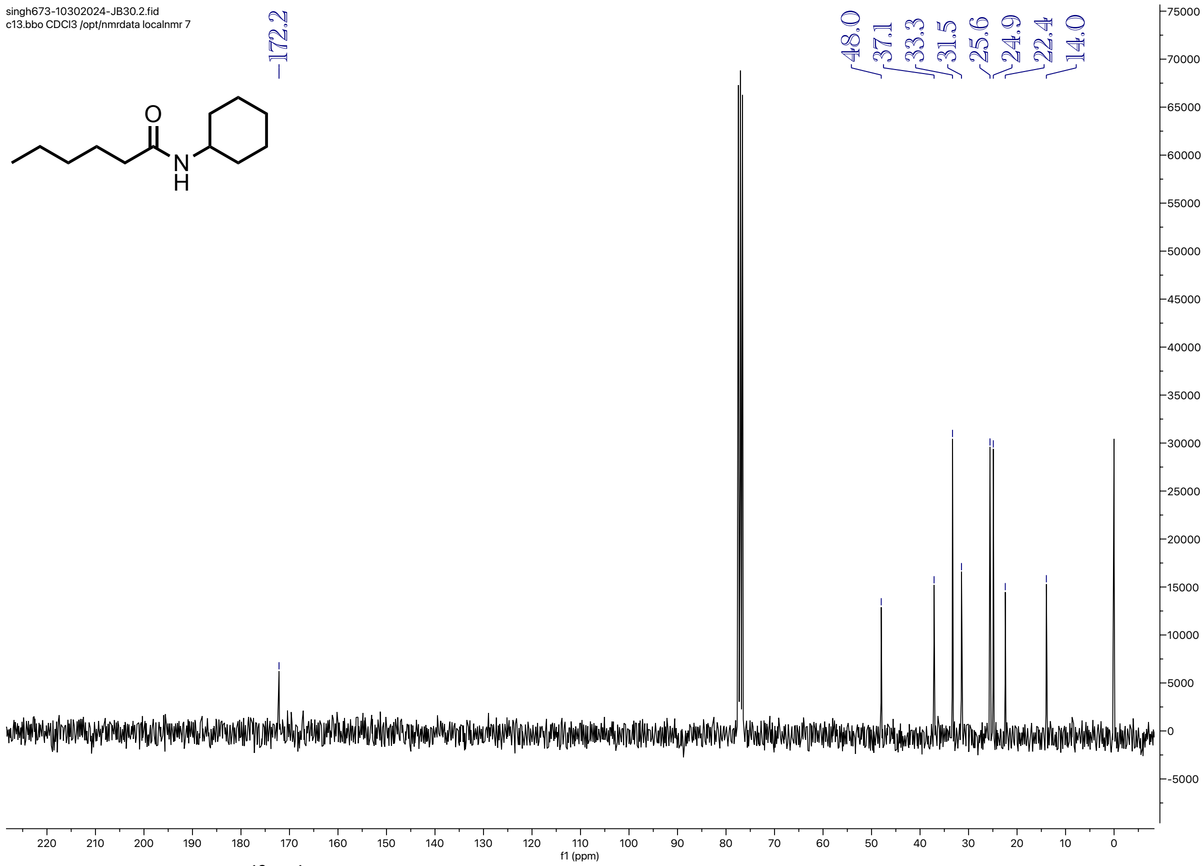

^{13}C {^{1}H} NMR (75 MHz, Chloroform-*d*) *N*-cyclohexylhexanamide (**21**)

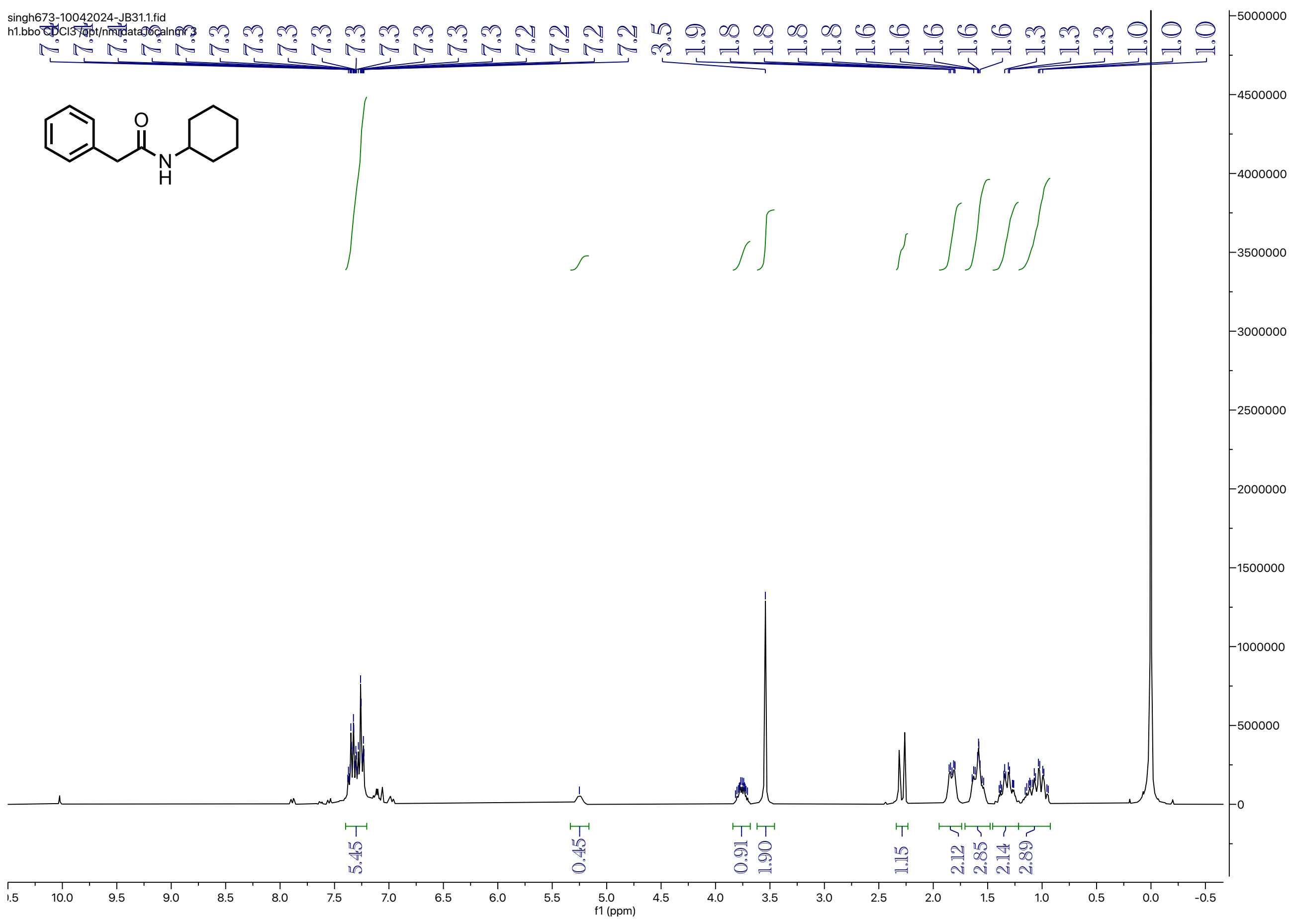

^{1}H NMR (300 MHz, Chloroform-*d*) *N*-cyclohexyl-2-phenylacetamide (**22**)

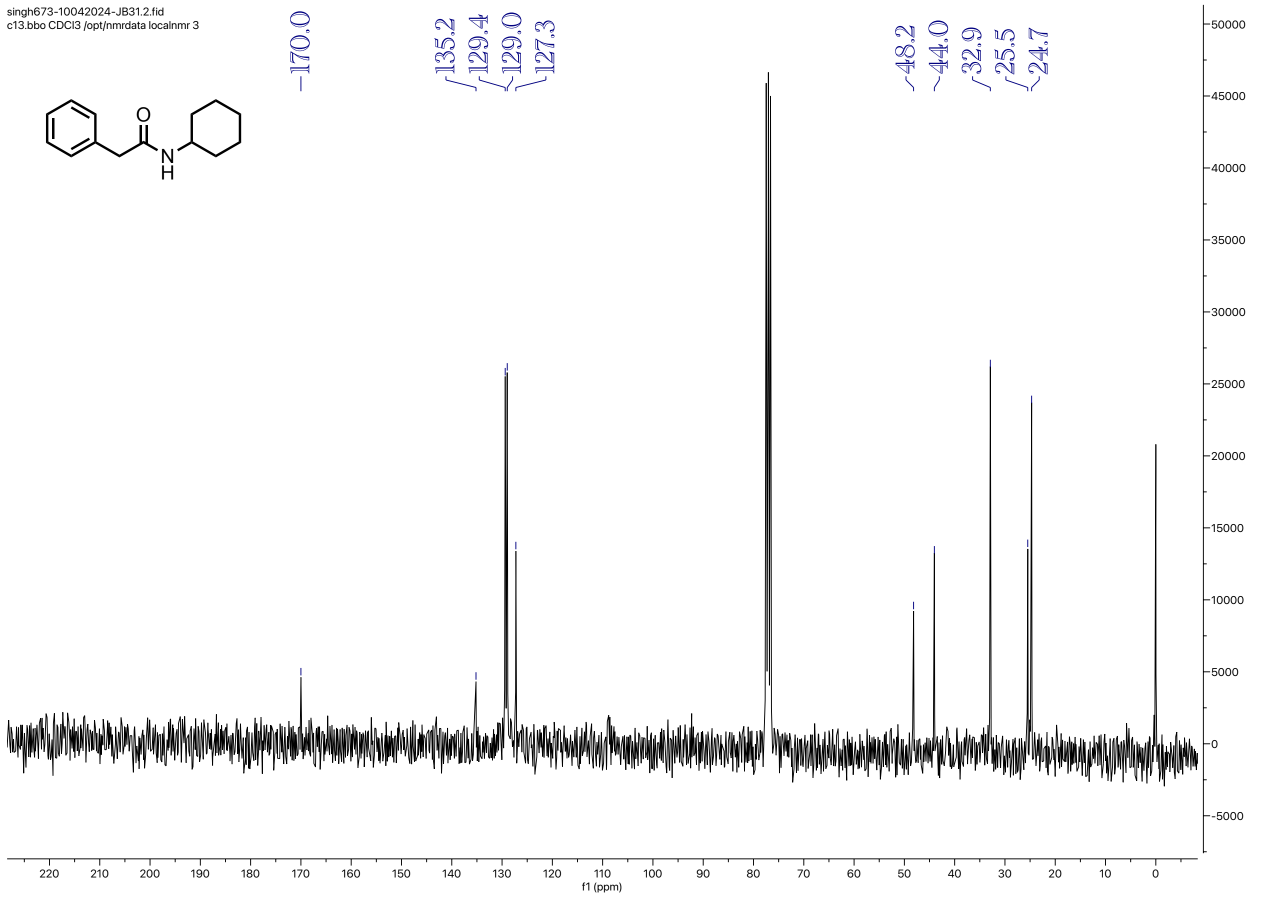

^{13}C {^{1}H} NMR (75 MHz, Chloroform-*d*) *N*-cyclohexyl-2-phenylacetamide (**22**)

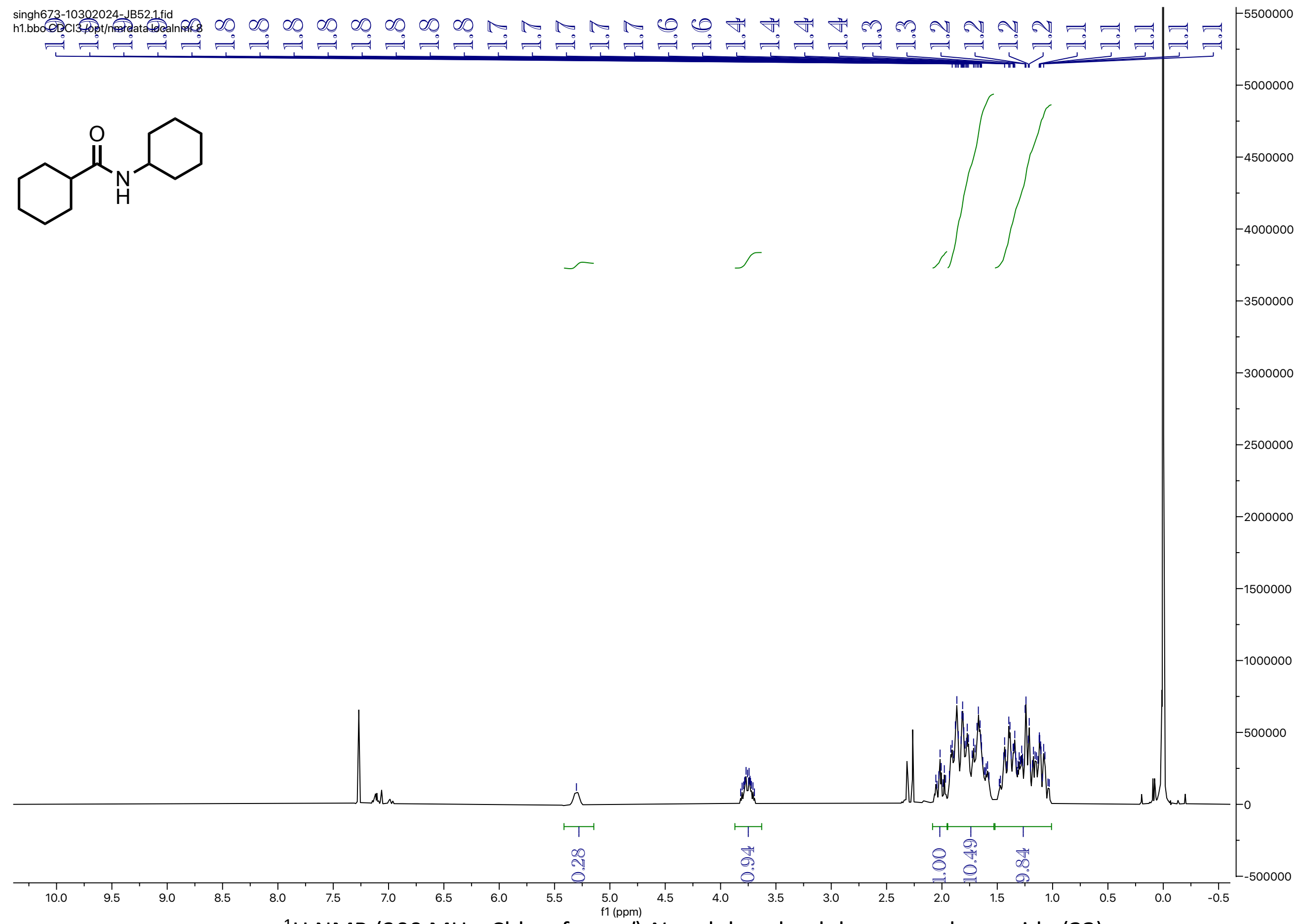

^1H NMR (300 MHz, Chloroform-*d*) *N*-cyclohexylcyclohexanecarboxamide (**23**)

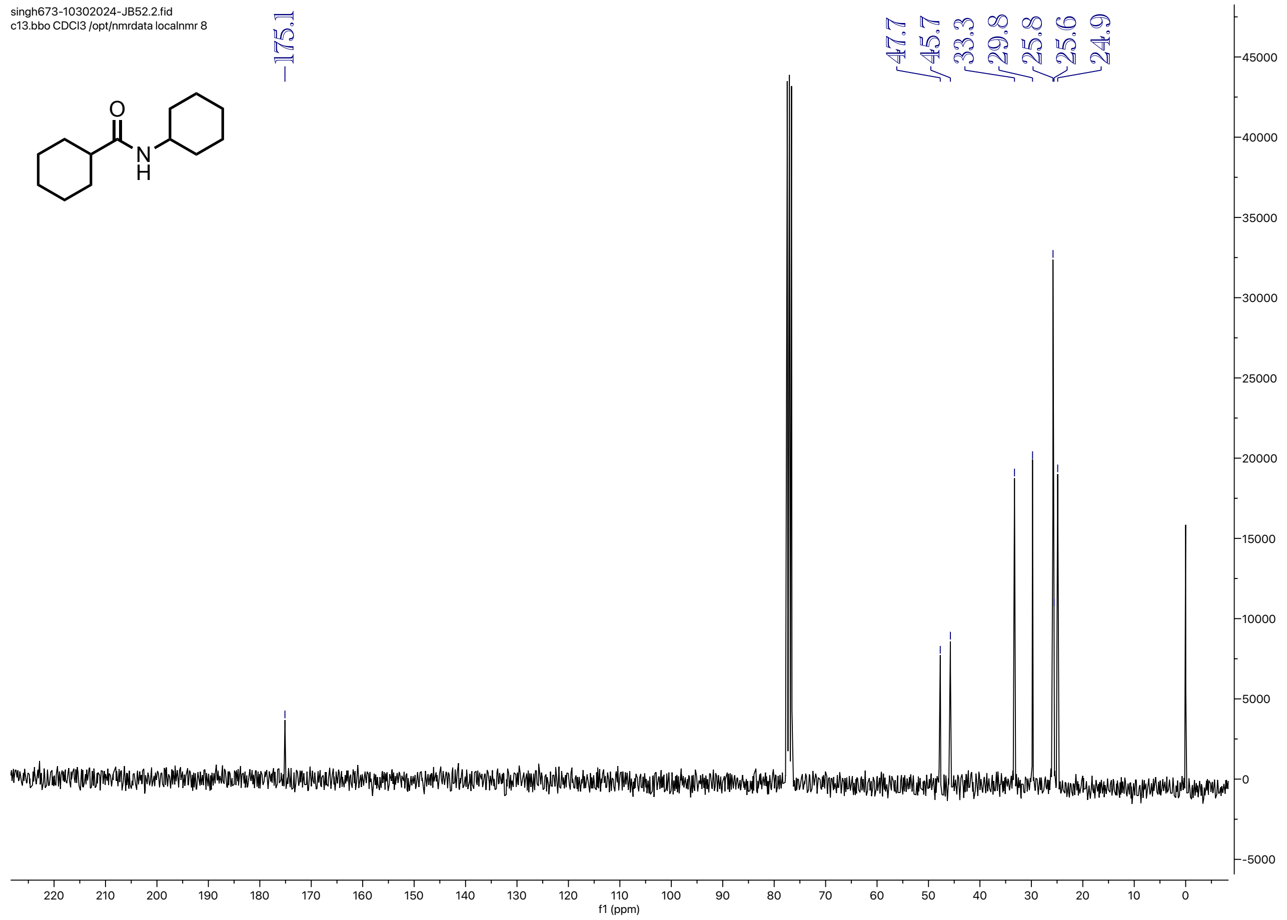

^{13}C {^1H} NMR (75 MHz, Chloroform-*d*) *N*-cyclohexylcyclohexanecarboxamide (**23**)

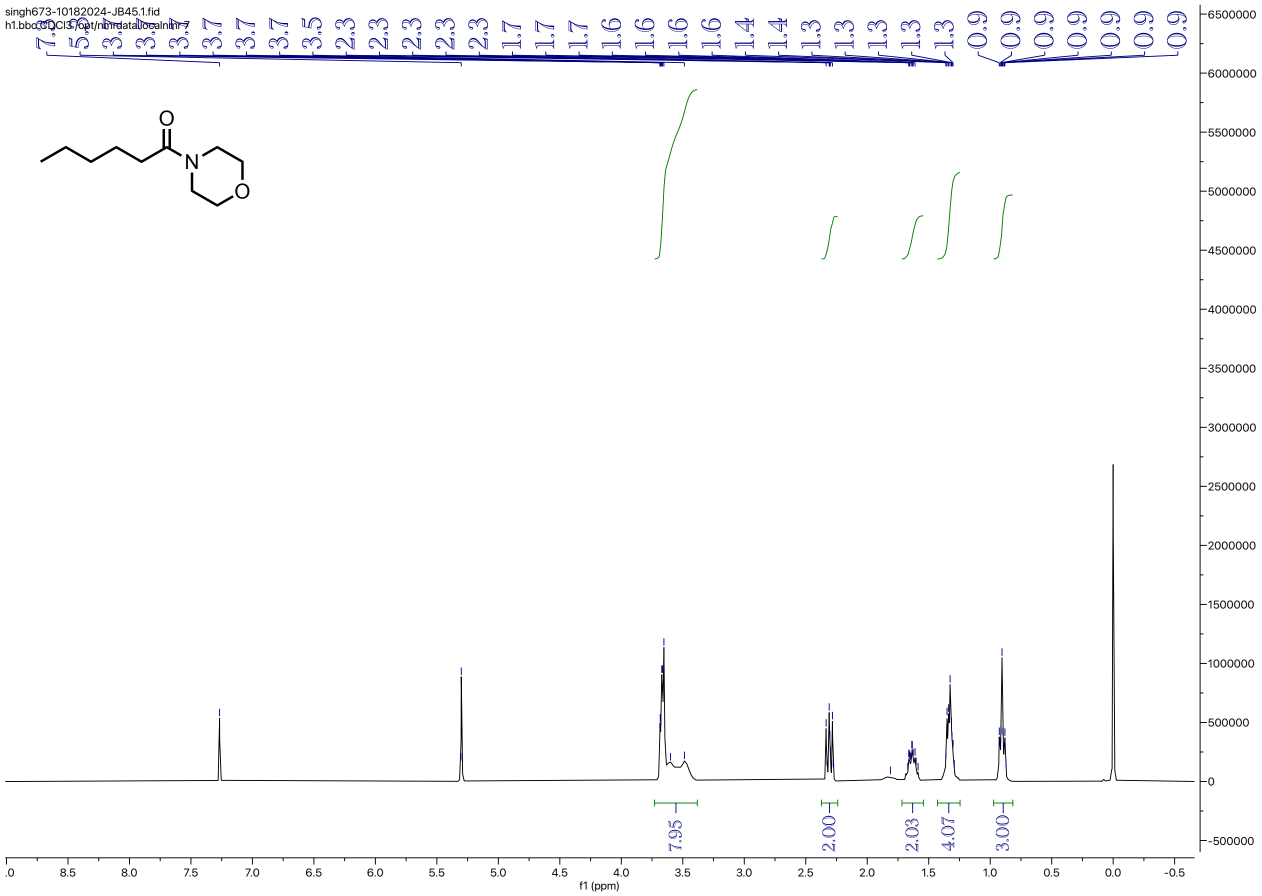

^{1}H NMR (300 MHz, Chloroform-*d*) 1-morpholinohexan-1-one (**24**)

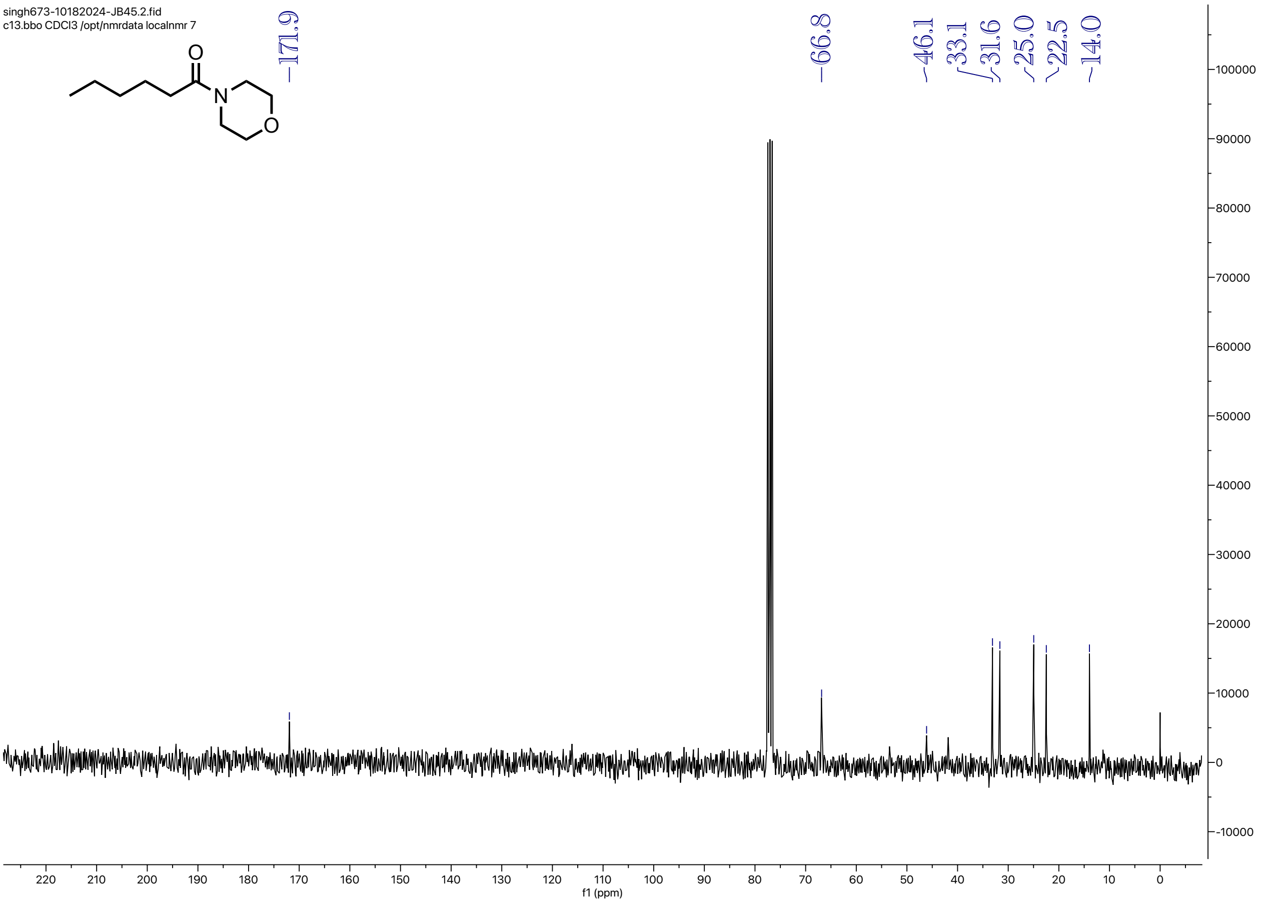

^{13}C {^{1}H} NMR (75 MHz, Chloroform-*d*) 1-morpholinohexan-1-one (**24**)

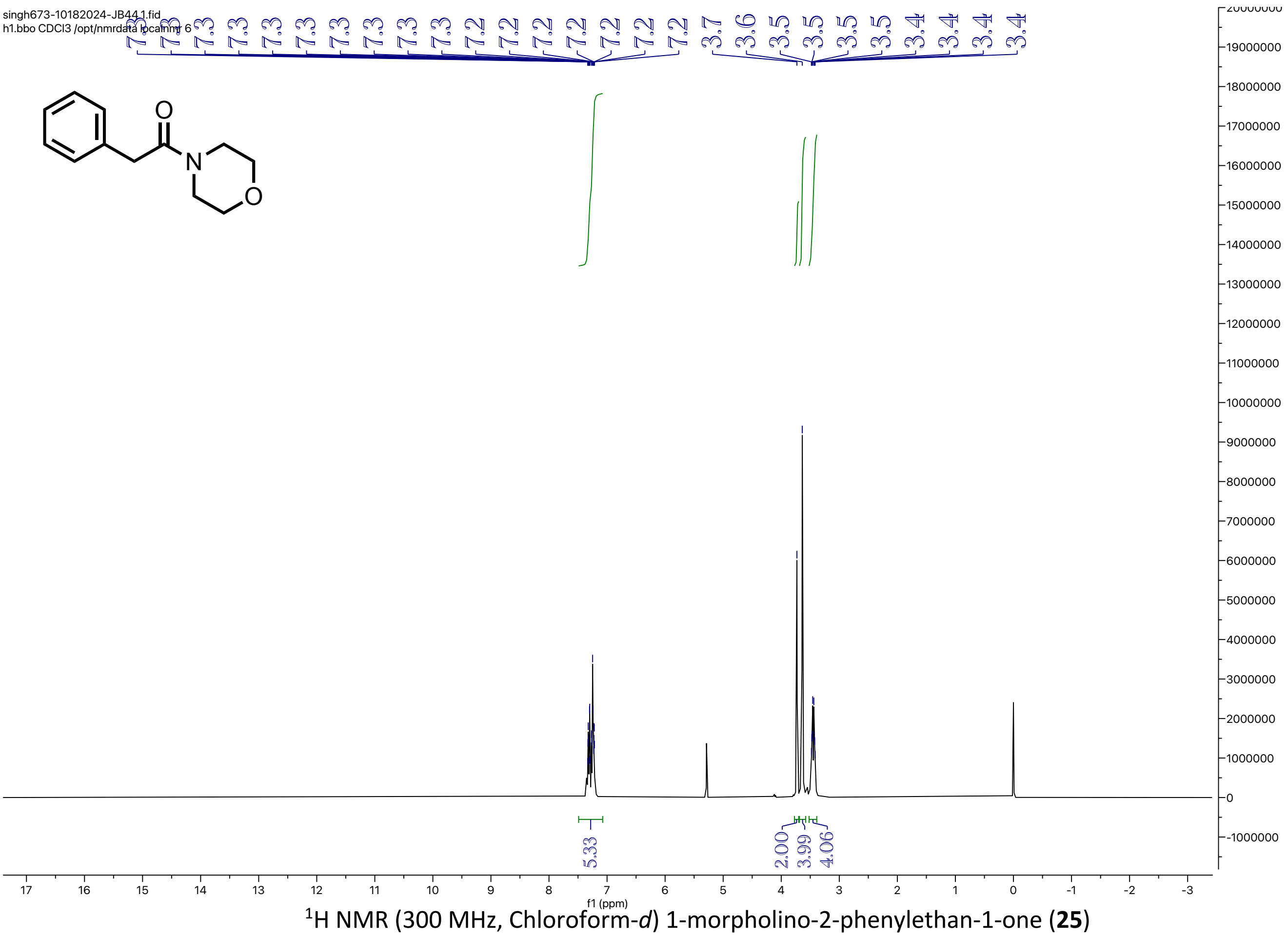

^{1}H NMR (300 MHz, Chloroform-*d*) 1-morpholino-2-phenylethan-1-one (**25**)

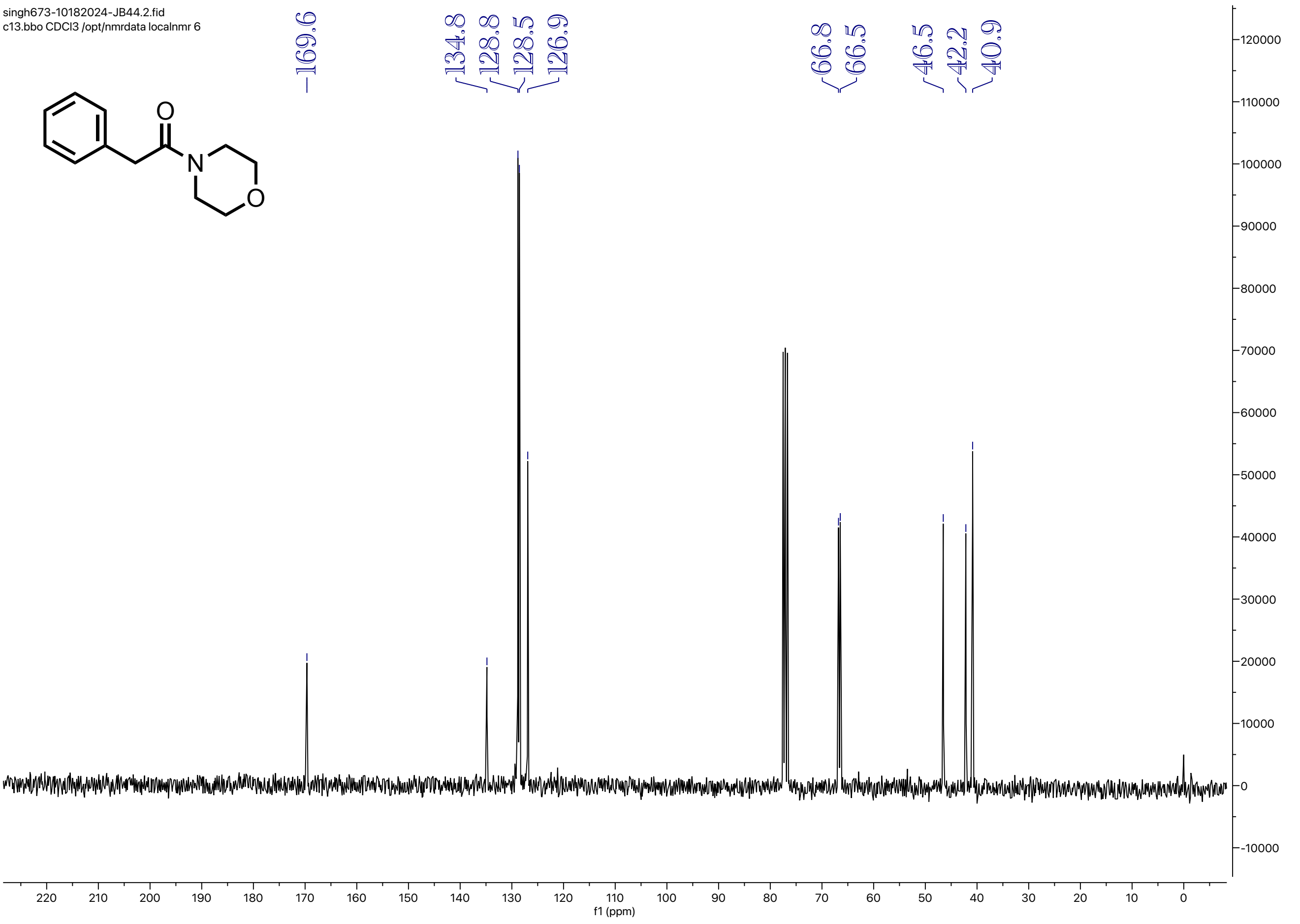

^{13}C {^{1}H} NMR (75 MHz, Chloroform-*d*) 1-morpholino-2-phenylethan-1-one (**25**)

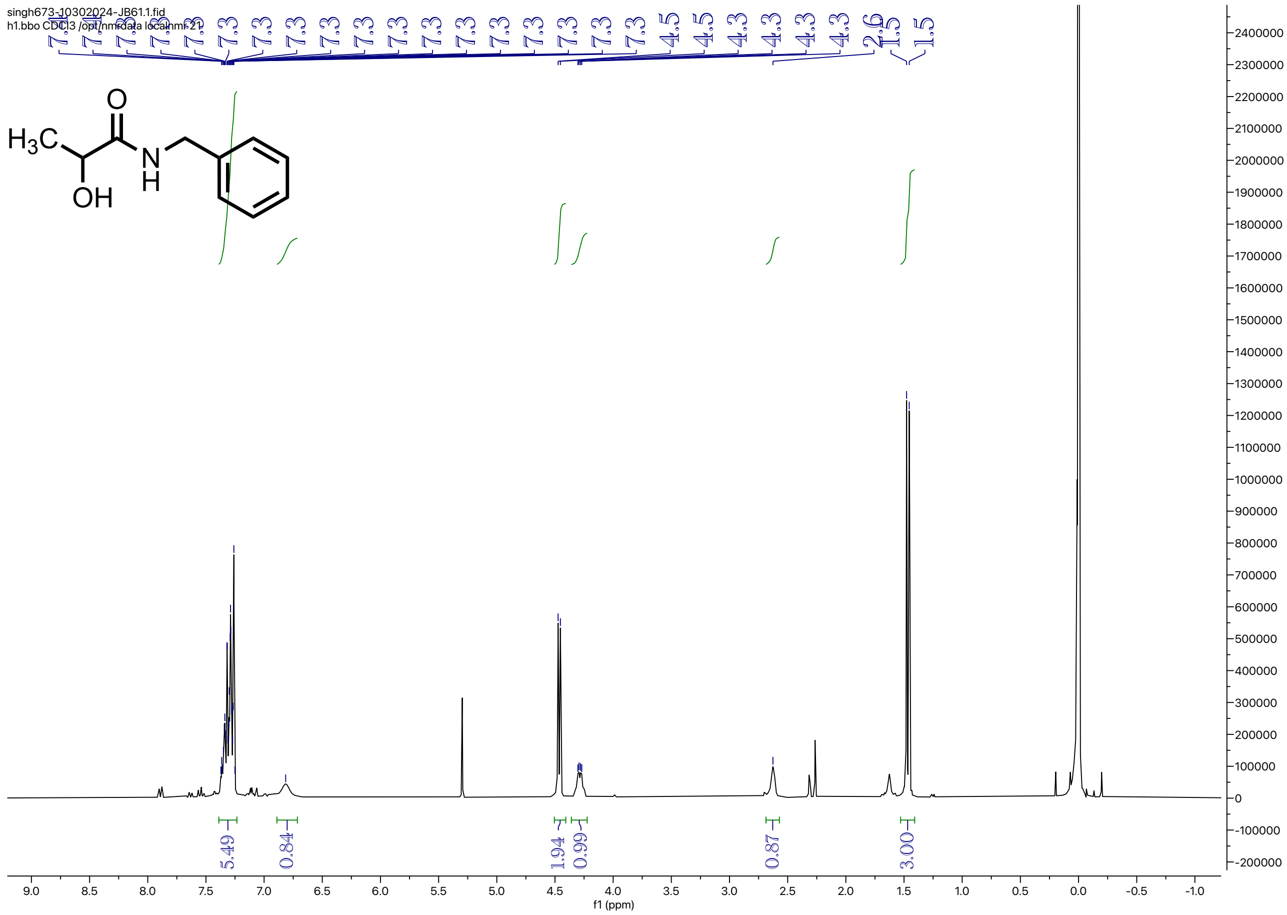

^{1}H NMR (300 MHz, Chloroform-*d*) *N*-benzyl-2-hydroxypropanamide (**26**)

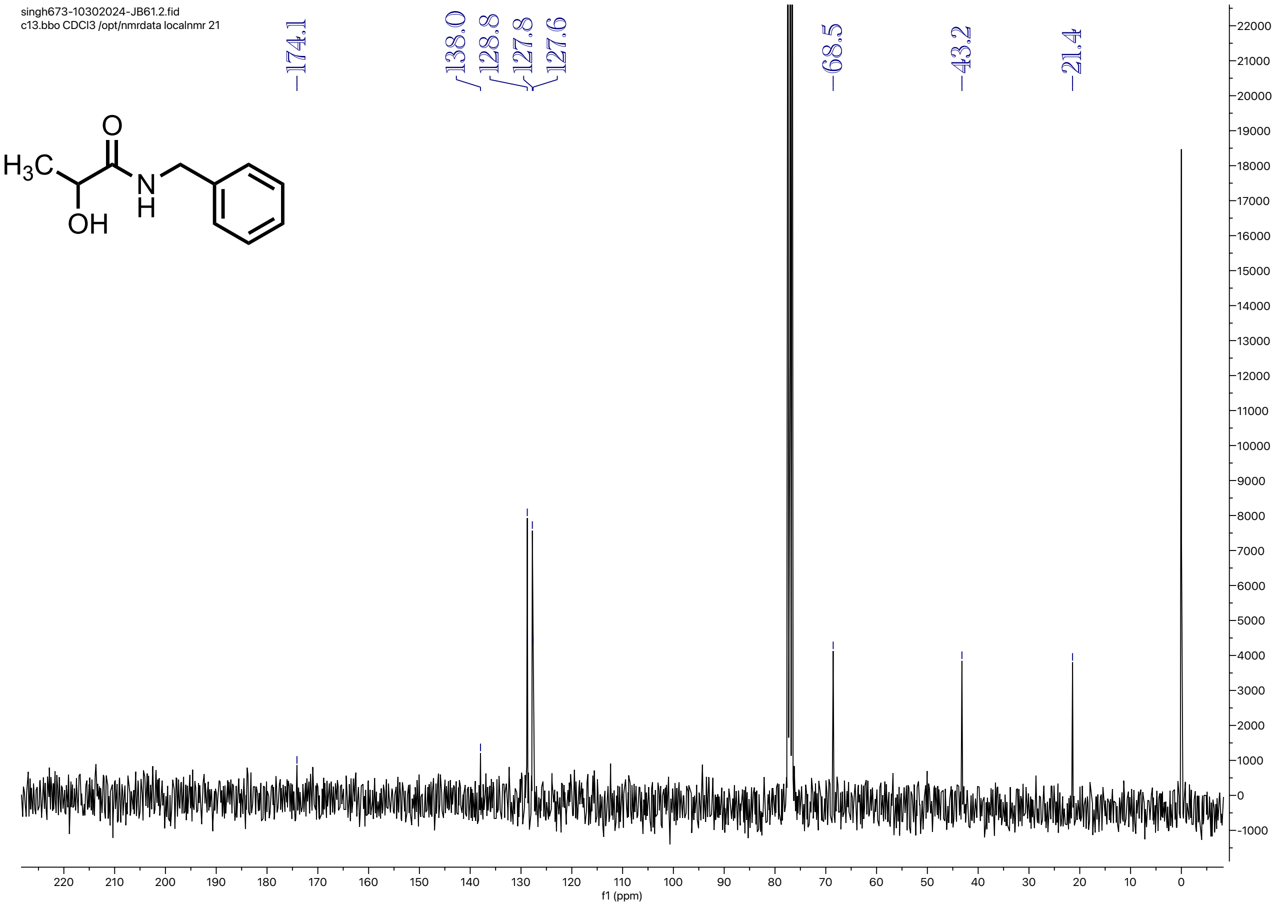

^{13}C {^{1}H} NMR (75 MHz, Chloroform-*d*) N-benzyl-2-hydroxypropanamide (**26**)

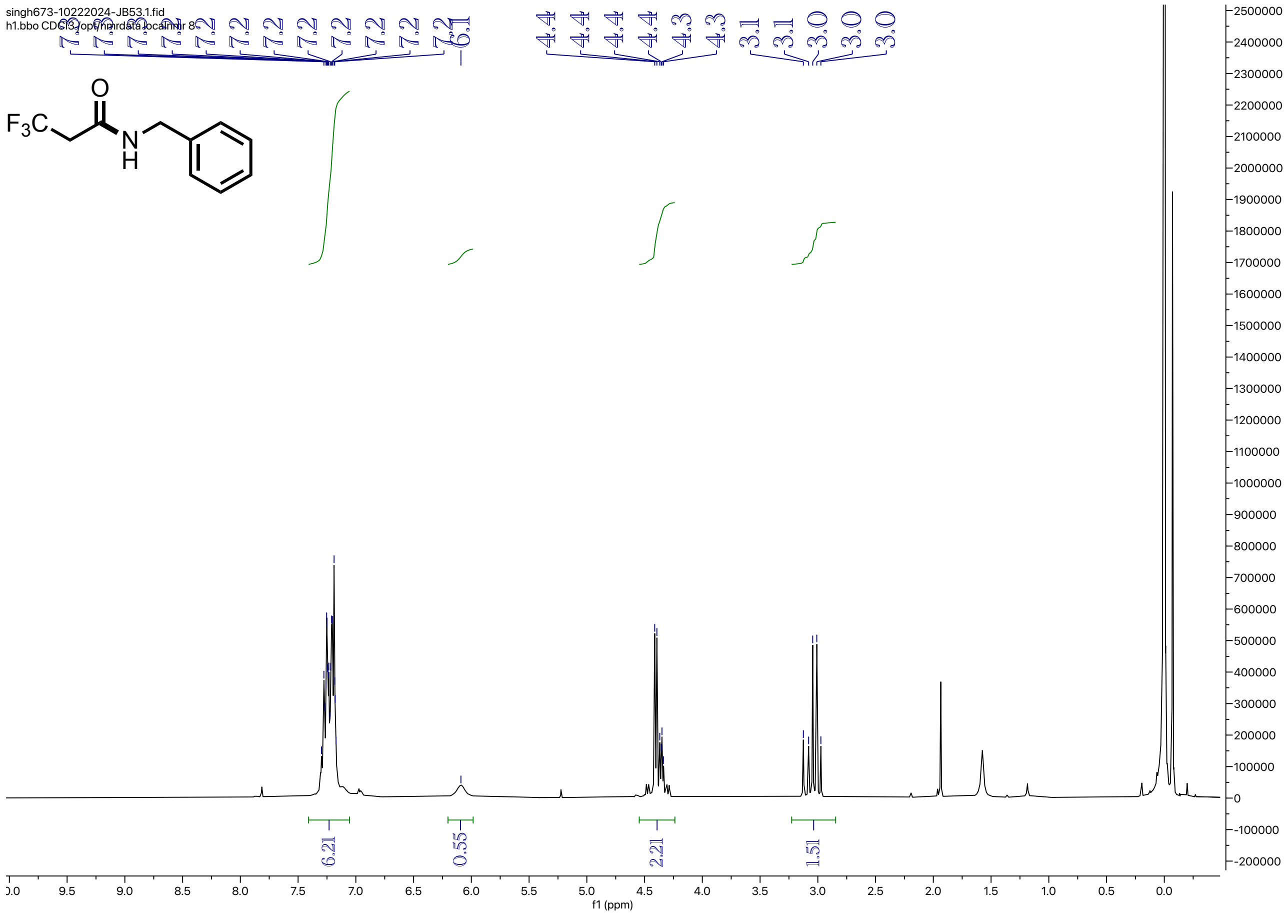

^{1}H NMR (300 MHz, Chloroform-*d*) *N*-benzyl-3,3,3-trifluoropropanamide (**27**)

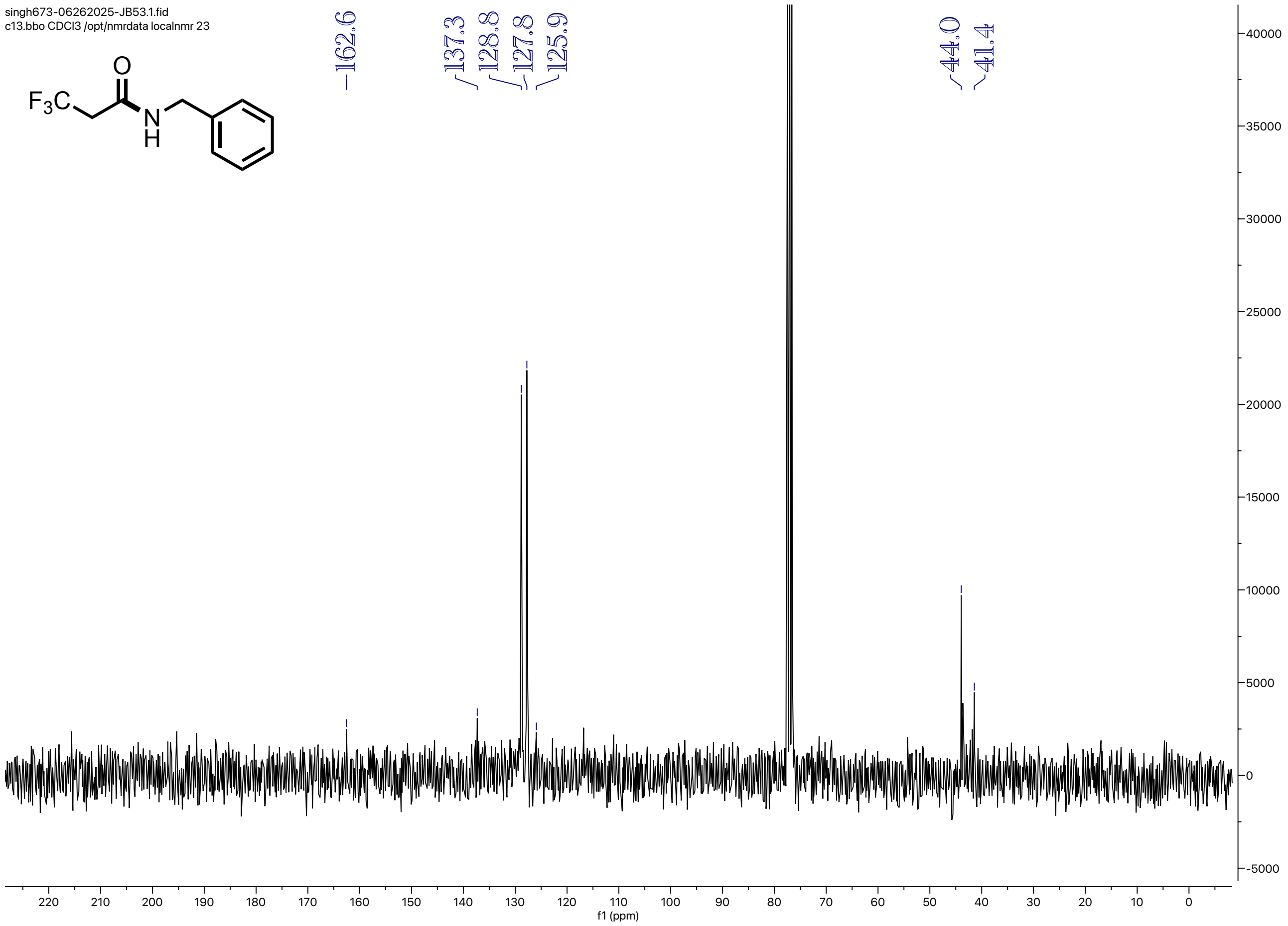

^{13}C NMR (75 MHz, Chloroform-*d*) *N*-benzyl-3,3,3-trifluoropropanamide (**27**)

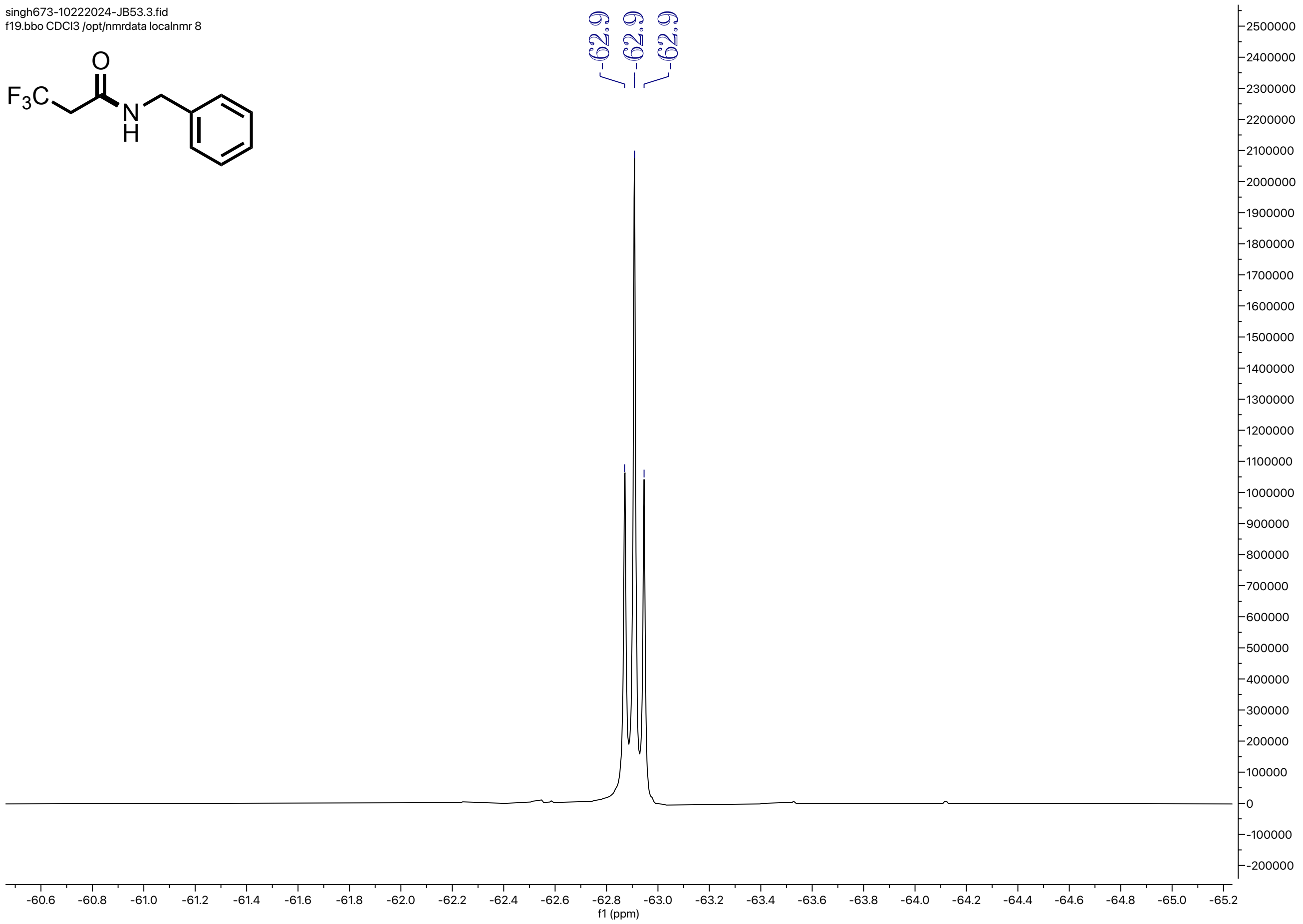

^{19}F NMR (282 MHz, Chloroform-*d*) *N*-benzyl-3,3,3-trifluoropropanamide (**27**)

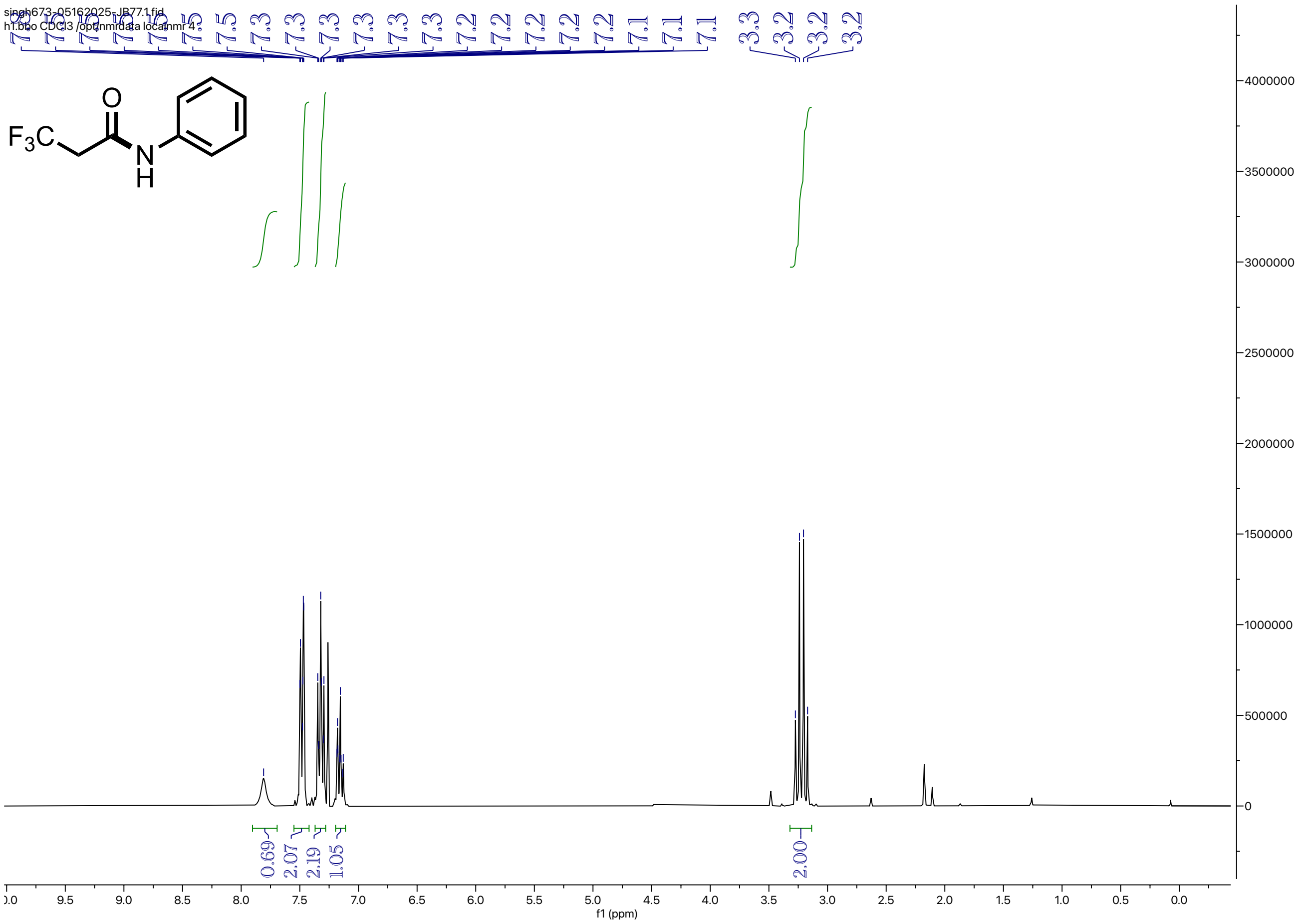

^{1}H NMR (300 MHz, Chloroform-*d*) *N*-phenyl-3,3,3-trifluoropropanamide (**28**)

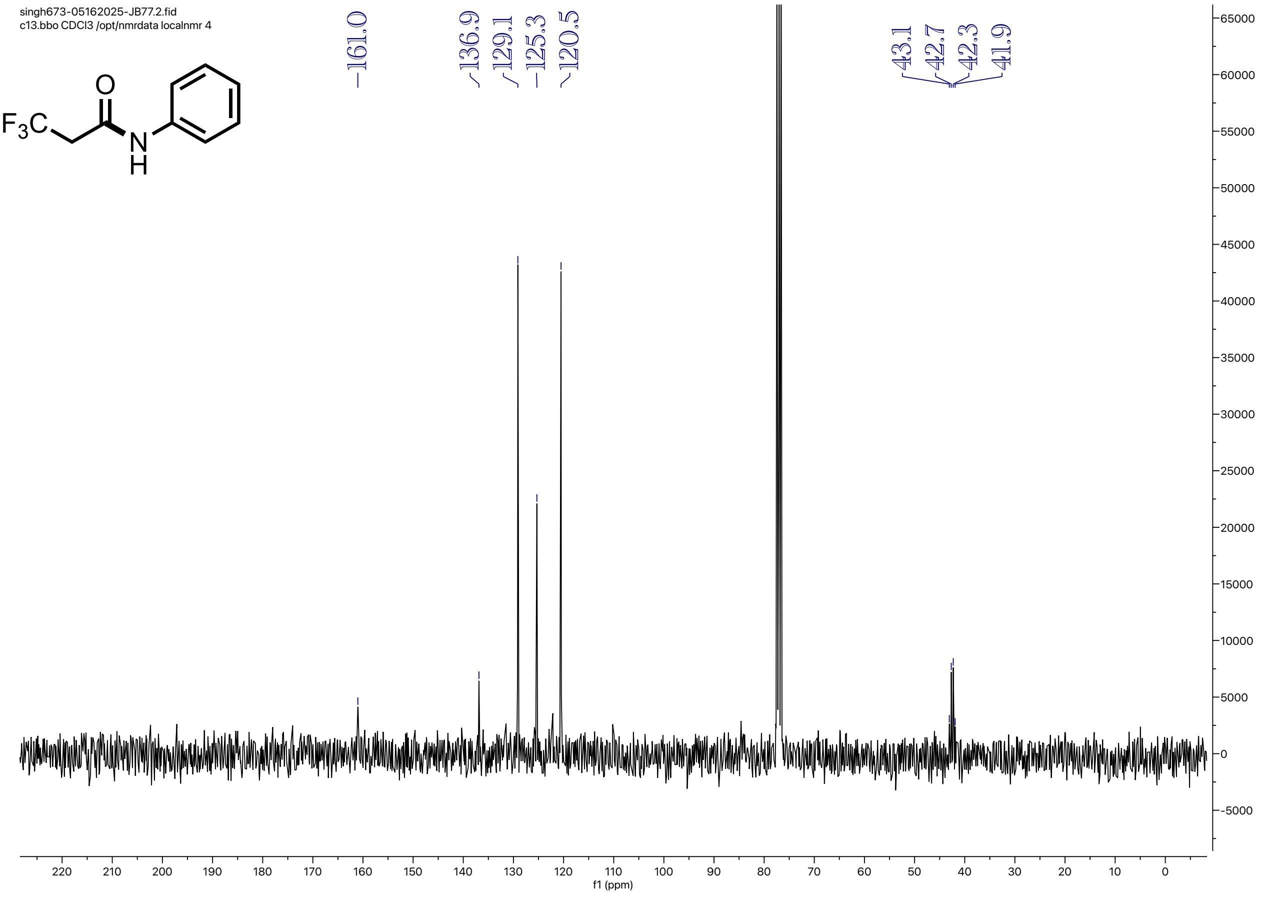

^{13}C {^{1}H} NMR (75 MHz, Chloroform-*d*) *N*-phenyl-3,3,3-trifluoropropanamide (**28**)

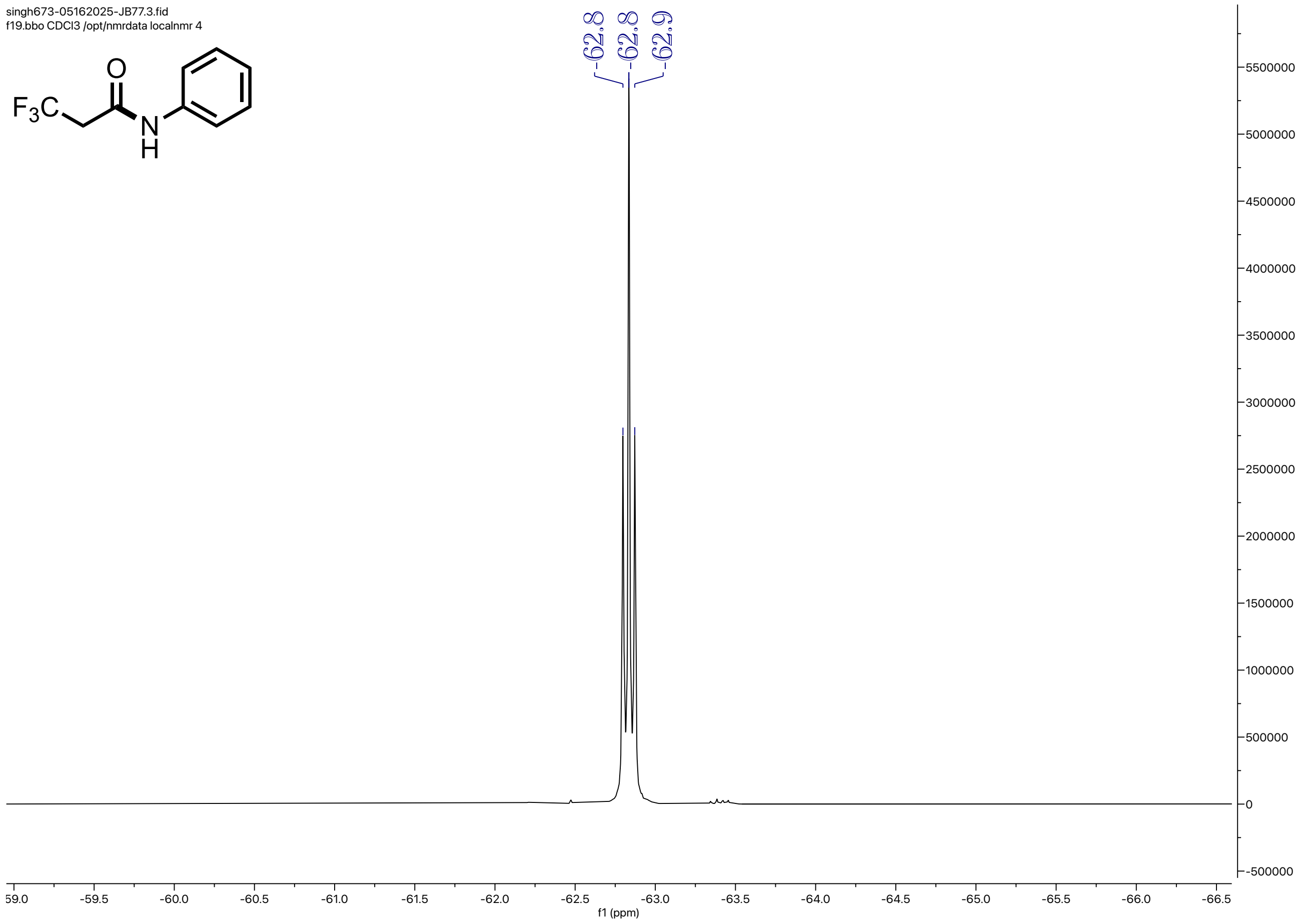

^{19}F NMR (282 MHz, Chloroform-*d*) *N*-benzyl-3,3,3-trifluoropropanamide (**28**)

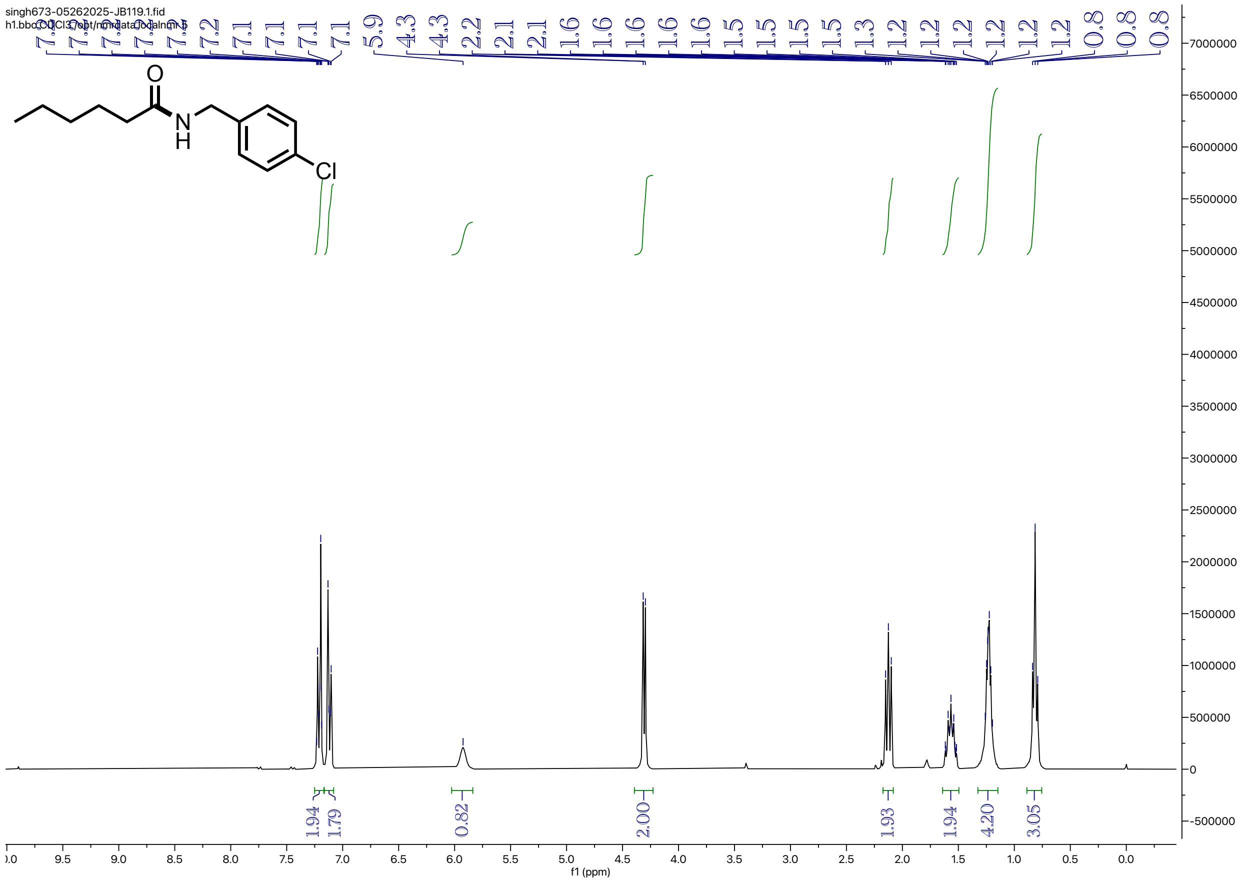

^{1}H NMR (300 MHz, Chloroform-*d*) *N*-(4-chlorobenzyl)hexanamide (**29**)

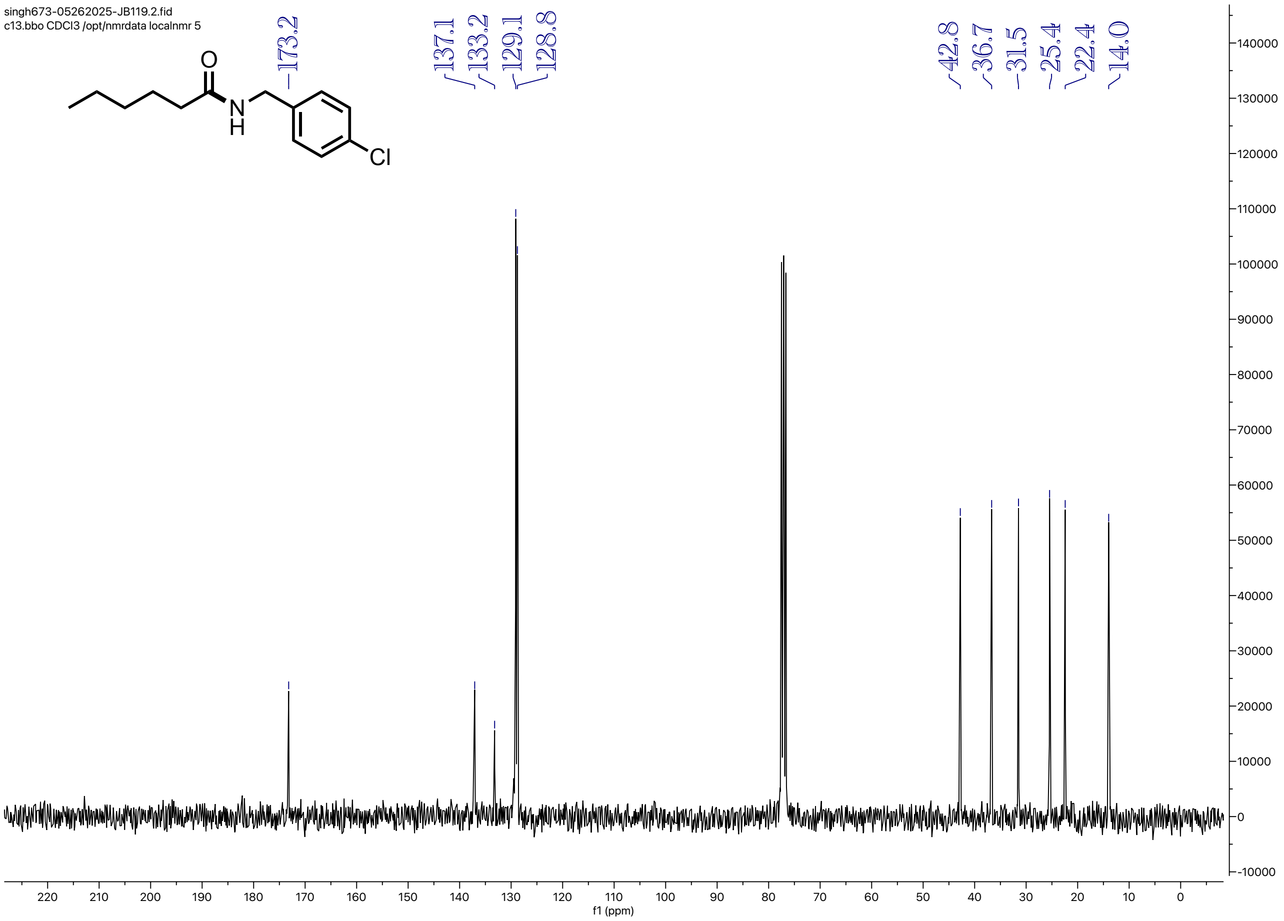

^{13}C NMR (75 MHz, Chloroform-*d*) *N*-(4-chlorobenzyl)hexanamide (**29**)

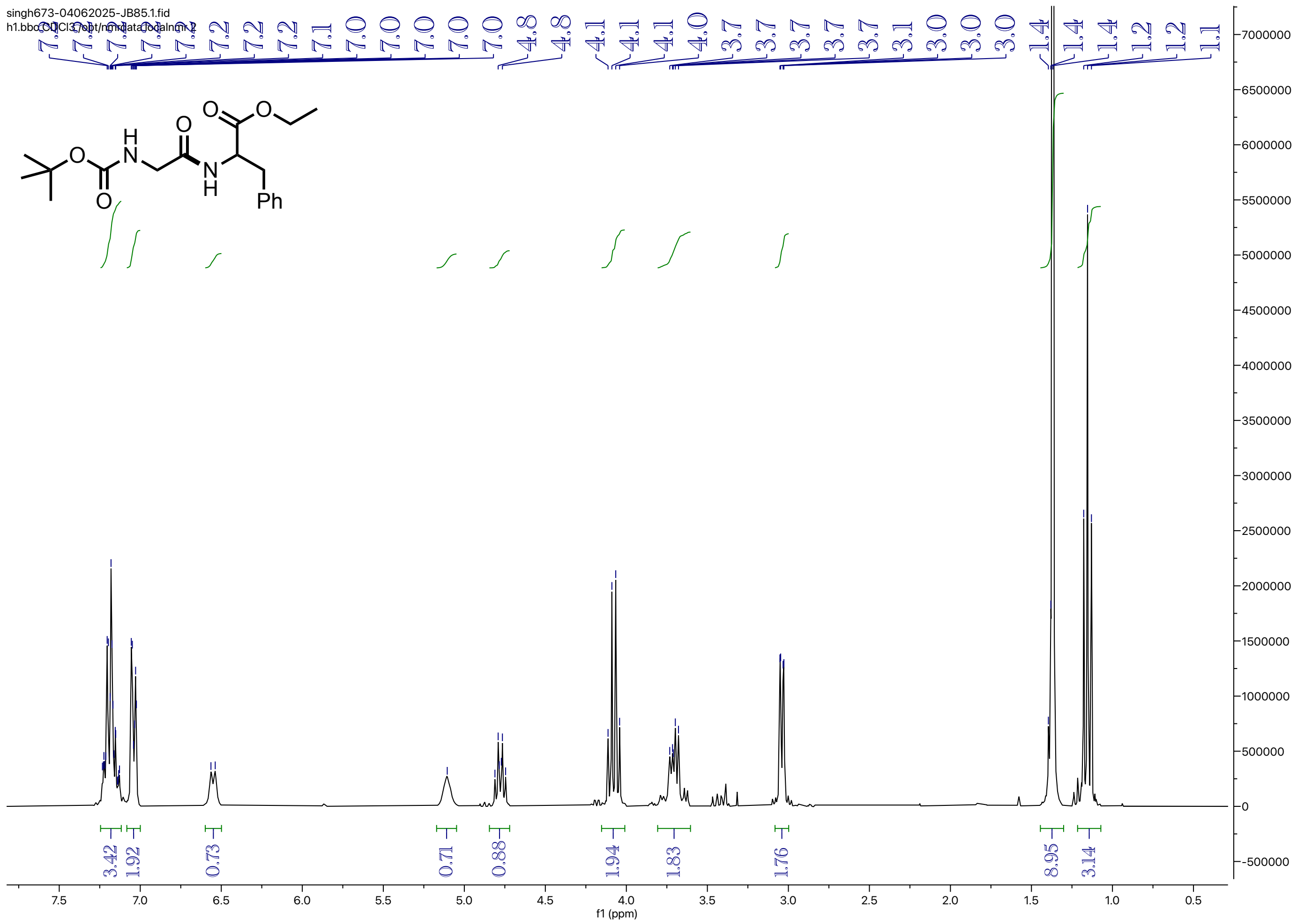

^{1}H NMR (300 MHz, Chloroform-*d*) ethyl (*tert*-butoxycarbonyl)glycylphenylalaninate (**30**)

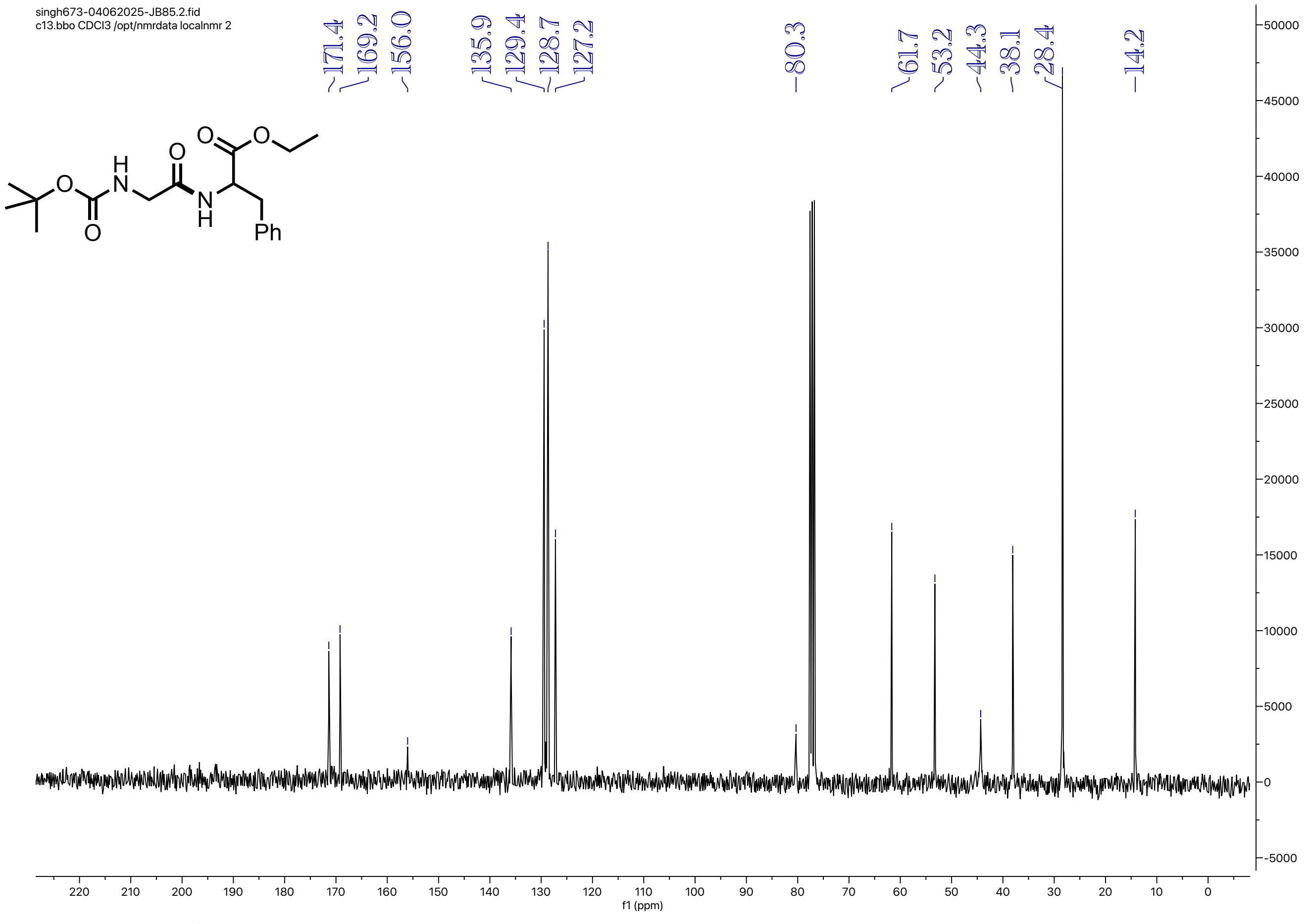

^{13}C {^{1}H} NMR (75 MHz, Chloroform-*d*) ethyl (*tert*-butoxycarbonyl)glycylphenylalaninate (**30**)

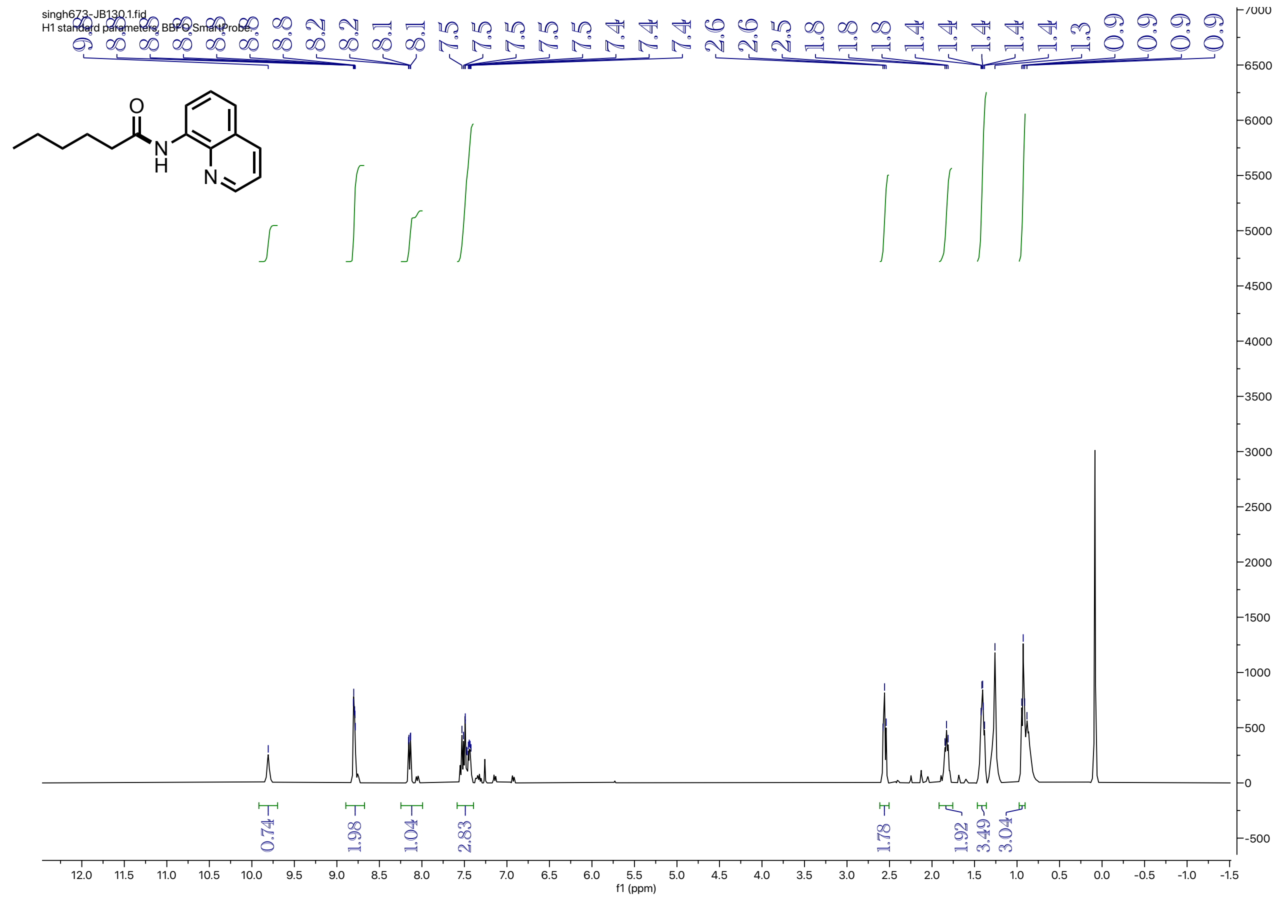

^{1}H NMR (400 MHz, Chloroform-*d*) *N*-(quinolin-8-yl)hexanamide (**31**)

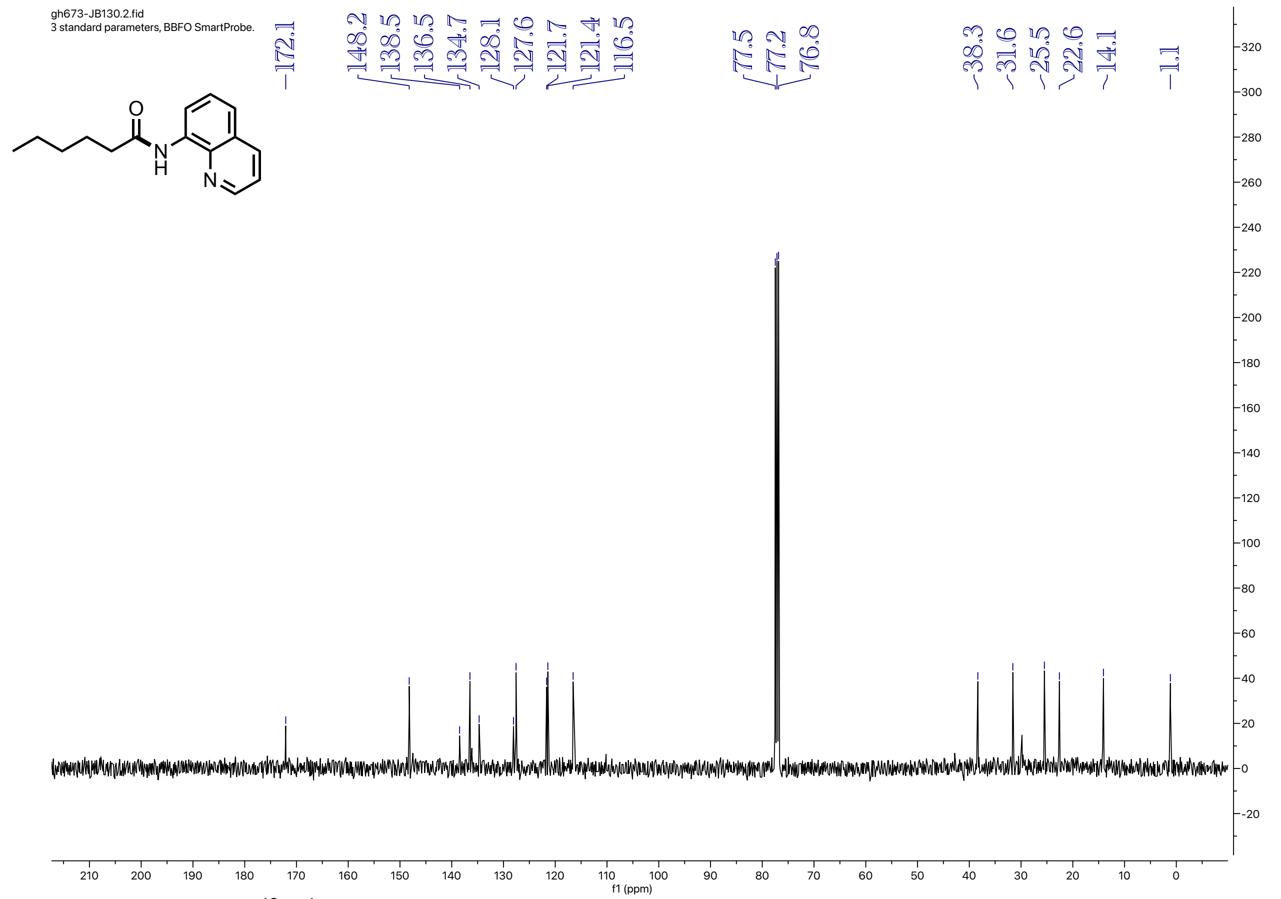

^{13}C {^{1}H} NMR (101 MHz, Chloroform-*d*) *N*-(quinolin-8-yl)hexanamide (**31**)

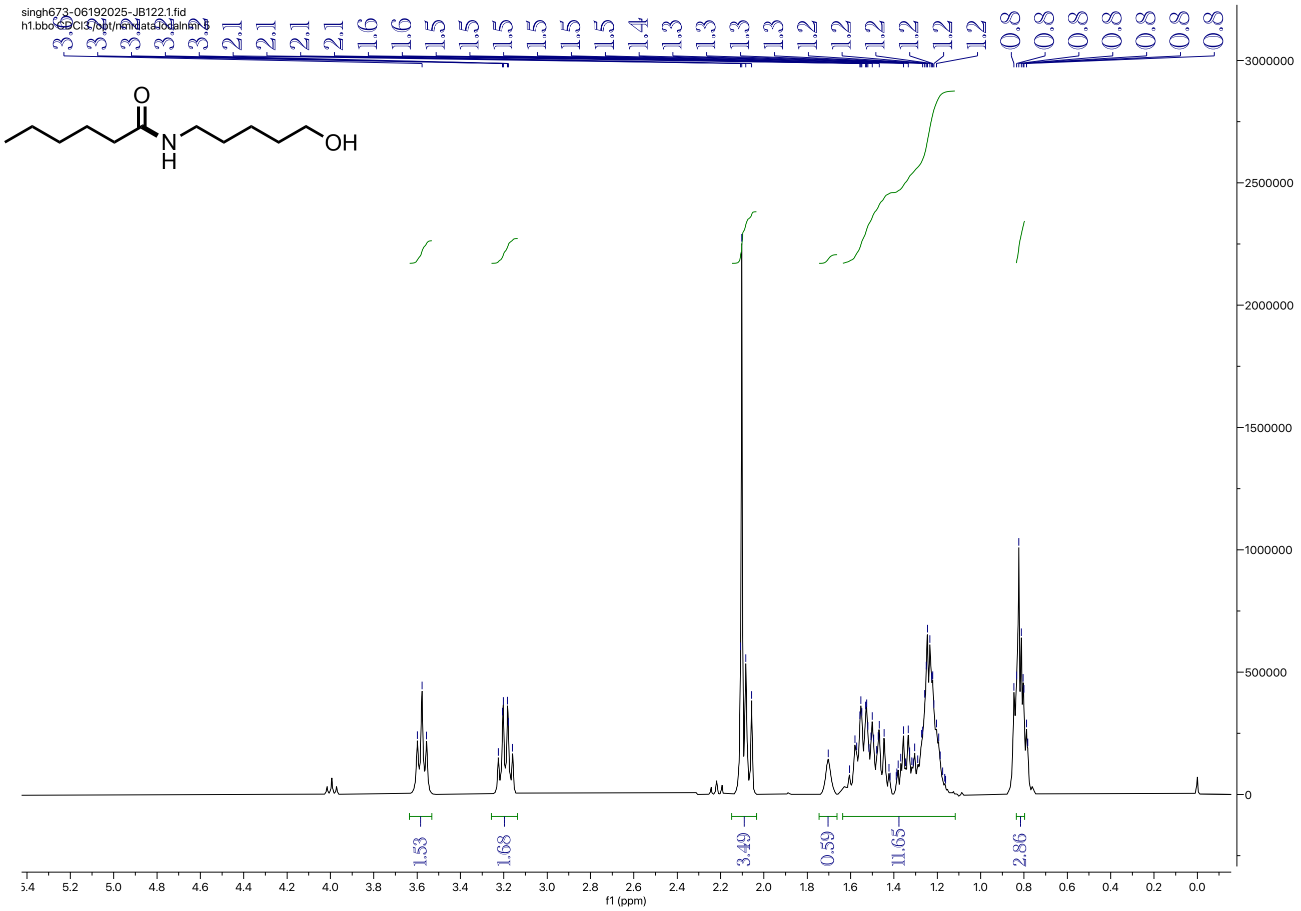

^{1}H NMR (300 MHz, Chloroform-*d*) *N-(5-hydroxypentyl)hexanamide* (**32**)

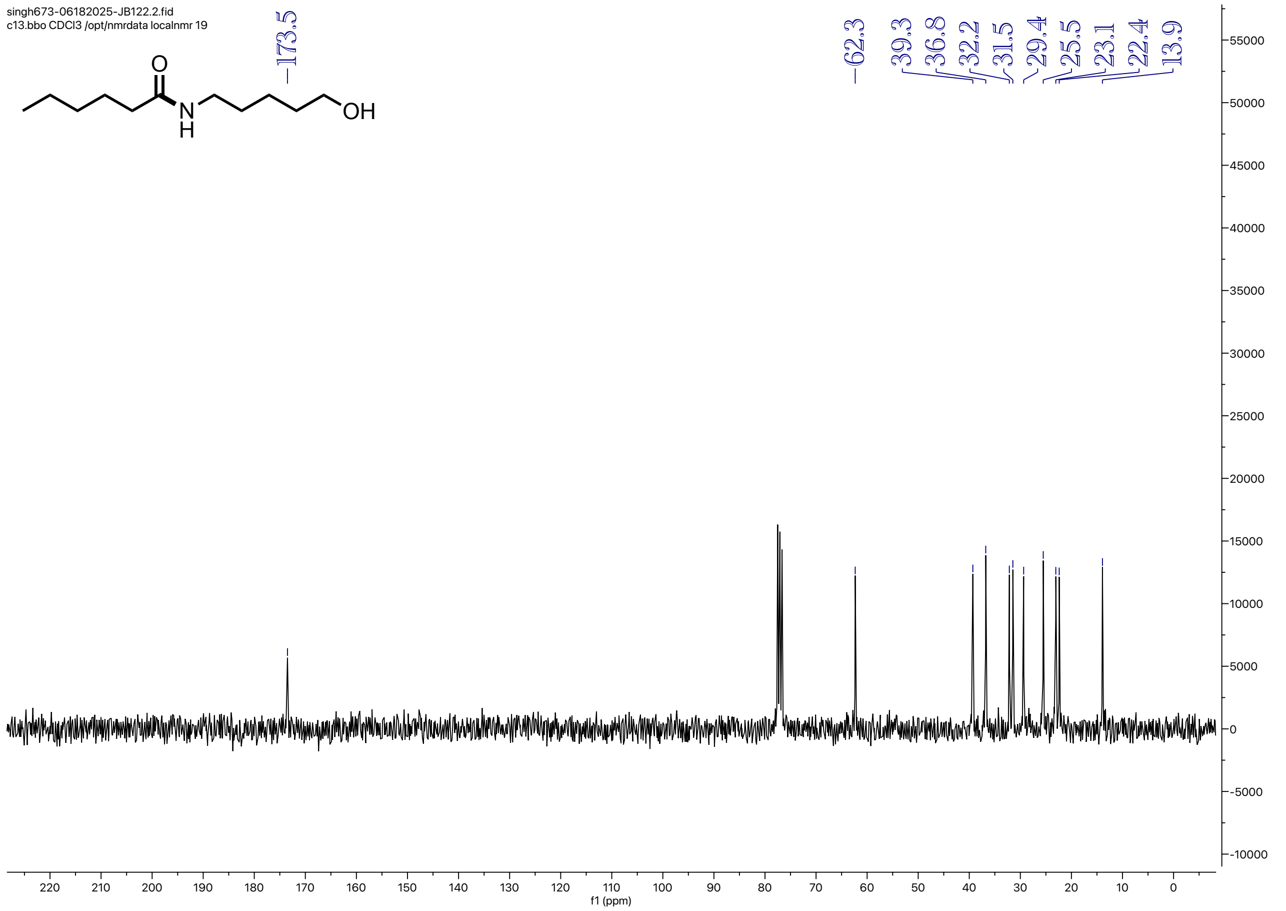

^{13}C NMR (75 MHz, Chloroform-*d*) *N-(5-hydroxypentyl)hexanamide* (**32**)

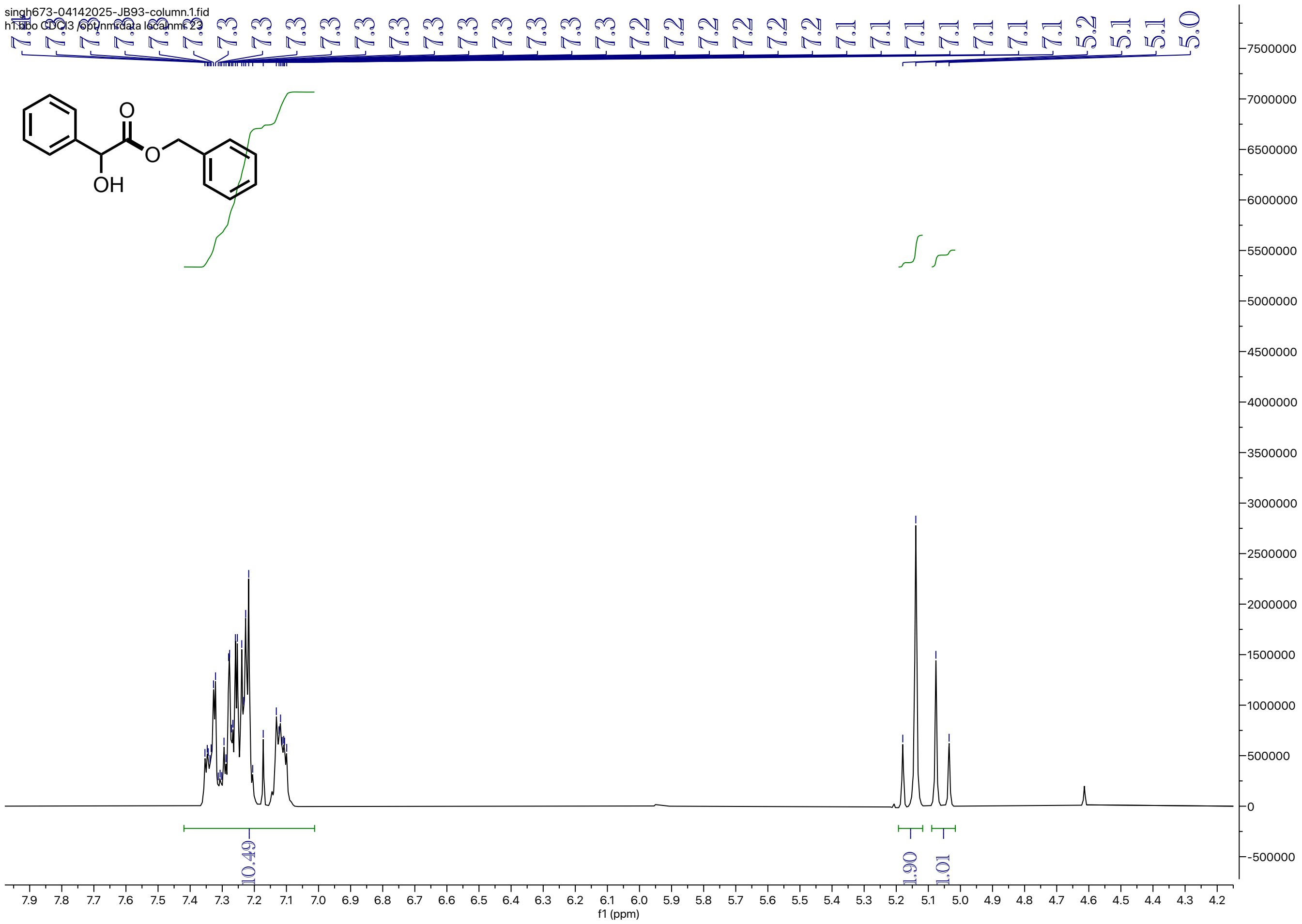

^{1}H NMR (300 MHz, Chloroform-*d*) benzyl 2-hydroxy-2-phenylacetate (**33**)

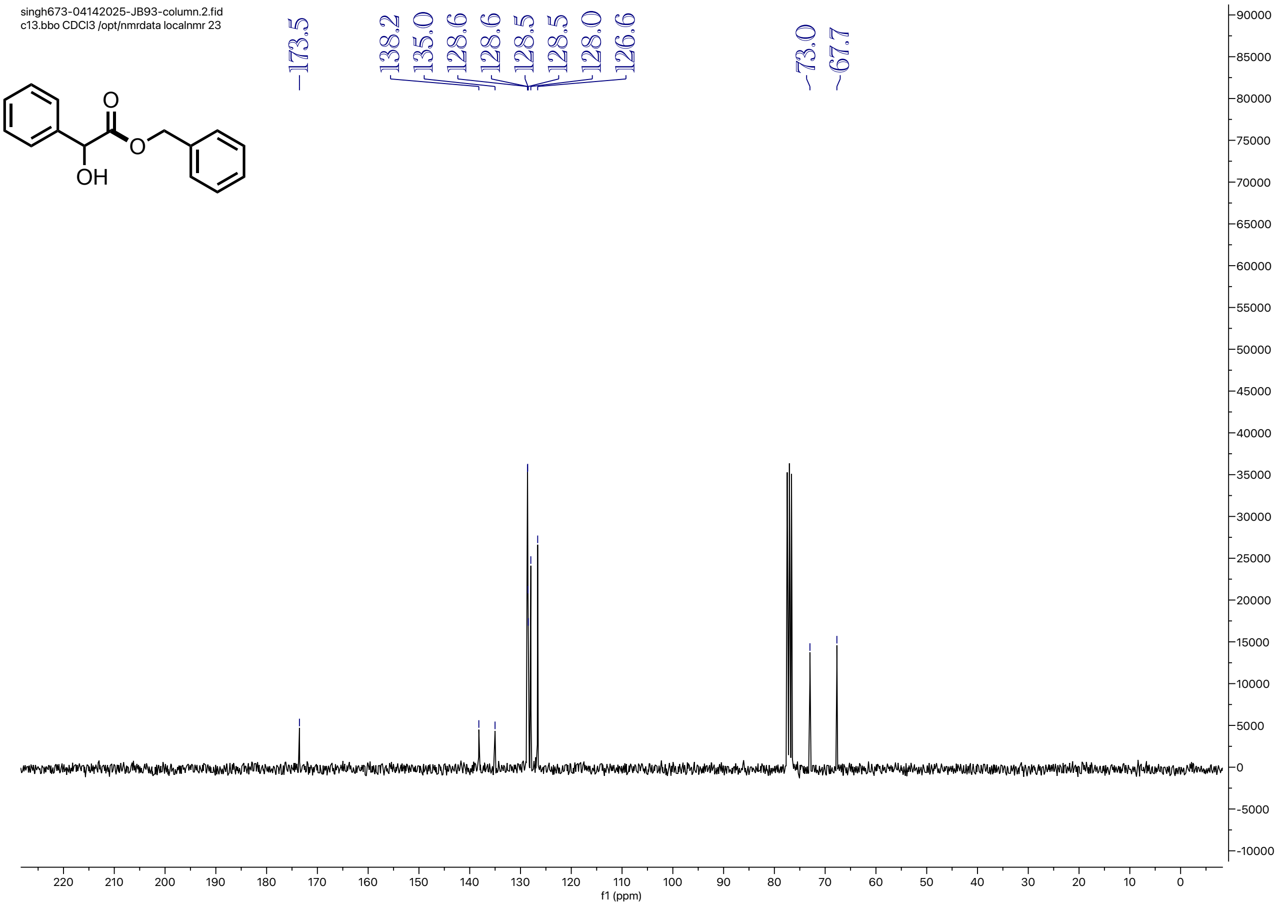

^{13}C {^{1}H} NMR (75 MHz, Chloroform-*d*) benzyl 2-hydroxy-2-phenylacetate (**33**)

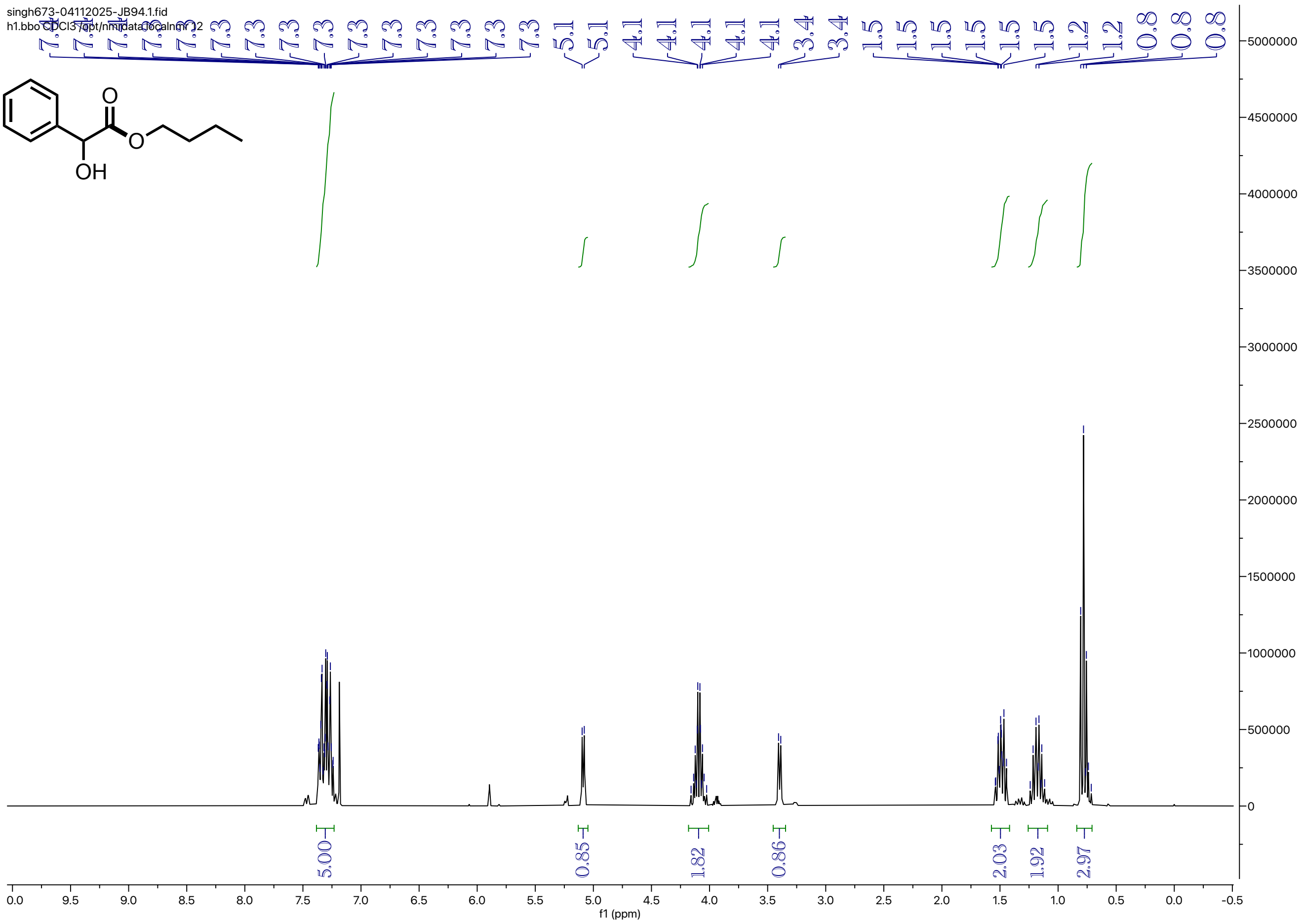

^{1}H NMR (300 MHz, Chloroform-*d*) butyl 2-hydroxy-2-phenylacetate (**34**)

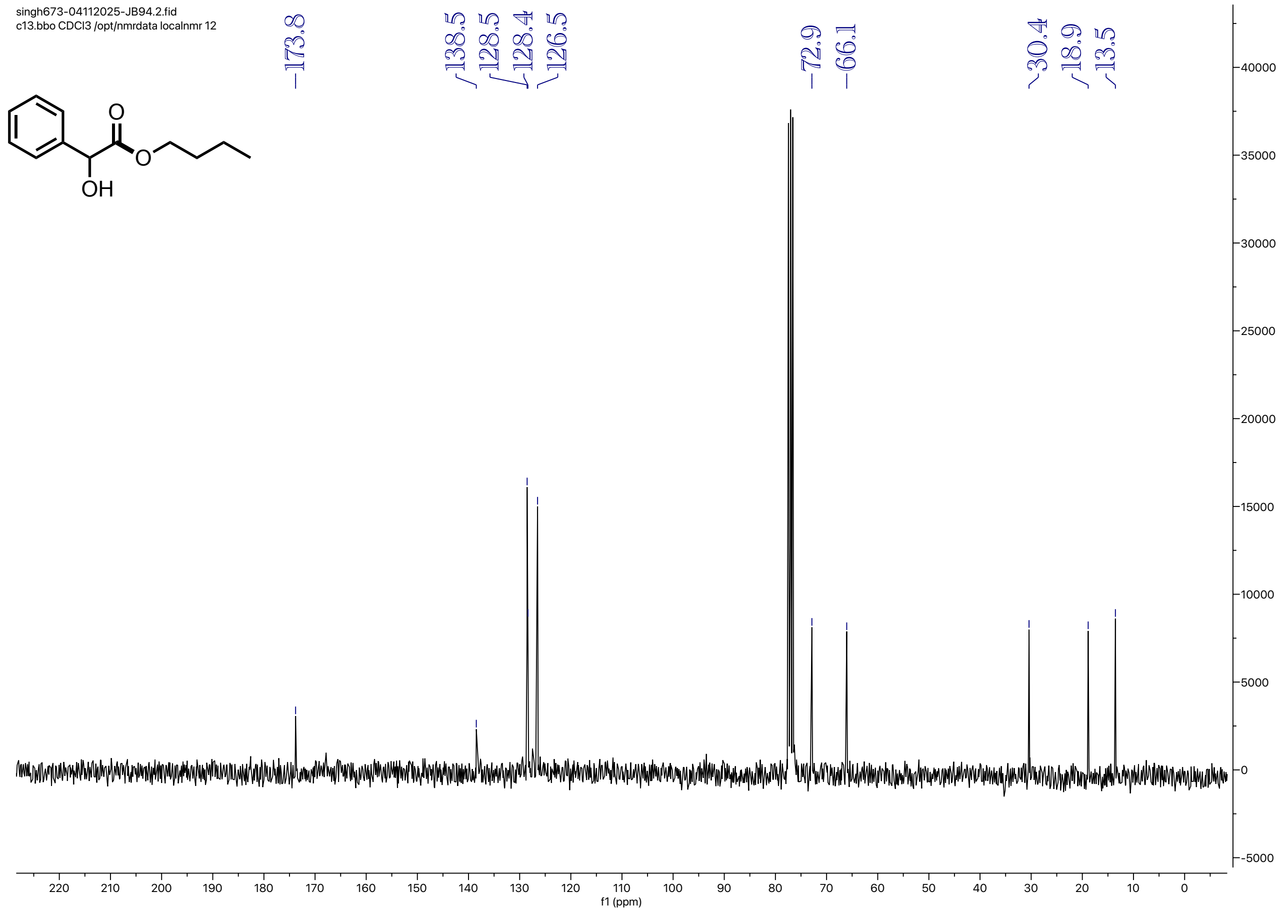

^{13}C {^{1}H} NMR (75 MHz, Chloroform-*d*) benzyl 2-hydroxy-2-phenylacetate (**34**)

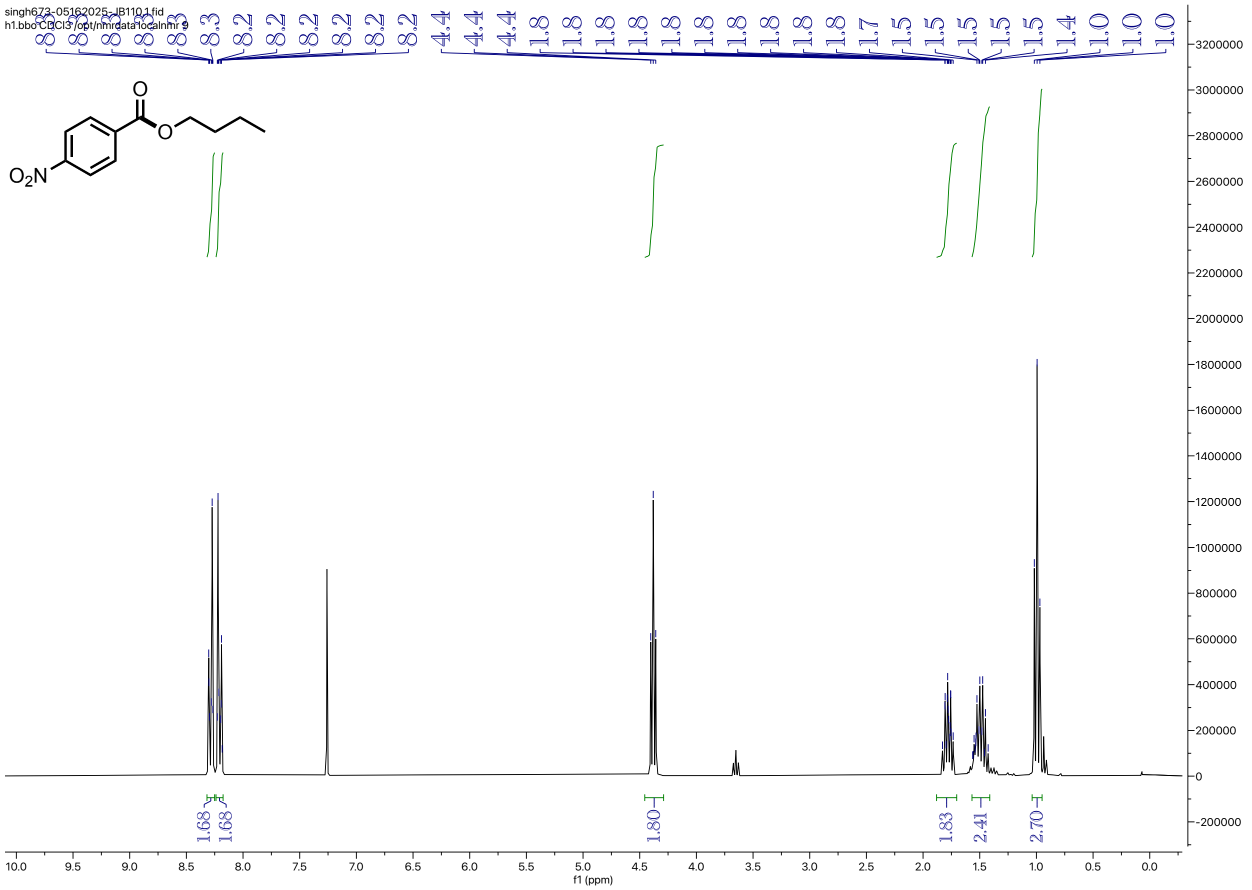

^{1}H NMR (300 MHz, Chloroform-*d*) butyl 4-nitrobenzoate (**35**)

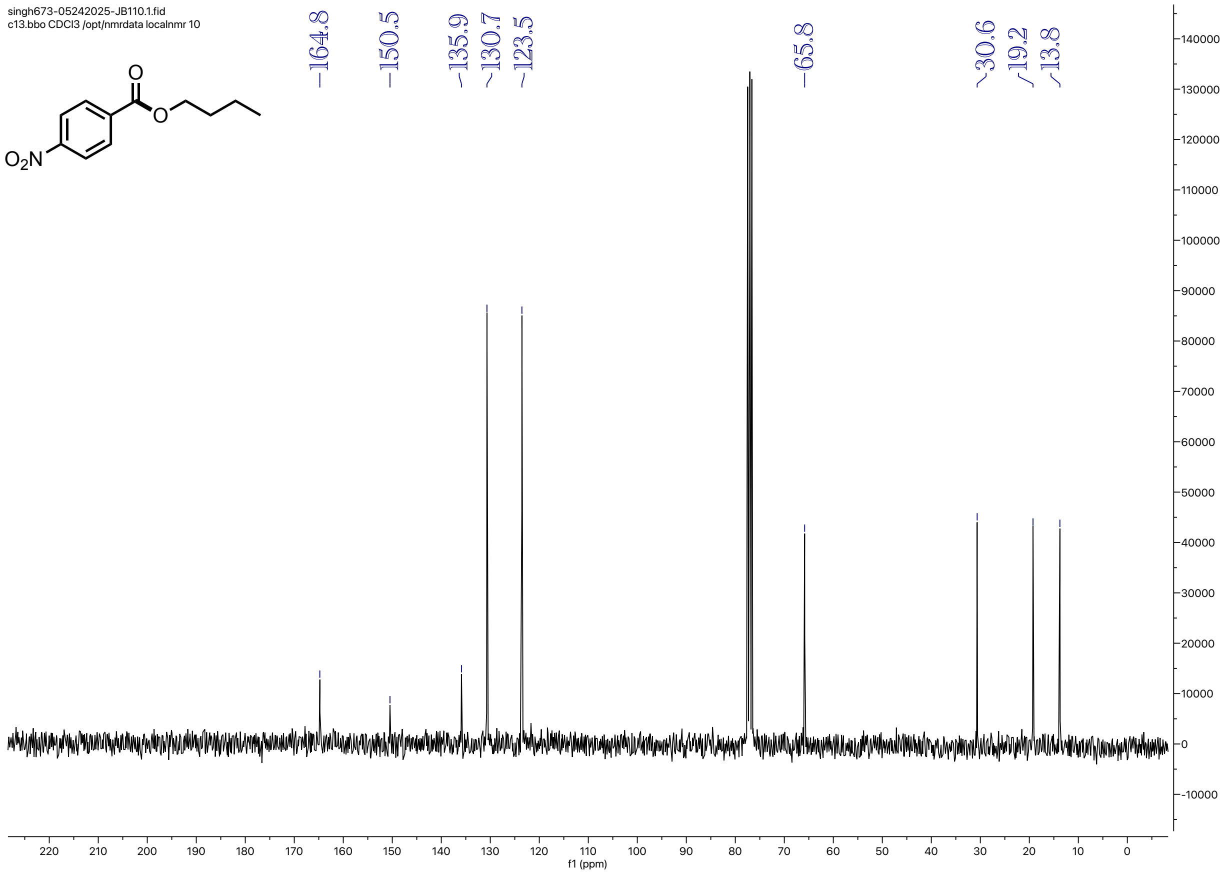

^{13}C NMR (75 MHz, Chloroform-*d*) butyl 4-nitrobenzoate (**35**)

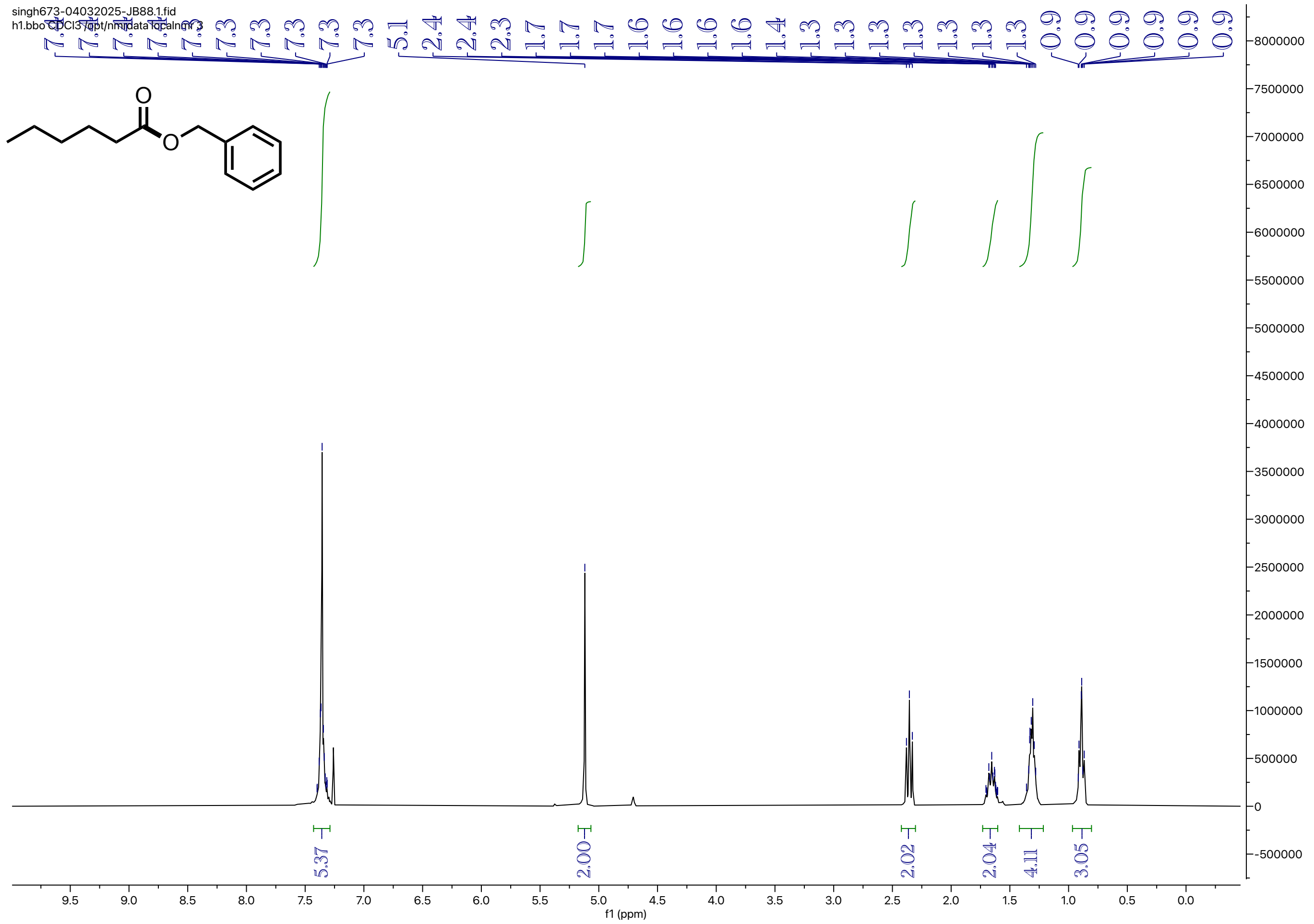

^{1}H NMR (300 MHz, Chloroform-*d*) benzyl hexanoate (**36**)

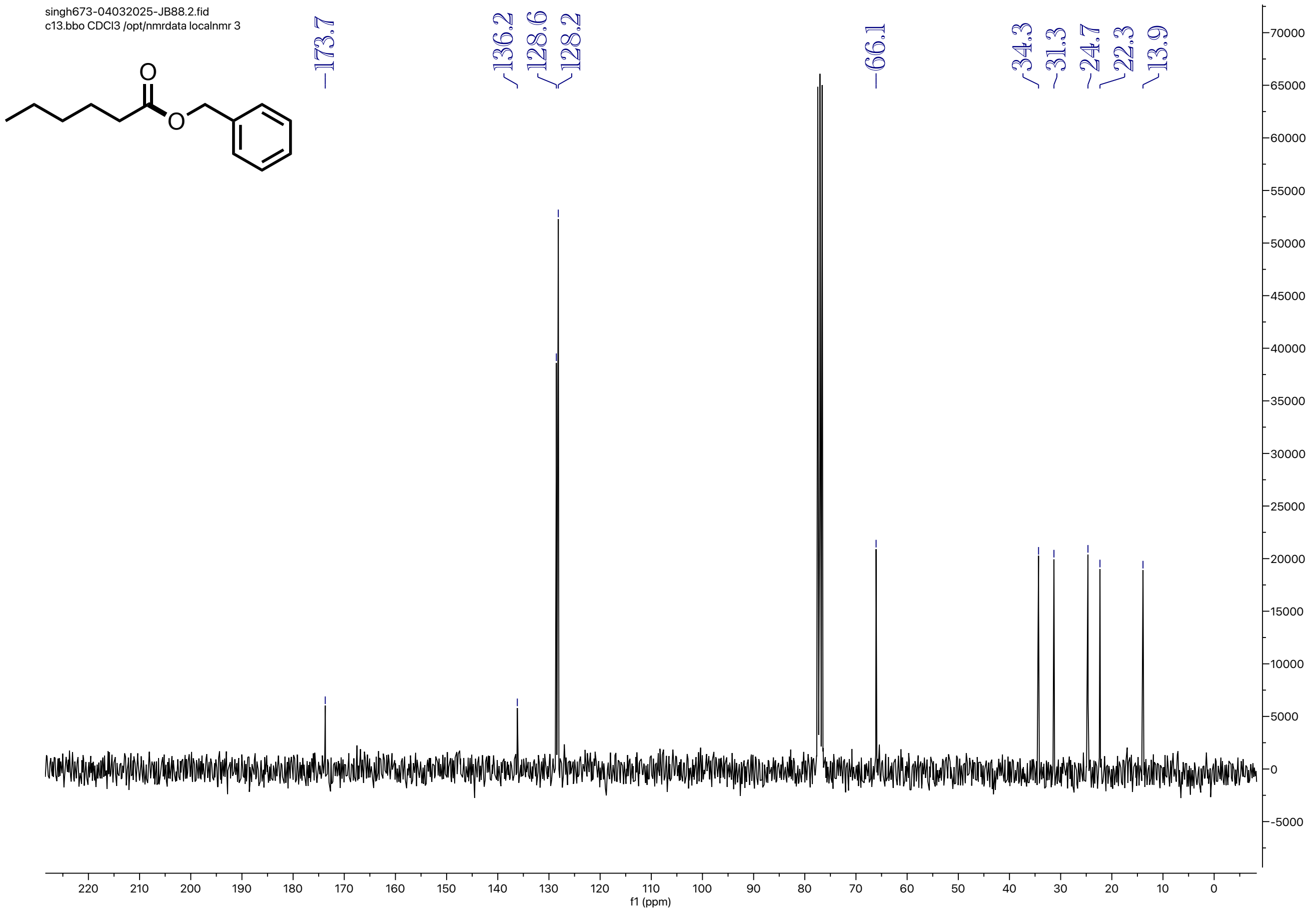

^{13}C {^{1}H} NMR (75 MHz, Chloroform-*d*) benzyl hexanoate (**36**)

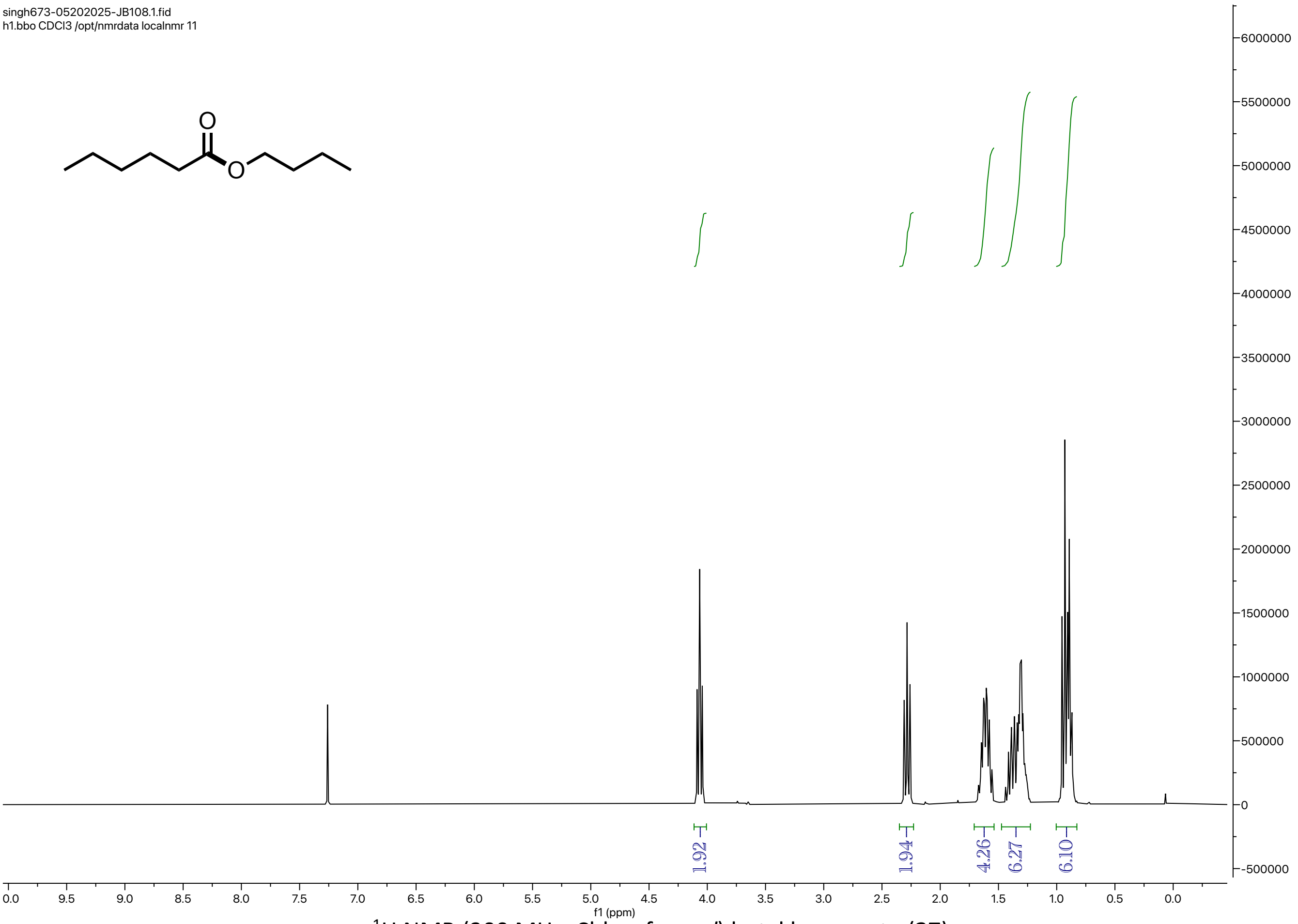

^{1}H NMR (300 MHz, Chloroform-*d*) butyl hexanoate (**37**)

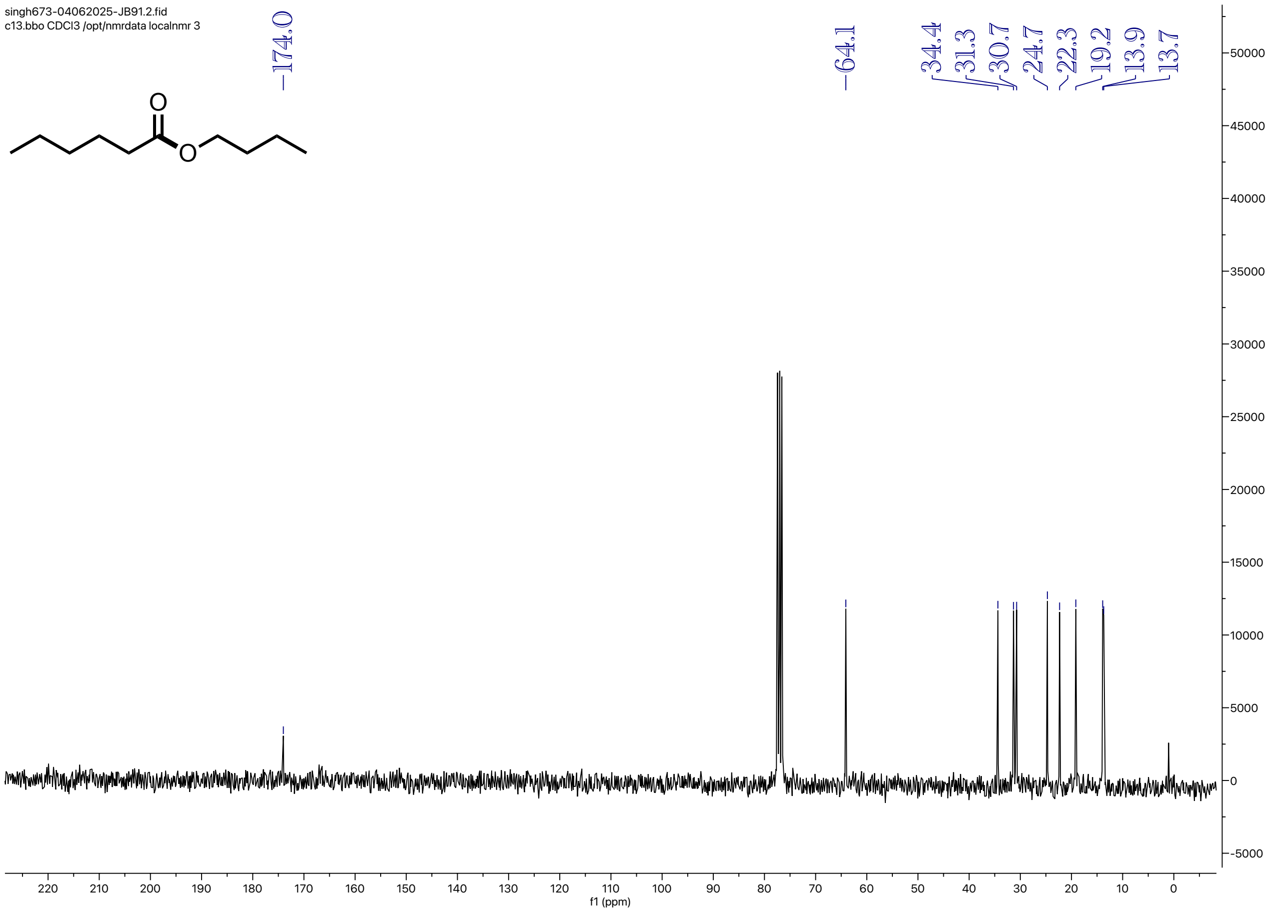

^{13}C {^{1}H} NMR (75 MHz, Chloroform-*d*) butyl hexanoate (**37**)

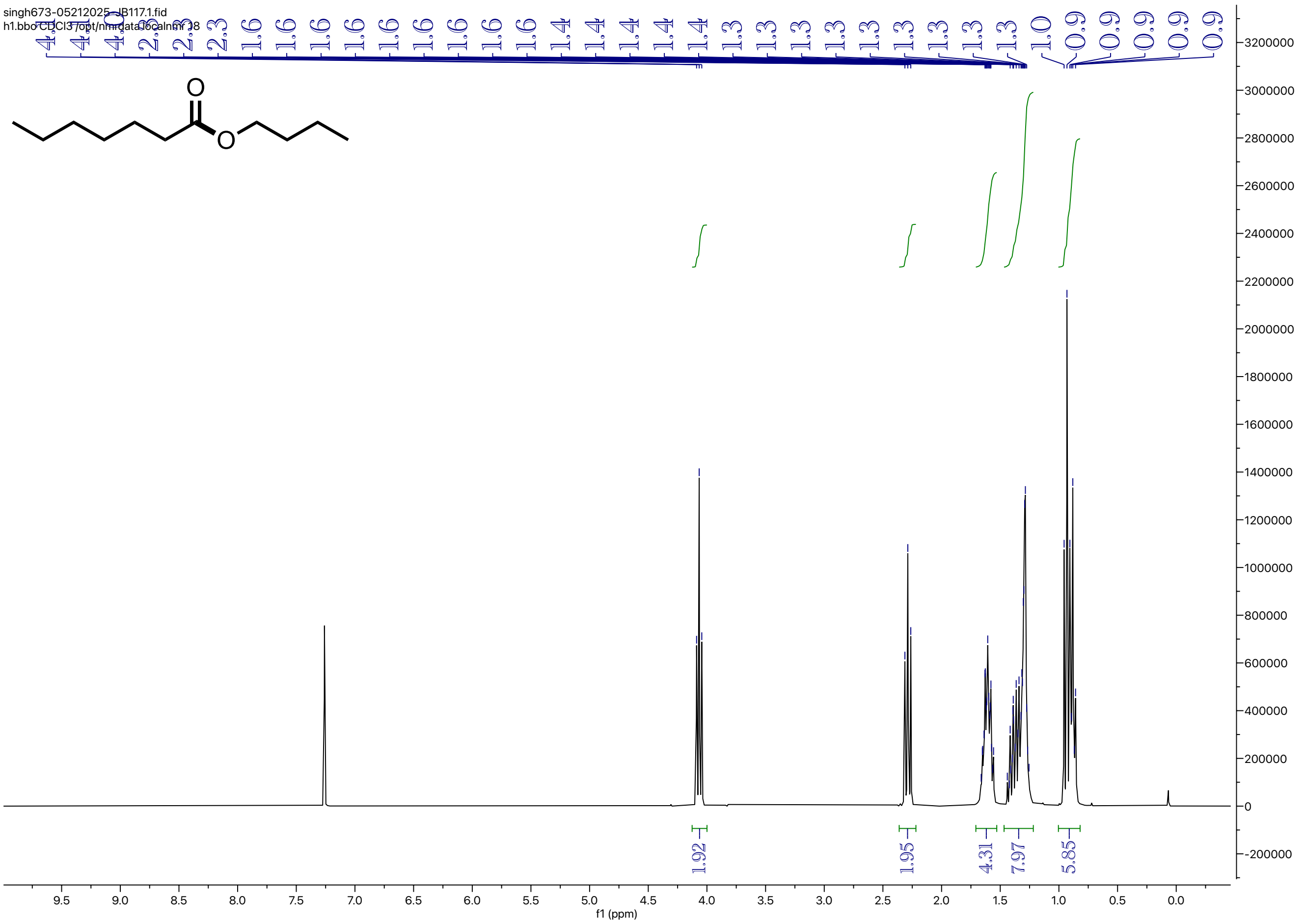

^{1}H NMR (300 MHz, Chloroform-*d*) butyl heptanoate (**38**)

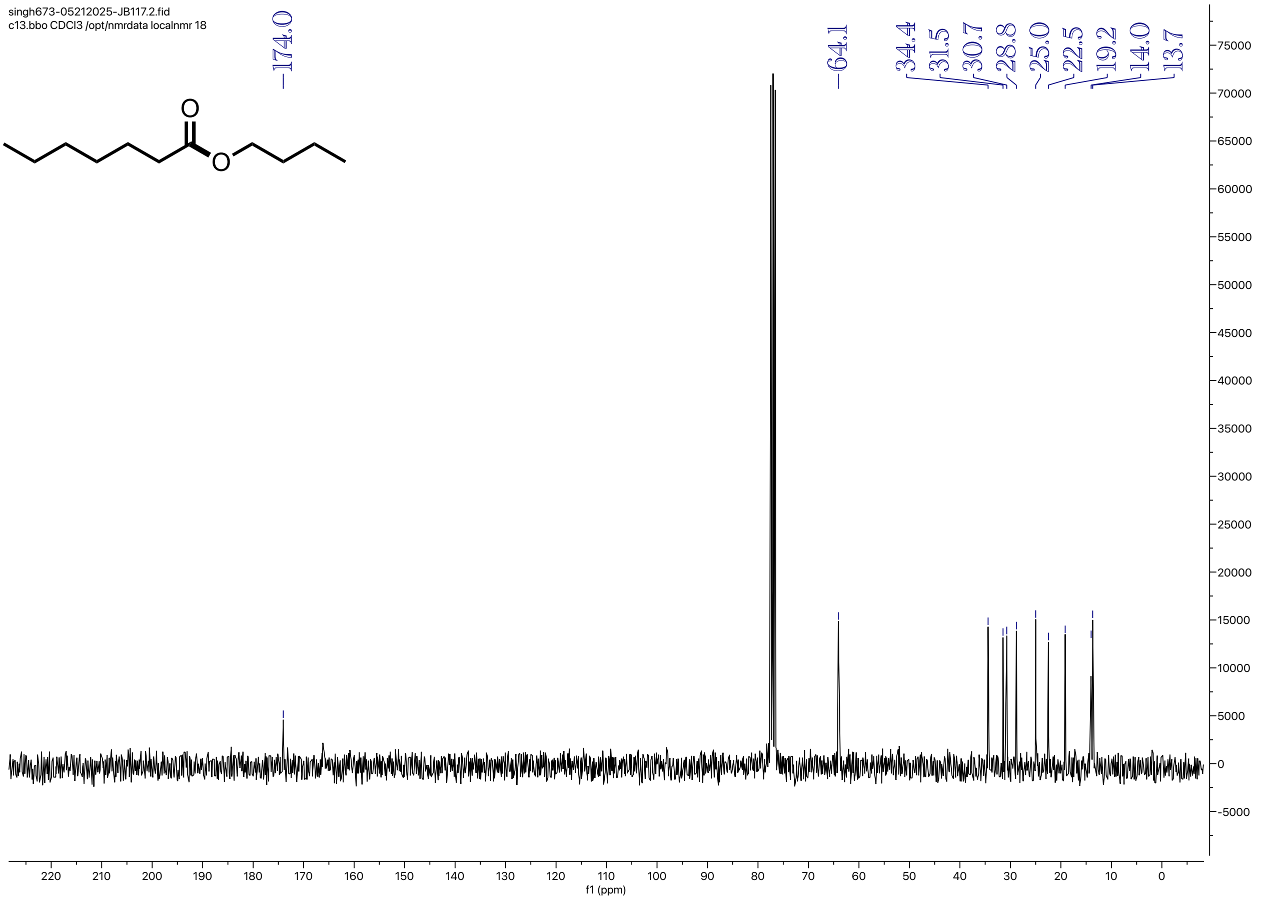

^{13}C NMR (75 MHz, Chloroform-*d*) butyl heptanoate (**38**)

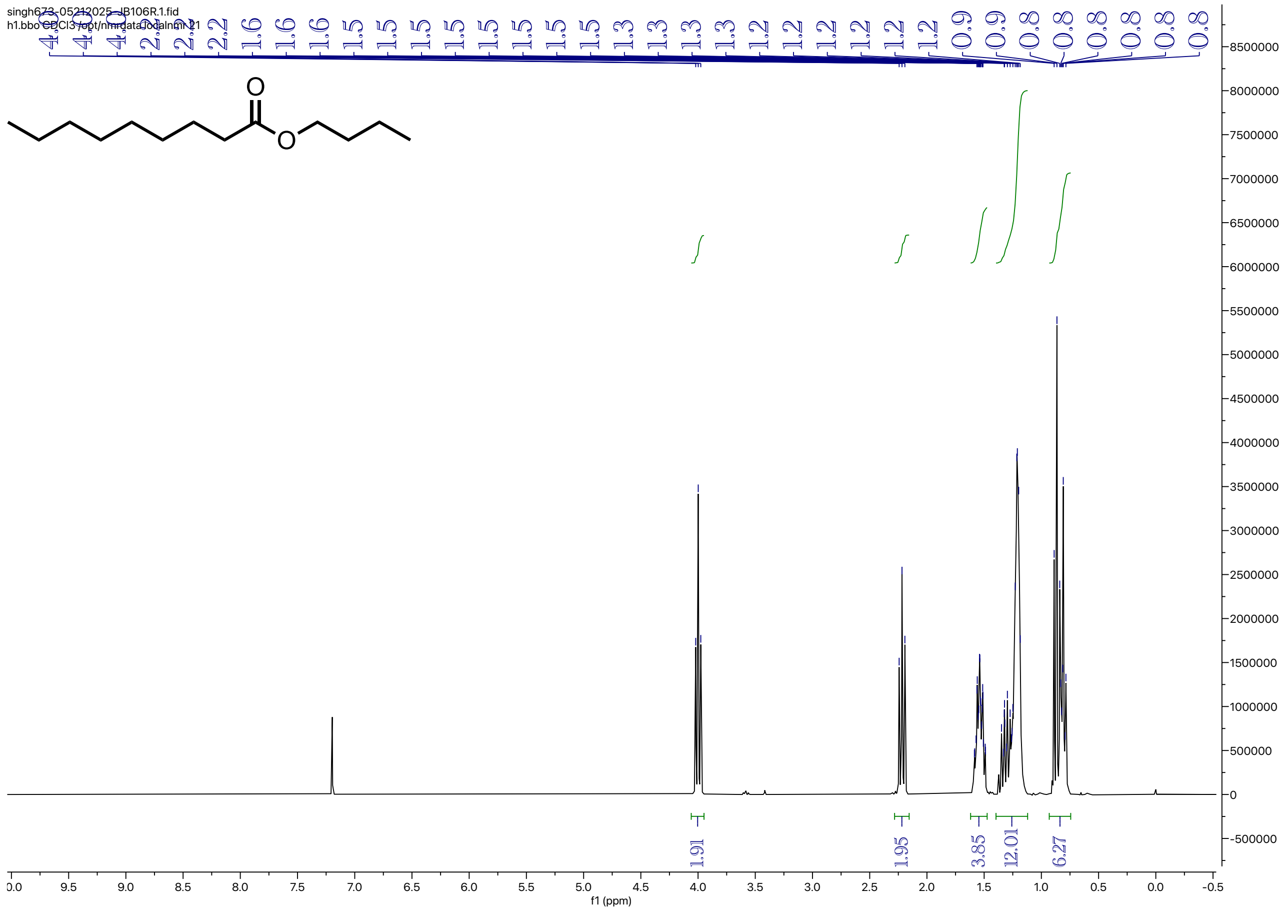

^{1}H NMR (300 MHz, Chloroform-*d*) butyl nonanoate (**39**)

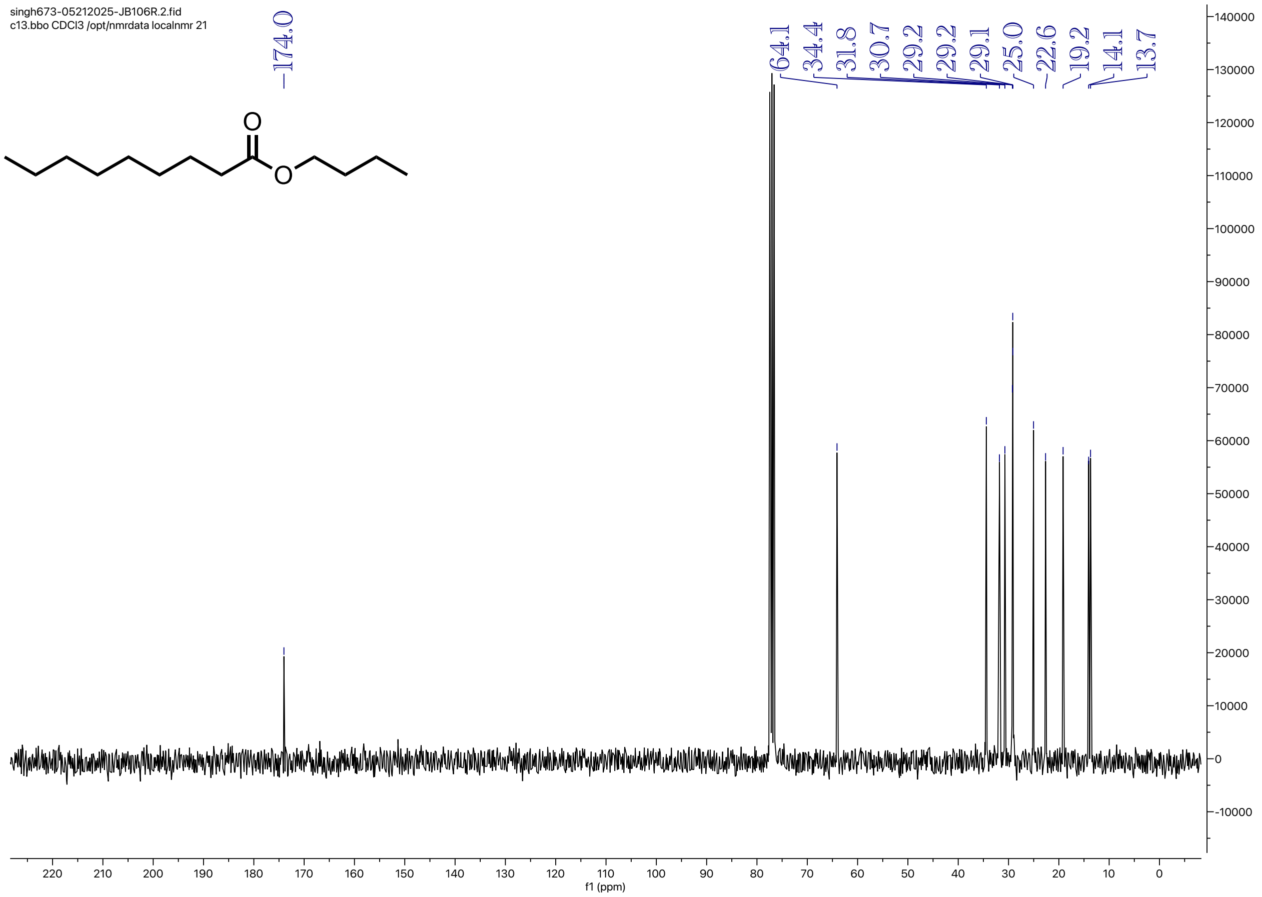

^{13}C NMR (75 MHz, Chloroform-*d*) butyl nonanoate (**39**)

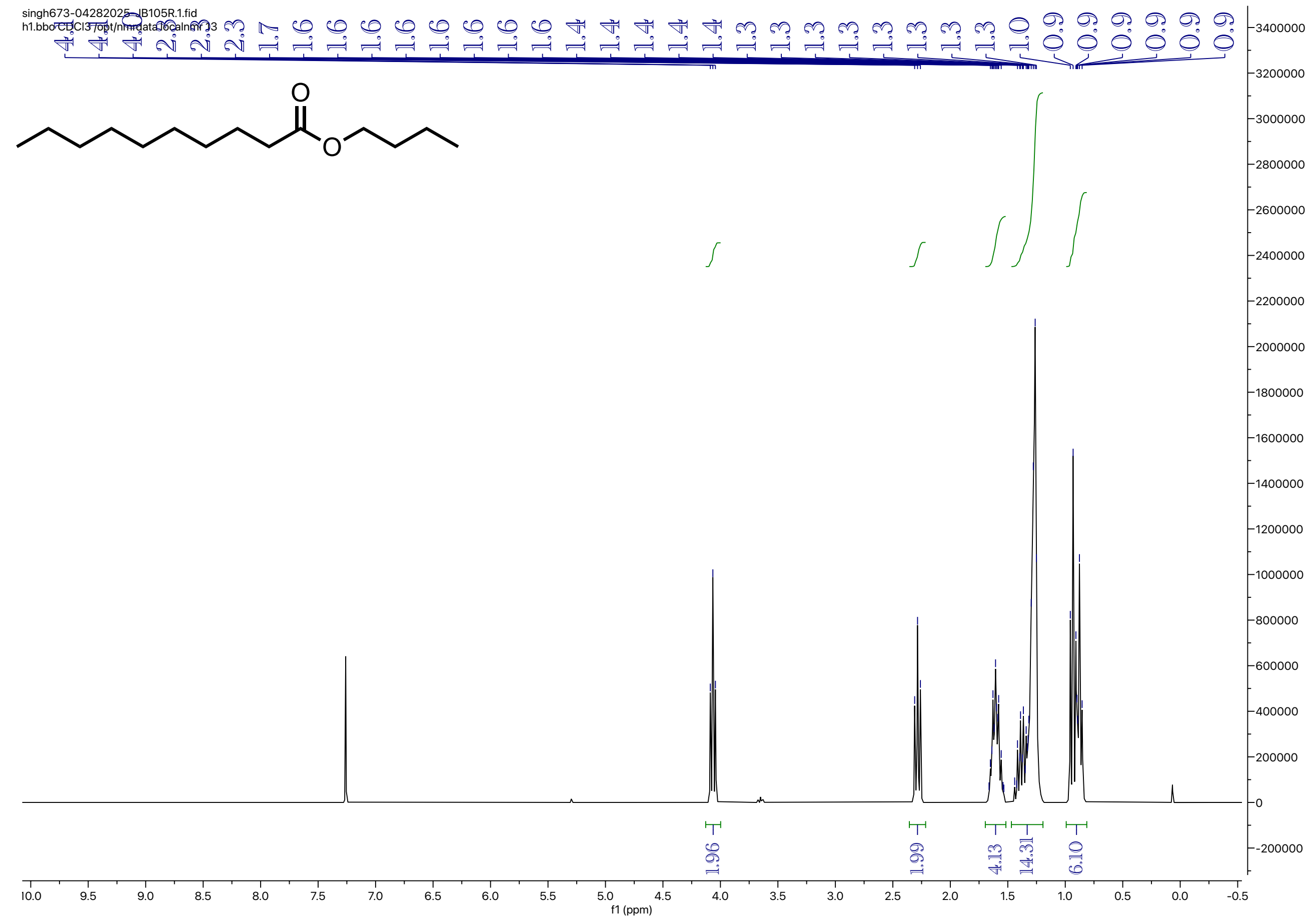

^{1}H NMR (300 MHz, Chloroform-*d*) butyl decanoate (**40**)

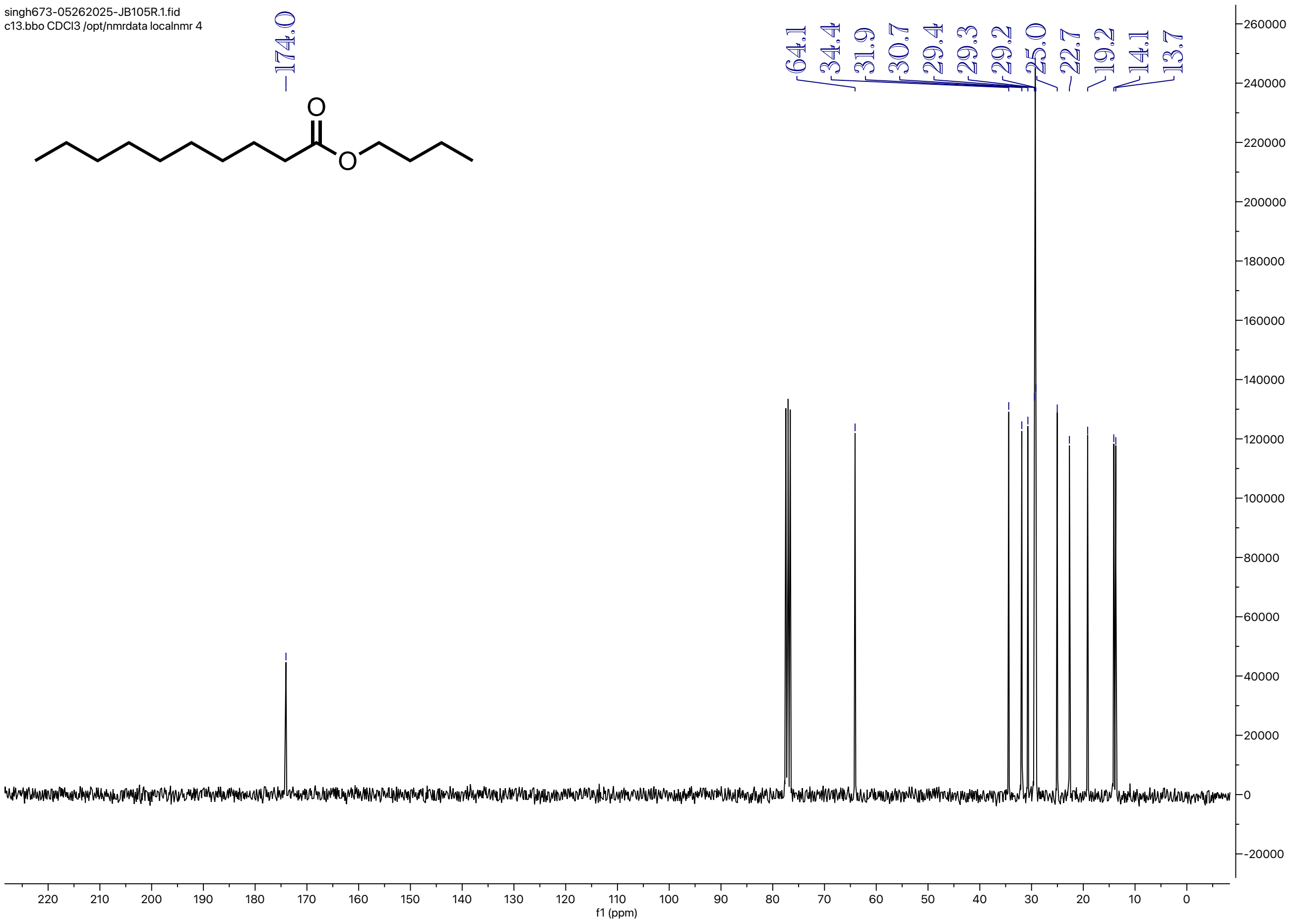

^{13}C NMR (75 MHz, Chloroform-*d*) butyl decanoate (**40**)

Green Chemistry Metrics:

Entry	Reagent 1	Reagent 2	Catalyst	Additive	Solvent	Product
1	Hexanoic Acid<br>**MW:** 116.2 g/mol<br>**Mass:** 0.697g	Benzylamine<br>**MW:** 107.2 g/mol<br>**Mass:** 0.535g	Cryolite	-	Xylenes	*N*-Benzylhexanamide<br>**MW:** 205.3 g/mol<br>**Mass:** 1.001g<br>**Yield:** 97%
2	Hexanoic Acid<br>**MW:** 116.2 g/mol<br>**Mass:** 0.116g	Benzylamine<br>**MW:** 107.2 g/mol<br>**Mass:** 0.107g	*Candida antarctica lipase B*	-	CPME	*N*-Benzylhexanamide<br>**MW:** 205.3 g/mol<br>**Mass:** 0.201g<br>**Yield:** 98%
3	Hexanoic Acid<br>**MW:** 116.2 g/mol<br>**Mass:** 0.116g	Benzylamine<br>**MW:** 107.2 g/mol<br>**Mass:** 0.107g	POM-Fe	Phenyl Silane<br>MW: 108.22g/mol<br>Mass: 0.032g (0.3 equiv.)	Toluene	*N*-Benzylhexanamide<br>**MW:** 205.3 g/mol<br>**Mass:** 0.133g<br>**Yield:** 65%

<u>Atom Economy:</u>

$$= \frac{Molecular\ Mass\ of\ desired\ product}{Molecular\ Mass\ of\ all\ reactants} \text{ x } 100$$

$$= \frac{205.3}{139.4+107.2} \text{ x } 100\% = 83.2\% \text{ (Entry 1)}$$

$$= \frac{205.3}{116.2+107.2} \text{ x } 100\% = 92\% \text{ (Entry 2)}$$

$$= \frac{205.3}{116.2+107.2+32.4} \text{ x } 100\% = 80.2\% \text{ (Entry 3)}$$

<u>Atom Efficiency:</u>

= %Yield x Atom Economy

= 97 * 0.83 = 80.5% (Entry 1)

= 98 * 0.92 = 90.2% (Entry 2)

= 65 * 0.80 = 52% (Entry 3)

Reaction Mass Efficiency:

$$= \frac{Mass\ of\ desired\ product}{Mass\ of\ all\ reactants} \times 100$$

$$= \frac{1.001}{0.697+0.535} = 81.3\% \text{ (Entry 1)}$$

$$= \frac{0.201}{0.116+0.106} = 90.1\% \text{ (Entry 2)}$$

$$= \frac{0.133}{0.116+0.107+0.032} = 52\% \text{ (Entry 3)}$$
